# Azologization and repurposing of a hetero-stilbene-based kinase inhibitor: towards the design of photoswitchable sirtuin inhibitors

**DOI:** 10.3762/bjoc.15.214

**Published:** 2019-09-16

**Authors:** Christoph W Grathwol, Nathalie Wössner, Sören Swyter, Adam C Smith, Enrico Tapavicza, Robert K Hofstetter, Anja Bodtke, Manfred Jung, Andreas Link

**Affiliations:** 1 Institute of Pharmacy, University of Greifswald, Friedrich-Ludwig-Jahn-Str. 17, 17489 Greifswald, Germany; 2 Institute of Pharmaceutical Sciences, University of Freiburg, Albertstr. 25, 79104 Freiburg, Germany; 3 Department of Chemistry and Biochemistry, California State University Long Beach, 1250 Bellflower Boulevard, Long Beach, CA, 90840 USA

**Keywords:** azo compounds, epigenetics, photoswitch, sirtuins, stilbenes

## Abstract

The use of light as an external trigger to change ligand shape and as a result its bioactivity, allows the probing of pharmacologically relevant systems with spatiotemporal resolution. A hetero-stilbene lead resulting from the screening of a compound that was originally designed as kinase inhibitor served as a starting point for the design of photoswitchable sirtuin inhibitors. Because the original stilbenoid structure exerted unfavourable photochemical characteristics it was remodelled to its heteroarylic diazeno analogue. By this intramolecular azologization, the shape of the molecule was left unaltered, whereas the photoswitching ability was improved. As anticipated, the highly analogous compound showed similar activity in its thermodynamically stable stretched-out (*E*)-form. Irradiation of this isomer triggers isomerisation to the long-lived (*Z*)-configuration with a bent geometry causing a considerably shorter end‐to‐end distance. The resulting affinity shifts are intended to enable real‐time photomodulation of sirtuins in vitro.

## Introduction

**Figure 1:**
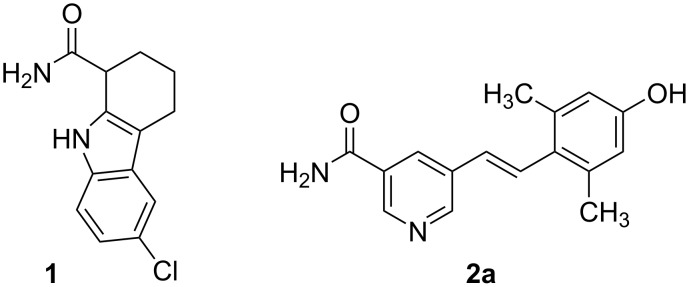
Selisistat (**1**) and hit compound GW435821X (**2a**).

Sirtuins are protein deacylases that cleave off not only acetyl, but also other acyl groups from the ε-amino group of lysines in histones and many other substrate proteins. This class of lysine deacetylases (KDACs) is distinguished from others by their dependence on the cosubstrate NAD^+^. In mammals, seven sirtuin isoforms have been identified to date [1]. These can be grouped into five classes (I, II, III, IV and V) according to their phylogenetic relationship [2]. The isoforms Sirt1, Sirt2 and Sirt3 originate from the same phylogenetic branch (class I), but differ in their subcellular localization. Although Sirt1 and Sirt2 were shown to shuttle between nucleus and cytoplasm in a cell-type and cell-cycle dependent manner, Sirt1 is mainly found in the nucleoplasm and Sirt2 in the cytoplasm [3–7]. Sirt3 primarily resides in the mitochondrion [8]. Facing the multitude of diseases that are associated with a dysregulation of sirtuin activity, they represent a promising target for pharmaceutical intervention. For example, selisistat (EX-527, **1**), a nanomolar and selective Sirt1 inhibitor, passed phase II clinical trials as a disease-modifying therapeutic for Huntington’s disease (HD) and was acquainted by AOP Orphan Pharmaceuticals AG for phase III trials in 2017 [9–10]. Its structure comprises a carboxamide moiety, which mimics the amide group of the endogenous pan-sirtuin inhibitor nicotinamide (Figure 1). Likewise Sirt2 inhibition was shown to have beneficial effects in animal and cell models of neurodegenerative diseases like HD and Parkinson’s disease [11–12]. Sirt3 activity recently was found to play an important role in cardiovascular diseases and extended ageing in humans [13–16]. Regarding tumorigenesis, the knowledge on the influence of sirtuins is inconsistent. Sirt1, Sirt2 and Sirt3 all have been reported to act either as tumor suppressors or promotors, depending on the particular cell type [1,17].

The ability to externally control the biological activity of small molecules in vitro or in vivo comprises numerous opportunities for example in the elucidation of biochemical pathways or the reduction of systemic side effects in drug therapy. Molecular photoswitches, i.e., compounds that undergo changes in their geometry and physicochemical properties upon irradiation with light, represent one major approach to this. One of the most common light-driven transformations exploited in molecular photoswitches is the *E*–*Z* isomerization of double bonds [18]. In this context, the photochemistry of stilbenes and the closely related azobenzenes has been studied intensely in the past [19–23]. Due to the multifaceted photoreactivity of unsubstituted stilbenes, an appropriate modification of the stilbene core is necessary to prevent unwanted irreversible side reactions [24–25]. On the contrary, the photochemical properties of azobenzenes are more convenient as already proven by their use as photoswitches in countless biological applications [26–30]. However, their heteroaromatic counterparts still seem underrepresented [31]. The approach to new chemotypes for sirtuin inhibition via known adenosine mimicking kinase inhibitors has already been fruitful in the past [32–33]. Therefore, a focused kinase inhibitor library from GlaxoSmithKline was screened for biological activity on human sirtuin isoforms Sirt1–Sirt3. Aza-stilbene derivative GW435821X (**2a,** Figure 1), initially published as c-RAF kinase inhibitor, was identified as a moderately active Sirt2 inhibitor with low selectivity [34–35]. In this work, the photoresponsiveness of the hetero-stilbene core structure is examined. Furthermore, an intramolecular azologization approach is performed in order to obtain photoswitchable sirtuin inhibitors, which could be useful tools in the further investigation of the biochemistry and pharmacology of sirtuins.

## Results

### Chemistry of azastilbenes

**Scheme 1:**
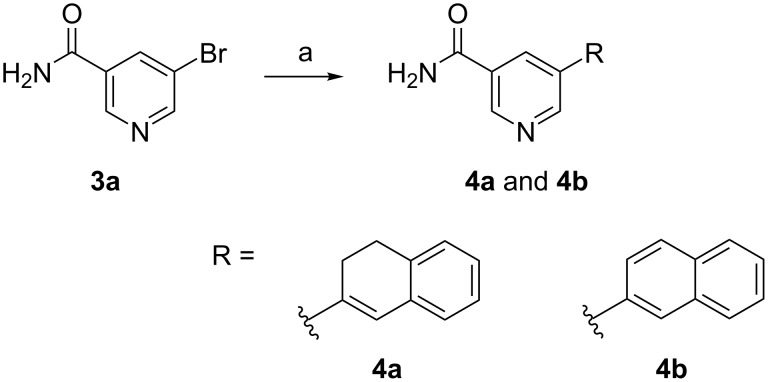
Reagents and conditions: a) appropriate boronic acid, Pd(PPh_3_)_4_, Na_2_CO_3_, DMF, H_2_O, microwave, 15 min, 150 °C, 43–64%.

All azastilbene derivatives were synthesised by palladium-catalysed cross-coupling reactions using either commercially available 5-bromonicotinamide (**3a**) or methyl 5-bromonicotinate (**3b**). If **3b** was used, transformation to the nicotinamide was accomplished almost quantitatively by addition of a saturated solution of ammonia in anhydrous methanol and stirring in a closed vessel at 40 °C. Compounds **4a** and **b** could easily be obtained through Suzuki coupling with commercially available naphthalene-2-ylboronic acid or (3,4-dihydronaphthalen-2-yl)boronic acid (Scheme 1). The latter was synthesized according to a literature procedure [36].

**Scheme 2:**
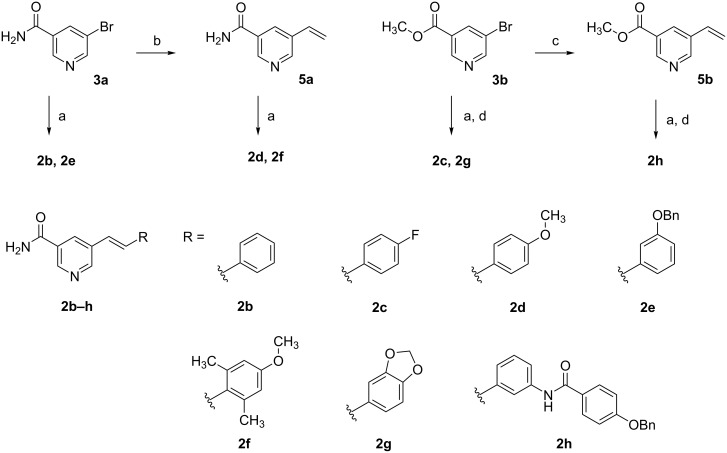
Reagents and conditions: a) Pd_2_(dba)_3_ or Pd(OAc)_2_, P(*o*-tol)_3_, TEA, DMF, 120–140 °C, 0.7–24 h, 11–75%; b) potassium vinyltrifluoroborate, Cs_2_CO_3_, PdCl_2_(PPh_3_)_2_, ACN, H_2_O, 1.5 h, 120 °C, 78% c) tributylvinyltin, Pd(PPh_3_)_4_; toluene, reflux, 3 h, 76%; d) NH_3_, MeOH, 40 °C, 3 d, 87–95%.

Formation of compounds **2b**–**h** was accomplished through Heck coupling of aryl bromides with the appropriate styrenes (Scheme 2) [37].

Compounds **2b** and **2e** were obtained in moderate yield using **3a** as the aryl halide in the Heck reaction. The use of **3b** in the Heck reaction resulted in a substantial improvement of yield in the synthesis of **2g** but not for **2c**. Interchanging the roles by using 5-vinylnicotinamide (**5a**) or methyl 5-vinylnicotinate (**5b**) as alkene component had detrimental effects on the yields in the synthesis of **2d**, **2f** and **2h**. Intermediates **5a** and **5b** were accessible from **3a** and **3b** via Suzuki–Miyaura or Stille coupling [34].

### Biology

The influence on deacetylase activity of three human sirtuin isoforms (Sirt1–3) was determined in a fluorescence-based assay, using *Z*-Lys(acetyl)-AMC (ZMAL) as a substrate [38]. Compared to the lead structure **2a**, all compounds except **2e**–**h** show increased inhibitory activity against Sirt2 (Table 1). Compound **2c** represents the most potent inhibitior with an IC_50_ value of about 7 µM. Moreover, a slight increase in selectivity for Sirt2 and Sirt3 over Sirt1 could be observed for **2c**, **4a** and **4b**. While none of the modifications provided complete isoenzyme specificity, **2c** preferentially inhibited Sirt2 (IC_50_ 6.6 ± 0.5) and Sirt3 (IC_50_ 7.5 ± 0.9 µM) compared to Sirt1 (51% inhibition at 100 µM). Though not photoswitchable, compounds **4a** and **4b** were synthesized to test the influence of a rigid conformation around the C=C double bond on sirtuin inhibition. Interestingly, this increased rigidity provokes a complete loss of activity against Sirt1. Despite the fact, that all mammalian sirtuins possess profound similarity in their catalytic core domains, many isotype selective inhibitors have been developed in recent years [39–45]. In the case of Sirt2 it was shown that appropriate ligand binding can induce conformational changes of the enzyme, revealing a so-called selectivity pocket, which allows for isotype-specific interactions [46]. A recently developed fluorescence polarization (FP)-based assay enables mapping of ligand binding to this specific binding site [35]. For **2a** an interaction with the selectivity pocket was already implied in the same work. Additionally performed docking studies proposed a binding mode in which **2a** mimics the nicotinamide residue of NAD^+^, whereas aromatic amino acid residues of the selectivity pocket stabilize the dimethylphenol ring [35]. As photoisomerization in stilbenes and azo dyes is accompanied by a perpendicular twist of the phenyl ring towards the former molecular plane, we assumed that this conformational change should provoke a differential binding situation at least for the dimethylphenol residue in **2a**. Unfortunately, binding of **2b** and **c** could not be localised in the vicinity of the selectivity pocket of Sirt2, so that the binding pose remains unclear.

**Table 1:** Sirt1–3 inhibition for compounds **2a–h**, **4a**/**4b** and **8a**.

Compound	Sirt1 inhibition^a^	Sirt2 inhibition^a^	Sirt3 inhibition^a^

**2a**	27% @ 50 µM	24.6 ± 2.8 µM^b^	41.7 ± 2.0 µM^b^
**2b**	71% @ 10 µM	8.7 ± 0.2 µM^b^	89% @ 50 µM
**2c**	51% @ 100 µM	6.6 ± 0.5 µM^b^	7.5 ± 0.9 µM^b^
**2d**	51% @ 10 µM	64% @ 10 µM	90% @ 50 µM
**2e**	61% @ 50 µM	69% @ 50 µM	60% @ 50 µM
**2f**	26% @ 10 µM	21% @ 10 µM	79% @ 50 µM
**2g**	52% @ 50 µM	62% @ 50 µM	87% @ 50 µM
**2h**	n.i.	9% @ 10 µM	n.i.
**4a**	n.i.	48% @ 10 µM	38% @ 10 µM
**4b**	n.i.	45% @ 10 µM	38% @ 10 µM
**8a**	n.i.	n.i.	n.i.

^a^Percent inhibition relative to controls at the indicated concentration, n.i. = no inhibition detected. ^b^IC_50_ values (μM) with statistical limits; values are the mean ± SD of duplicate experiments.

### Photochemistry of azastilbenes

The photochemical behaviour of stilbenes has been subject to intense investigation in the past. It is reported that unsubstituted stilbene undergoes *E*→*Z* photoisomerization [47], as well as photocyclization to dihydrophenanthrene upon UV irradiation, which is oxidized to phenantrene in the presence of oxygen [48]. In high concentrations, (*E*)-stilbene furthermore undergoes photocyclodimerization to cyclobutane derivatives [49]. Photoisomerization and photocyclization are also reported for 3-styrylpyridines, forming two regioisomeric dihydroazaphenanthrenes that are oxidized to 2- and 4-azaphenantrene (not shown), respectively [50].

Photochemistry of compounds **2b** and **2f** was investigated via UV–vis spectroscopy, LC–HRMS and NMR spectroscopy. Compound **2b** represents the core structure of the azastilbenes investigated, whereas in **2f** the influence of *ortho* methylation was intended to be examined. For UV–vis spectroscopy 50 µM solutions in 5% DMSO (v/v) in enzyme assay buffer were used, as this reflects the enzyme assay conditions. However, for LC-HRMS and NMR analysis, a higher concentration of 10 mM in methanol was necessary to receive reliable chromatograms and spectra.

**Figure 2:**
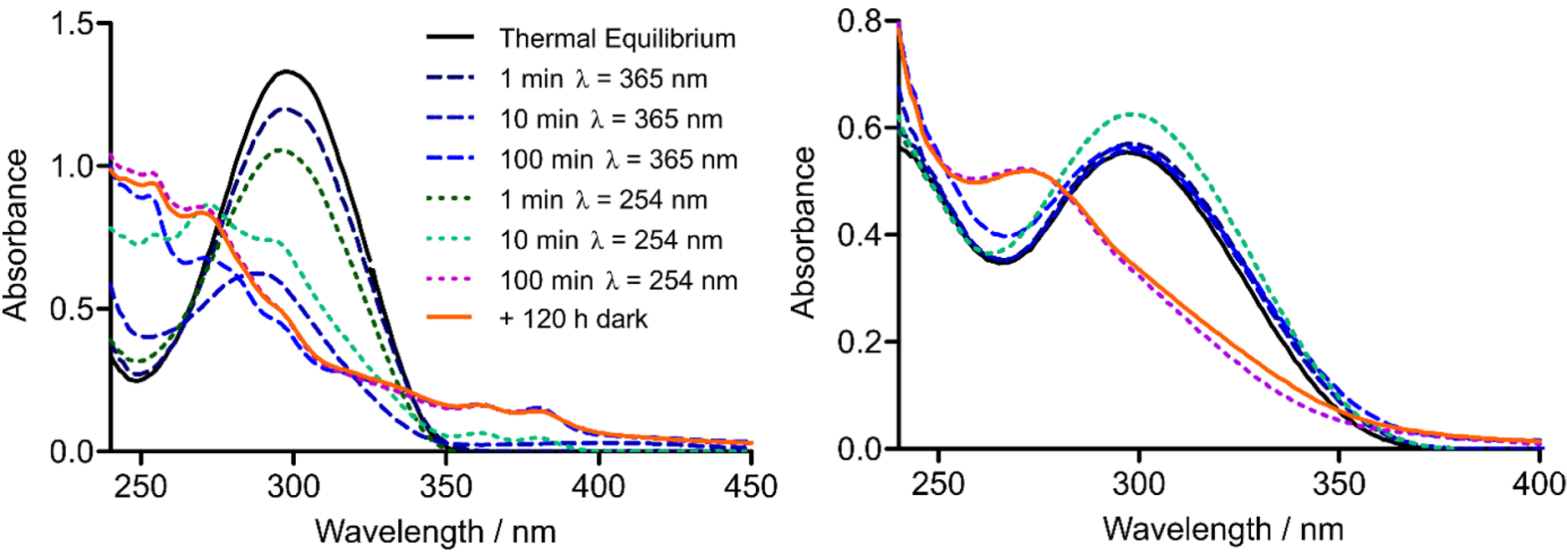
(Left) UV–vis spectrum of **2b** 50 µM in 5% DMSO (v/v) in assay buffer after varying durations of irradiation with 254 nm and 365 nm, respectively. (Right) UV–vis spectrum of **2f** 50 µM in 5% DMSO (v/v) in assay buffer after varying durations of UV radiation.

Upon exposure of **2b** to radiation of 365 nm, changes in the UV–vis spectra proceeded slowly, due to the low absorbance of **2b** in this wavelength region. However, shorter wavelengths, i.e. 254 nm, revealed fast and dramatic changes (Figure 2). After an initial decline and blue shift of the absorption maximum, the UV–vis spectrum of **2b** developed a more complex structure with further illumination. The initial spectrum did not restore, neither thermally by standing in the dark nor photochemically when exposed to daylight. Regarding **2f**, 254 nm radiation was obligatory to obtain changes in the UV–vis spectrum. However even long-term radiation did not lead to a complex spectrum as with **2b**, yet no stationary state was reached in the examined time. As in the case of **2b**, the spectrum of **2f** was not altered by daylight, nor by standing several days in the dark at room temperature.

**Figure 3:**
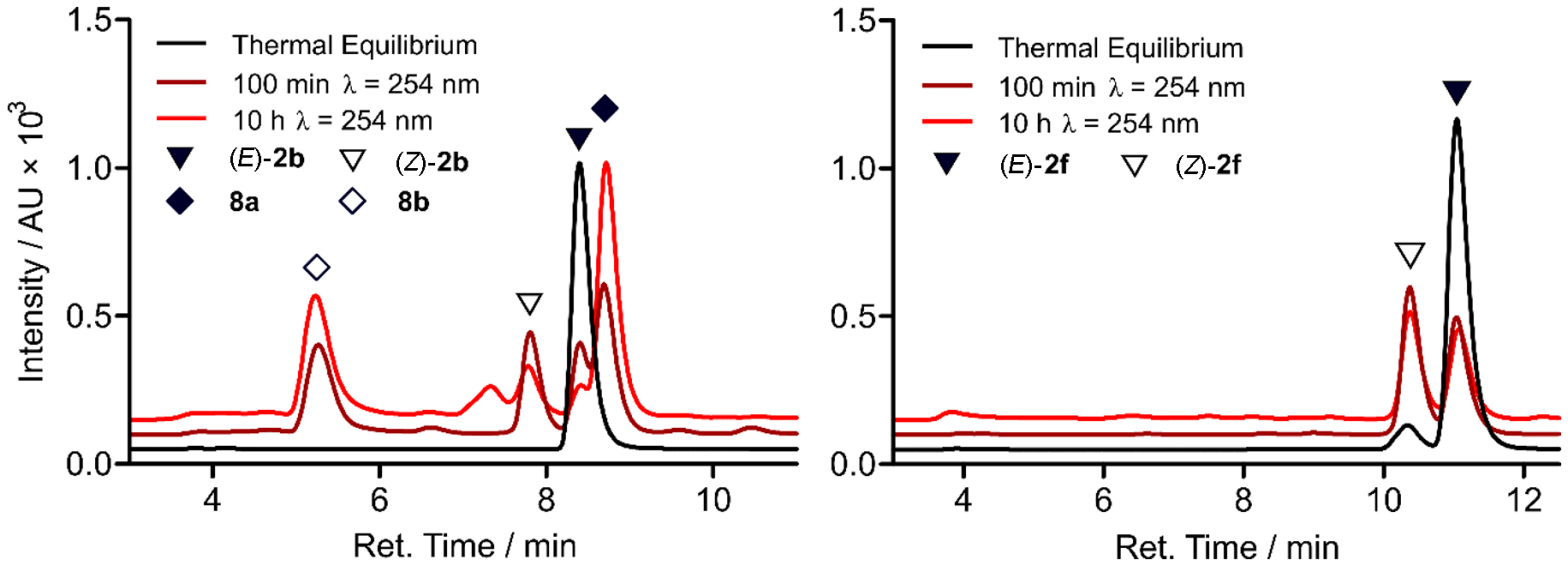
(Left) LC chromatogram of the LC–HRMS analysis of **2b** after varying durations of irradiation with 254 nm. Identity of **8a** was assigned by the reference compound synthesized and allowed differentiation of the two fractions containing photocyclized compounds, as indicated by mass spectra. (Right) LC chromatogram of the LC–HRMS analysis of **2f** after varying durations of irradiation with 254 nm.

LC–HRMS analysis provided deeper insights and clarified the differential behaviour observed in the UV–vis spectra of **2b** and **2f** after UV irradiation. As anticipated, UV irradiation lead to *E*→*Z* isomerization of the C=C double bond in both compounds. The (*Z*)-isomers were found to be slightly more polar than the respective (*E*)-isomers and their absorption maxima appeared blue shifted as demonstrated by the UV–vis spectra extracted from the LC runs. Unfortunately, the amount of photoisomerization was only moderate, since after 100 minutes of continuous irradiation still substantial amounts of the (*E*)-isomers were present in the mixtures (Figure 3). Proton NMR analysis implied photostationary states comprising a relative percentage of 45% (*Z*)-**2b** and 57% (*Z*)-**2f**, respectively after 100 minutes of 254 nm irradiation. The NMR spectra can be found in Supporting Information File 1.

**Scheme 3:**
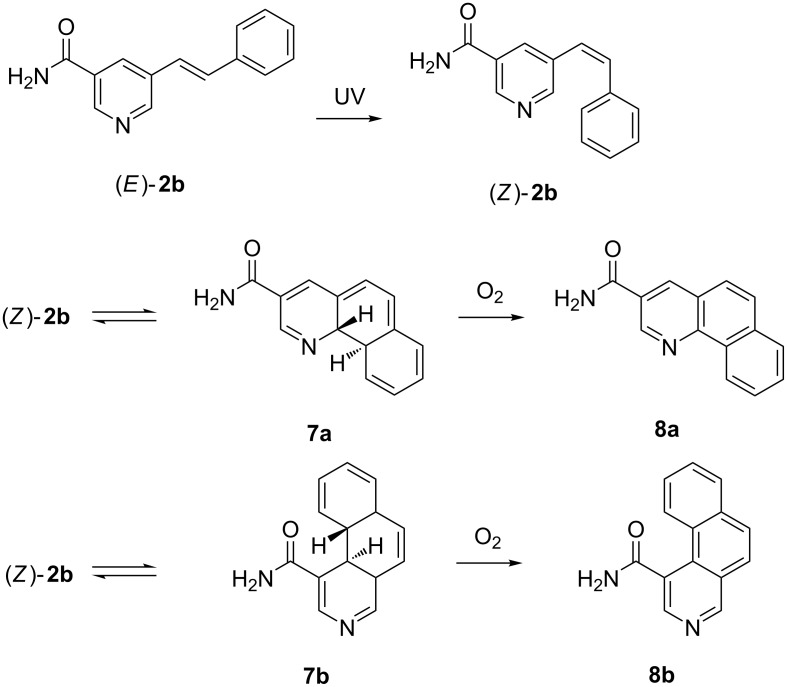
Photocyclization and oxidation reaction of **2b** upon UV irradiation.

The degree of photoisomerization could not be enhanced by extended illumination. Instead, for **2b** prolonged irradiation resulted in the formation of several side products, so that after 10 hours the fractions containing (*E*)-**2b** and (*Z*)-**2b** had declined significantly. This decrease was primarily accompanied by an increase of the fractions containing the benzoquinoline carboxamide isomers **8a** and **b** formed by photocyclization and successive oxidation (Scheme 3). Furthermore, small amounts of cycloaddition products in two fractions were found, probably due to the high concentration of **2b** in the irradiated solution. In contrast, **2f** was remarkably stable to long-term UV radiation. Even though the ratio of the double bond isomers was left unaffected, only small traces of the cycloaddition product and some unidentified compounds were registered. No formation of benzoquinoline carboxamides was registered as in the case of **2b**. Hence, due to the sterically blocking *ortho* methyl groups in **2f**, intramolecular photocyclization could be prevented.

**Figure 4:**
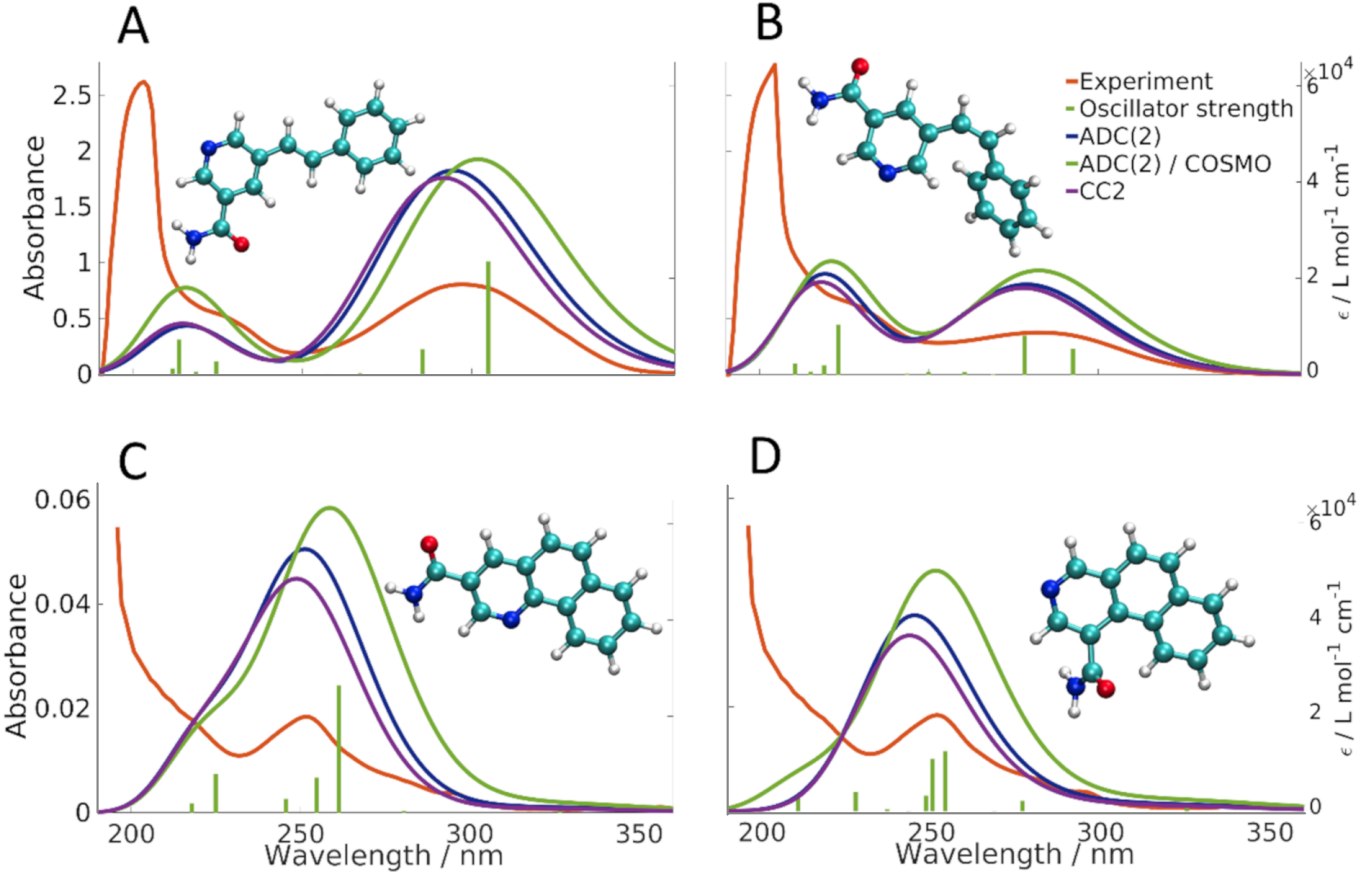
Calculated and experimental absorption spectra of compounds (*E*)-**2b-B** (A), (*Z*)-**2b-A** (B), and products **8a** (C) and **8b** (D). Oscillator strengths (green sticks) correspond to the ADC(2)/COSMO calculation.

To verify the hypothetical structures derived from irradiation of **2b**, we carried out quantum chemical calculations of the double bond isomers (*E*)-**2b** and (*Z*)-**2b** as well as the oxidized compounds **8a** and **8b**. We used density functional theory (DFT) to optimize the ground state equilibrium structures of (*E*)-**2b**, (*Z*)-**2b**, **8a** and **8b**, and used time-dependent DFT (TDDFT) and high-level correlated methods to obtain UV–vis absorption energies and oscillator strengths. To obtain the simulated absorption spectrum and λ_max_ values, oscillator strengths were converted into molar decadic extinction coefficients using a Gaussian line shape with a full-width-at-half-maximum of 0.3 eV. The correlated methods used were second-order approximated coupled cluster singles and doubles (CC2) and its approximation, algebraic diagrammatic construction to second-order (ADC(2)) [51–53]. ADC2 calculations have also been carried out with the implicit solvent continuum model COSMO using a dielectricity constant and refractive index of a methanol/water mixture, which was used as solvent in the experimental UV–vis measurements of the LC-HRMS fractions [54–55]. Geometries for reactants (*E*)-**2b** and (*Z*)-**2b** were optimized for two different rotational isomers ((*E*)-**2b-A** and (*E*)-**2b-B**; (*Z*)-**2b-A** and (*Z*)-**2b-B**), defined in Supporting Information File 1. In the following, we report only the results for (*E*)-**2b-B** and (*Z*)-**2b-A**, since they possess lower ground state energies and therefore are expected to be the dominant species at room temperature. Energy differences of the ground state structures of two pairs of isomers, however, are less than 0.6 kcal/mol, and computed spectra differ only slightly. Extensive results of all structures and all applied computational methods are summarized in the Supporting Information. While TDDFT systematically underestimates the λ_max_ values of the lowest absorption of all compounds by 0.1–0.75 eV, CC2 and ADC(2) agree with the λ_max_ values of the lowest absorption bands with a maximum deviation of 0.15 eV, similar to the previously determined accuracy [56]. We notice a good agreement between ADC(2) gas phase calculations with CC2 gas phase calculations, which justifies the usage of the approximate ADC(2) method. Comparing the calculated absorption spectra for (*E*)-**2b-B** and (*Z*)-**2b-A** to the experimental spectra obtained from LC-HRMS (Figure 4A,B), we see that all calculations consistently confirm the experimentally found blue shift of about 15 nm (0.22 eV) for the λ_max_ value of the lowest absorption band. Blue shifts predicted by CC2, ADC(2), ADC(2)/COSMO are 14, 16, and 20 nm, respectively. Consistent with the experimental spectra, all theoretical methods predict the maximum extinction of the lowest absorption band of (*Z*)-**2b** to approximately one half of the one of (*E*)-**2b**. Since the maximum error of the methods (0.15 eV) is smaller than the observed blue shift (0.22 eV), we conclude that the computed λ_max_ values are meaningful and clearly support the successful formation of the *Z*-isomer. Regarding the spectra of the photocyclization and oxidation products **8a** and **8b** (Figure 4C,D), theoretical methods predict the λ_max_ value of the lowest absorption bands within 8 nm (≈0.15 eV) of the value of the experimental spectrum of the LC–HRMS, clearly confirming the experimentally found blue shift of 0.75 eV and 0.54 eV compared to compounds (*E*)-**2b** and (*Z*)-**2b**, respectively. Also here, we conclude that the calculations clearly support the formation of compounds **8a** and/or **8b**. However, due to the similarity of the spectra of **8a** and **8b**, calculations do not allow to predict which of the two isomers was present in the respective fraction analysed.

Regarding the high similarity between **8a**/**8b** and selisistat, it was likely that these cyclized compounds could possess biological activity against sirtuins, too. On the other hand they resemble a fixed (*Z*)-configuration of the stilbene double bond. Therefore, comparison with **2b** could provide information concerning differential biological activity of the two photoisomers. By applying Mallory reaction conditions to a solution of **2b** in methanol utilizing oxygen and iodine as oxidants we were able to isolate a preparative amount of **8a** and tested it for its biological activity against Sirt1, Sirt2 and Sirt3. Surprisingly, **8a** showed complete inactivity towards all sirtuins tested (Table 1). Hence it can be assumed that *E*→*Z* photoisomerization in similar compounds lowers inhibitory strength accordingly.

### Synthesis and photochemistry of photoswitchable diazeno analogue

Even though the photochemical properties of *ortho* methylated azastilbenes like **2f** could be improved by preventing photocyclization, they were still unsuitable for the use as photoswitchable sirtuin inhibitors in the enzyme assay. The long irradiation periods that were necessary to obtain significant amounts of the (*Z*)-isomers did not permit switching of the inhibitors in the enzyme assay mixture, as the fluorescent substrate and the enzyme would be harmed by long-term UV radiation. We envisioned to replace the stilbene motive of selected stilbene **2c** by a diazeno group, because photoisomerization of azo dyes was anticipated to proceed fast and reversible by application of UV irradiation and visible light, respectively in this analogue.

**Scheme 4:**
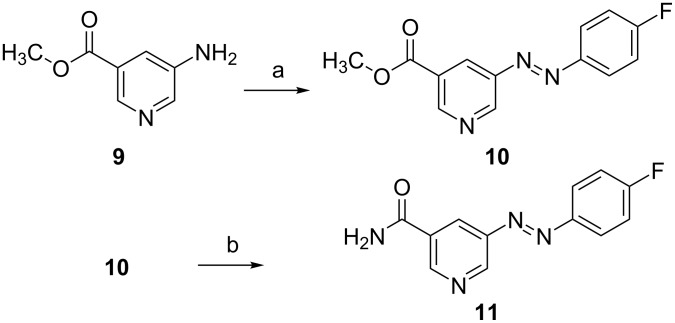
Reagents and conditions: a) 4-fluoroaniline, oxone, HAc, 60 °C, 14 d, 42%; b) NH_3_, MeOH, rt, 3 d, 98%.

5-Diazenylnicotinamide **11** was synthetically accessible in two steps through conversion of commercially available methyl 5-aminonicotinate (**9**) and 4-fluoroaniline to **10** under Mill’s reaction conditions and subsequent ammonolysis of the methyl ester **10** to amide **11** (Scheme 4).

**Figure 5:**
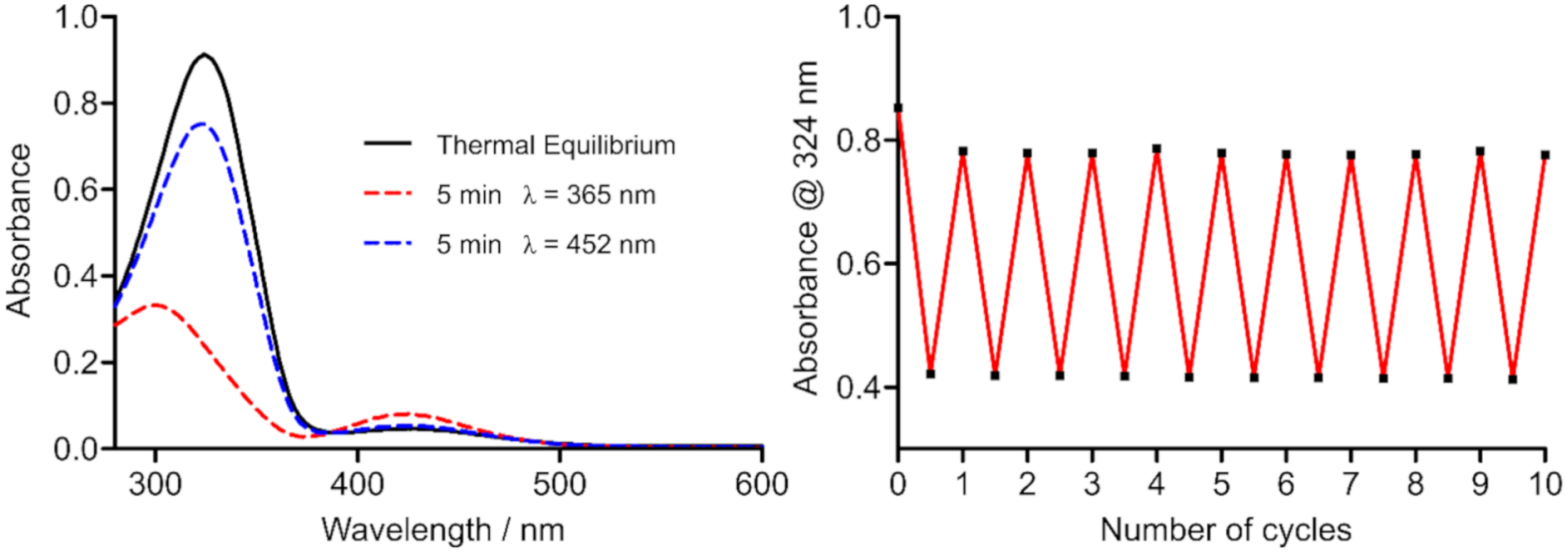
(Left) UV–vis spectrum of **11**, 50 µM in 5% DMSO (v/v), in assay buffer at the thermal equilibrium and the photostationary states (PSS) after 365 nm or 452 nm radiation. (Right) Fatigue resistance of **11**, 50 µM in 5% DMSO (v/v), in assay buffer over 10 cycles of alternating 365 nm and 452 nm radiation.

Photoswitching of (*E*)-**11** to a long-lived PSS (*t*_½_ = 300 h) containing 84% of (*Z*)-**11** was possible by short term UV irradiation of 365 nm. The photoisomerization could be reversed by exposure to visible light, i.e. 452 nm, albeit the PSS at 452 nm still comprised about 25% of (*Z*)-**11** as determined by HPLC analysis using UV–vis detection at the isosbestic points (Table 2). Light of 500 nm could also reverse photoisomerization, but was not as effective as 452 nm radiation. 630 nm irradiation, in contrast, did not lead to an altered PSS composition obtained by UV irradiation of 365 nm. Switching between the two PSS could be repeated several times without any observable fatigue of the compound (Figure 5).

**Table 2:** Percentage of *E*/*Z*-isomers of **11** at the thermal equilibrium (∆), and photostationary states (PSS) after 365 nm and 452 nm irradiation.

	∆	PSS 5 min 365 nm	PSS 1 min 452 nm

(*E*)-**11**/(*Z*)-**11**	99:1	16:84	75:25

The photoswitchable diazeno compound **11** was subjected to biological evaluation to test the effect of photoisomerization on the inhibitory activity. The enzyme assay mixture containing **11** was exposed to 5 minutes of 365 nm radiation and compared with the results of a non-irradiated measurement. The applied radiation did not perturb the proper enzyme functioning as proved by an unaltered enzyme activity in the blank tests. Unfortunately, 365 nm radiation turned out to have only minor effects on the IC_50_ values of **11** (Table 3).

**Table 3:** Sirt1-3 inhibition for compound **11** at the thermal equilibrium (∆) and the photostationary state (PSS) after 5 minutes of 365 nm irradiation.

Entry	Sirt1 inhibition^a^	Sirt2 inhibition^a^	Sirt3 inhibition^a^

∆	35% @ 100 µM	18.9 ± 1.38 µM	27.5 ± 3.42 µM
PSS	19% @ 100 µM	24.1 ± 1.69 µM	29.9 ± 2.11 µM

^a^Percent inhibition relative to controls at the indicated concentration, n.i. = no inhibition detected.

## Discussion

In recent years, photopharmacology has become a reputable strategy to optically control biochemical processes in the field of enzyme and ion channel modulation and recently 7TM-receptors also called GPCRs. Whereas in most approaches towards photoswitchable ligands the structure of the lead has to be changed considerable in order to incorporate a photoswitchable structural element, this was not the case with azastilbene-based lead structure **2a**. Unfortunately, due to several disadvantages the azastilbene moiety itself was unsuitable as photoswitchable element in this application. Even though competing azaphenantrene formation could be prevented by implementation of blocking *ortho* methyl groups in **2f**, the degree of photoisomerization in the two compounds observed was only moderate and required UV radiation over an extended period of time. Furthermore, the irreversibility of photoisomerization remained a major drawback and made an exchange with a diazeno group mandatory. Typically, it is not clear from the beginning, if the remodelling of the bioactive compounds will lead to an active diazeno derivative or not. The so-called azologization approach, moulded by Trauner et al., features a rational strategy for the design of photoswitchable compounds from established drug molecules through replacing certain core motives with an bioisosteric azobenzene moiety [57–59]. Recent examples have proven successful for receptor ligands by exchange of a linear alkinyl spacer for the zigzag shaped (*E*)-diazeno group [60–61]. In that instance, the geometry of the lead had to be changed considerably but careful design led to useful photoswitches. In the case of lead **2a** no such alteration of geometry was necessary and thus it seemed highly likely, that biological activity could be maintained. Indeed, this hypothesis could be proven. Exchange of the azastilbene double-bond with a diazeno bridge caused only a slight decrease in inhibitory potency against Sirt2 and Sirt3, and the selectivity profile of diazeno compound **11** equals the profile of its direct stilbene analogue **2c**. Concerning photoswitchability, **11** was superior to the stilbenoid structures, as it could be toggled reversibly between two states comprising high amounts of (*E*)-**11** and (*Z*)-**11**, respectively. The other part of the hypothesis was, that by this photoinduced isomerization a considerable drop of activity would occur due to the conformational change and the resulting changed geometry and polarity. However, this part of our hypothesis turned out to be wrong. The over-all conformational changes upon photoisomerization were too small or did not lead to a hindered binding, as anticipated. This result is disappointing, because the photoswitchable sirtuin inhibitor **11** cannot be switched between active and inactive state, as envisioned. Possible reasons could be assigned to substituent effects as demonstrated by Simeth et al. [62]. As recently reported by Schehr et al., reducing agents like DTT, used to prevent enzyme oxidation in crystallization mixtures or in vitro assays, can reduce azo dyes to hydrazine derivatives very fast and thus disable photoisomerization [63]. However, in our enzyme assay no such reducing agents were present, which is why we assume that the photoswitchable diazeno group should still be intact in the enzyme assay mixture. Even if the change in space orientation does not alter binding after irradiation, we would have predicted, that at least the significant difference in polarity of (*E*)-**11** and (*Z*)-**11** should lead to marked differences of sirtuin engagement in vitro. However, recent results from a carefully designed azologization study performed by Rustler et al. led to comparable difficulties [64].

## Conclusion

Based on lead structure GW435821X (**2a**) a small library of analogous azastilbene compounds was designed, synthesized and tested for their inhibitory activity against the human sirtuin isoforms Sirt1, Sirt2 and Sirt3. Compared to the lead structure the inhibitory potency could be increased to single digit µM potency for some compounds, while isoenzyme selectivity still remains an issue. The photochemistry of azastilbene compounds **2b** and **2f** was studied. For **2b**, besides photoisomerization, formation of benzoquinoline carboxamides by photocyclization and oxidation was indicated by high accuracy mass spectroscopy. Formation of 4-azaphenantrene derivative **8a** could be proven by isolation and characterization of a preparative sample. Theoretical UV–vis spectra for (*E*)-**2b**, (*Z*)-**2b** and two isomeric benzoquinoline carboxamides reproduced the experimental data. Compound **2f** was unsusceptible to photocyclization due to sterically blocking *ortho* methyl groups but could not be toggled between (*E*)- and (*Z*)-configuration. This lead to the synthesis of a first diazenyl derivative of the lead structure **2a** with promising photochemical characteristics for a new class of photoswitchable sirtuin inhibitors, but the activity difference for the (*E*)- and (*Z*)-isomers needs dramatic improvement before a useful molecular probe can be obtained by this approach.

## Experimental

### General remarks

All solvents and reagents were obtained from commercial suppliers and were used without purification. Anhydrous solvents were purchased from Acros Organics. Thin-layer chromatography (TLC) was executed on silica gel 60 F_254_ aluminium plates purchased from Merck. Visualization of the compounds was accomplished by UV-light (254 nm and 366 nm) and by staining with iodine, DNPH/H_2_SO_4_ (2 g 2,4-dinitrophenylhydrazine and 5 mL H_2_SO_4_ in 50 mL EtOH and 16 mL water) or vanillin/sulfuric acid (3.0 g vanillin and 0.5 mL H_2_SO_4_ in 100 mL EtOH) reagent. Synthesis was additionally monitored using high speed SFC/MS runs performed by a Nexera SFE-SFC/UHPLC switching system (Shimadzu Corporation, Kyoto, Japan) consisting of a pumping system (one LC-30ADSF for liquid CO_2_ and two LC-20ADXR for modifier and make-up delivery), an on-line supercritical fluid extraction module (SFE-30A auto extractor equipped with 0.2 mL extraction vessels) for reaction monitoring, an autosampler (SIL-30AC) for purified compounds, a column thermostat (CTO-20AC) equipped with a Torus DIOL (Waters) or Phenomenex CSP (Lux Amylose-2, i-Amylose-3, i-Cellulose-5), a degasser (DGU-20A5R), a communications module (CBM-20A), and two back pressure regulators BPR A and B (SFC-30A). UV and MS spectra were recorded via photodiode array detection (SPD-M20A) and electrospray ionization single quadrupole MS (LCMS-2020) controlled by Shimadzu LabSolution software (Version 5.91). Chromatographic purification of products was performed by flash chromatography on silica gel (20–45 µm, Carl Roth) applying pressured air up to 0.8 bar. NMR spectra were recorded on a Bruker Avance III instrument (^1^H NMR: 400 MHz, ^13^C NMR: 100.6 MHz). Chemical shifts were referenced to tetramethylsilane (TMS) as internal standard in deuterated solvents and reported in parts per million (ppm). Coupling constants (*J*) are reported in Hz using the abbreviations: s = singlet, d = doublet, t = triplet, q = quartet, m = multiplet and combinations thereof, br = broad. Infrared (IR) spectra were recorded on a Bruker Alpha FT-IR spectrometer equipped with a diamond ATR unit and are indicated in terms of absorbance frequency [cm^−1^]. Microwave synthesis was conducted in a Monowave 300 microwave synthesis reactor from Anton Paar equipped with appropriate sealed reaction vessels G10 (6 mL) or G30 (20 mL), applying a maximum initial power of 850 W to reach a given temperature (IR sensor) for a given time with stirring at 600 rpm. Melting points were measured in open capillary tubes using a Melting Point M-565 apparatus from Büchi and are uncorrected. High accuracy mass spectra were recorded on a Shimadzu LCMS-IT-TOF using ESI ionization. Purity of final compounds was determined by HPLC with DAD (applying the 100% method at 220 nm). Preparative and analytical HPLC were performed using Shimadzu devices CBM-20A, LC-20A P, SIL-20A, FRC-10A with SPD 20A UV–vis detector and an ELSD-LTII. In analytical mode a LiChroCART^®^ (250 × 4 mm) and in preparative mode a Hibar^®^ RT (250 × 25 mm) column, both containing LiChrospher^®^ 100 RP-18e (5 µm), were used. An Elementar Vario MICRO cube was used for the experimental determination of elemental configurations of final pure products. UV–vis spectra were obtained using a Thermo Scientific Genesys 10S UV–vis spectrophotometer.

### Synthesis

**General procedure for synthesis of nicotinamides from methyl nicotinates:** The respective methyl nicotinate was treated with a saturated solution of ammonia in anhydrous MeOH (30 mL) and stirred in a sealed vessel at 40 °C until thin layer chromatography indicated complete conversion of the starting material. The solvent was evaporated under reduced pressure and the residue washed sparingly with cold MeOH.

**(*E*)-5-Styrylnicotinamide (2b):** In a microwave reaction vessel **3a** (1.01 g, 5.00 mmol, 1.00 equiv) was mixed with styrene (651 mg, 6.25 mmol, 1.25 equiv), tris(*o*-tolyl)phosphine (61 mg, 0.20 mmol, 0.04 equiv), Pd_2_(dba)_3_ (92 mg, 0.10 mmol, 0.02 equiv) and NEt_3_ (863 ΜL, 0.63 g, 6.25 mmol, 1.25 equiv) and suspended in anhydrous DMF (6 mL). The reaction was conducted at 120 °C for 40 min in a microwave reactor. After cooling to room temperature the mixture was taken up in EtOAc and filtered through a pad of Celite^®^. The filtrate was washed with water (3 × 30 mL) and sat. aq. NaCl solution (30 mL), dried over MgSO_4_ and concentrated under reduced pressure. The formed precipitate was collected by filtration and recrystallized from EtOAc. The product was obtained as colourless crystals (0.55 g, 2.45 mmol, 49%): *R*_f_ = 0.25 (cyclohexane/THF 1:1); mp: 196.4 °C; ^1^H NMR (400 MHz, DMSO-*d*_6_) δ (ppm) 8.93 (d, *J* = 2.0 Hz, 1H), 8.90 (d, *J* = 2.1 Hz, 1H), 8.50 (pseudo-t, *J* = 2.0 Hz, 1H), 8.24 (s, br, 1H), 7.71–7.62 (m, 3H), 7.54–7.28 (m, 5H); ^13^C NMR, DEPT135, HSQC, HMBC (75.5 MHz, DMSO-*d*_6_) δ (ppm) 166.4, 150.4, 147.3, 136.4, 132.4, 131.4, 131.3, 129.6, 128.7, 128.2, 126.7, 124.2; IR (ATR) ν (cm^−1^): 3372, 3168, 1649, 1619, 1492, 1394, 961, 746, 691, 568; HRESIMS: calcd for [C_14_H_12_N_2_O + H]^+^ 224.0950, found 224.0939; comp. purity (220 nm): 100 %; anal. calcd for C_14_H_12_N_2_O: N, 12.49; C, 74.98; H, 5.39; found: N, 12.38; C, 74.81; H, 5.15.

**Methyl (*E*)-5-(4-fluorostyryl)nicotinate:** Synthesis was conducted according to the procedure of **2b** using **3b** (648 mg, 3.00 mmol, 1.00 equiv), 1-fluoro-4-vinylbenzene (550 mg, 4.50 mmol, 1.50 equiv), tris(*o*-tolyl)phosphine (183 mg, 0.60 mmol, 0.20 equiv), Pd_2_(dba)_3_ (67 mg, 0.30 mmol, 0.10 equiv) and NEt_3_ (1.25 mL, 9.00 mmol, 3.00 equiv) in anhydrous DMF (4 mL). The reaction was conducted at 140 °C for 1.5 h. The raw product was purified by silica gel column chromatography (*n*-hexane/EtOAc 2:1) yielding a colourless solid (97 mg, 0.38 mmol, 13%): *R*_f_ = 0.50 (*n*-hexane/EtOAc 2:1); mp: 108.2 °C; ^1^H NMR, H,H-COSY (400 MHz, CDCl_3_) δ (ppm) 9.09 (d, *J* = 1.8 Hz, 1H), 8.90 (d, *J* = 2.1 Hz, 1H), 8.52 (pseudo-t, *J* = 2.0 Hz, 1H), 7.57–7.49 (m, 2H), 7.26 (d, *J* = 16.4 Hz, 1H), 7.13–7.06 (m, 2H), 7.03 (d, *J* = 16.4 Hz, 1H); 4.00 (s, 3H, H-8); ^13^C NMR, DEPT135, HSQC, HMBC (75.5 MHz, CDCl_3_) δ (ppm) 165.3, 163.2 (d, *J* = 249.4 Hz), 150.1, 147.7, 135.0, 134.0, 135.7, 132.4 (d, *J* = 3.4 Hz), 132.2, 128.8 (d, *J* = 8.2 Hz), 126.9, 122.9 (d, *J* = 2.3 Hz), 116.2 (d, *J* = 21.8 Hz), 52.9; IR (ATR) ν (cm^−1^): 2957, 1718, 1508, 1433, 1299, 1230, 986, 821, 763; HRESIMS: calcd for [C_15_H_12_NO_2_F + H]^+^ 257.0852, found 257.0850.

**(*E*)-5-(4-Fluorostyryl)nicotinamide (2c):** Synthesis was conducted following the general procedure of nicotinamides from methyl nicotinates, using methyl (*E*)-5-(4-fluorostyryl)nicotinate (75 mg, 0.31 mmol, 1.00 equiv). The product was obtained as colourless solid (65 mg, 0.27 mmol, 87%): *R*_f_ = 0.48 (EtOAc/MeOH 95:5); mp: 205.6 °C; ^1^H NMR, H,H-COSY (400 MHz, DMSO-*d_6_*) δ (ppm) 8.91 (d, *J* = 1.9 Hz, 1H), 8.88 (d, *J* = 2.0 Hz, 1H), 8.47 (pseudo-t, *J* = 2.0 Hz, 1H), 8.22 (s, 1H), 7.76–7.68 (m, 2H), 7.49 (d, *J* = 16.6 Hz, 1H), 7.31 (d, *J* = 16.6 Hz, 1H), 7.29–7.22 (m, 2H); ^13^C NMR, DEPT135, HSQC, HMBC (75.5 MHz, DMSO-*d*_6_) δ (ppm) 166.4, 161.9, 150.3, 147.3, 133.1 (d, *J* = 3.2 Hz), 132.3, 131.4, 130.1, 129.7, 128.6 (d, *J* = 8.2 Hz), 124.1 (d, *J* = 2.2 Hz), 115.7 (d, *J* = 21.6 Hz); IR (ATR) ν (cm^–1^): 3364, 3172, 1650, 1620, 1507, 1397, 1212, 968, 857, 601; HRESIMS: calcd for [C_14_H_11_N_2_OF + H]^+^ 242.0855, found 242.0844; comp. purity (220 nm): 100%; anal. calcd for C_14_H_11_N_2_OF: N, 11.56; C, 69.41; H, 4.58; found: N, 11.53; C, 69.89; H, 4.51.

**Methyl 5-[(4-fluorophenyl)diazenyl]nicotinate (10):** 4-Fluoroaniline (444 mg, 4.00 mmol, 1.00 equiv) was dissolved in DCM (15 mL) and treated with a solution of oxone (4.92 g, 8.00 mmol, 2.00 equiv) in water (50 mL). The biphasic mixture was vigorously stirred until thin layer chromatography indicated complete consumption of the starting material. The watery phase was discarded and the organic phase washed with an aq. HCl-solution (1 M, 3 × 10 mL) and water (3 × 10 mL), then dried over MgSO_4_. The solution was concentrated to a volume of 5 mL under reduced pressure and added to a solution of **9** (609 mg, 4.00 mmol, 1.00 equiv) in acetic acid (20 mL). The reaction mixture was stirred at 60 °C for two weeks, cooled to room temperature, poured onto ice cooled sat. aq. NaHCO_3_-solution and extracted with EtOAc (3 × 50 mL). The combined organic extracts were washed with water (3 × 50 mL), sat. aq. NaCl solution (30 mL) and dired over MgSO_4_. The solvent was evaporated under reduced pressure and the residue purified by silica gel column chromatography (cyclohexane/EtOAc 3:1). The product was obtained as orange solid (431 mg, 1.67 mmol, 42%): *R*_f_ = 0.52 (cyclohexane/EtOAc 3:1); mp: 103.6 °C; ^1^H NMR, H,H-COSY (400 MHz, DMSO-*d*_6_) δ (ppm) 9.34 (d, *J* = 2.3 Hz, 1H), 9.22 (d, *J* = 2.0 Hz, 1H), 8.50 (pseudo-t, *J* = 2.2 Hz, 1H), 8.09–8.01 (m, 2H), 7.52–7.44 (m, 2H), 3.95 (s, 3H); ^13^C NMR, DEPT135, HSQC, HMBC (75.5 MHz, DMSO-*d*_6_) δ (ppm) 164.5, 164.4 (d, *J* = 251.8 Hz), 151.8, 150.3, 148.5 (d, J = 2.8 Hz), 146.7, 126.4 (d, *J* = 6.7 Hz), 125.4 (d, *J* = 9.5 Hz), 116.6 (d, J = 23.2 Hz), 52.7; IR (ATR) ν (cm^−1^): 3081, 1713, 1583, 1496, 1286, 1222, 1092, 1000, 843, 498.

**5-[(4-Fluorophenyl)diazenyl]nicotinamide (11):** Synthesis was conducted following the general procedure of nicotinamides from methyl nicotinates, using **10** (160 mg, 0.62 mmol, 1.00 equiv). The product was obtained as orange solid (149 mg, 0.61 mmol, 98%): *R*_f_ = 0.65 (EtOAc/MeOH 95:5); mp: 212.3 °C; ^1^H NMR, H,H-COSY (400 MHz, DMSO-*d*_6_) δ (ppm) 9.24 (d, *J* = 2.3 Hz, 1H), 9.20 (d, *J* = 2.0 Hz, 1H), 8.58 (pseudo-t, *J* = 2.2 Hz, 1H), 8.38 (s, br, 1H), 8.08–8.02 (m, 2H), 7.80 (s, br, 1H), 7.53–7.45 (m, 2H); ^13^C NMR, DEPT135, HSQC, HMBC (75.5 MHz, DMSO-*d*_6_) δ (ppm) 165.6, 164.3 (d, *J* = 251.4 Hz), 150.7, 148.6 (d, *J* = 2.8 Hz), 148.2, 146.7, 130.5, 125.7, 125.3 (d, *J* = 9.4 Hz), 116.6 (d, *J* = 23.2 Hz); IR (ATR) ν (cm^−1^): 3359, 3125, 1669, 1628, 1496, 1398, 1136, 838, 808, 692; HRESIMS: calcd for [C_12_H_9_N_4_OF + H]^+^ 224.0760, found 224.0753; comp. purity (220 nm): 100%; anal. calcd for C_12_H_9_N_4_OF: N, 22.94; C, 59.02; H, 3.71; found: N, 22.95; C, 59.46; H, 3.92.

**Cloning, expression and purification of recombinant proteins:** Expression and purification of Sirt1_133-747_, Sirt2_56−356_, and Sirt3_118−395_ was carried out as described previously. Identity and purity were verified by SDS-PAGE [65]. Protein concentration was determined by the Bradford assay [66]. Deacylase activity of sirtuin isotypes could be inhibited with nicotinamide and was shown to be NAD^+^-dependent.

**Bioassay:** The inhibitory effect of compounds **2a**–**h**, **4a**/**b**, **8a** and **11** on Sirt1–3 was detected via a previously reported fluorescence based assay [38]. The synthetic substrate *Z*-Lys(acetyl)-AMC (ZMAL) is deacetylated by sirtuins, followed by tryptic digestion and thereby release of 7-aminomethylcoumarin, leading to a fluorescent readout. Inhibition was determined by comparing percentage substrate conversion to a DMSO control after subtraction of the blank fluorescence signal. All compounds were tested at 100 µM, 50 µM and 10 µM, respectively. For compounds that showed more than 50% inhibition at 10 µM an IC_50_ value was determined. IC_50_ values were calculated with OriginPro 9.0 G using a non-linear regression to fit the dose response curve. An enzyme-free blank control and a 100% conversion control using AMC instead of ZMAL were measured as well. Inhibition measurements were performed in biological duplicates for all compounds.

**Photochemistry:** All photoisomerization experiments were conducted under ruby light of 630 nm. Illumination was executed using a Bio-Link 254 Crosslinker from Vilber-Lourmat equipped with six Vilber-Lourmat T8-C lamps (8 W, 254 nm) or six Vilber-Lourmat T8-L lamps (8W, 365 nm), respectively. Visible light radiation of 630 nm (red), 500 nm (green) and 452 nm (blue) was derived from a Paulmann FlexLED 3D strip. All compounds were irradiated in solution, using spectrophotometric grade solvents. Photoisomerization and UV–vis spectra measurement was conducted in quartz cuvettes at room temperature.

**Computational details:** All calculations were carried out using the TURBOMOLE version 7.2 quantum chemistry package [67]. Geometry optimizations of all compounds in different conformers were carried out using DFT with PBE approximation to the exchange-correlation (XC) functional and employing the SV(P) basis set [68–69]. The 10 lowest excitation energies and their oscillator strengths were computed using the SV(P) basis and the larger def2-TZVP basis set [69]. This was done using TDDFT with the hybrid approximation to the XC functional PBE0, CC2, and ADC(2) [51–53,70–72]. ADC(2) and CC2 calculations make use of the resolution-of-identity approximation [73]. ADC(2) calculations were also done using the continuum solvent model COSMO as previously described [54–55,74–76]. A dielectric constant of 62.14 and a refractive index of 1.3379 were used, which corresponds to a solvent of a 6/4-mixture of methanol/water, as experimentally determined [77–78]. Broadened absorption spectra were simulated by converting oscillator strengths to decadic extinction coefficients using a Gaussian line shape with a full-width-at-half-maximum of 0.3 eV [79–82].

## Supporting Information

The Supporting Information features experimental and analytical data for the synthesis of intermediates and compounds **4a**, **4b**, **2c–2h** and **8a** and ^1^H and ^13^C NMR spectra for all synthesized compounds. Procedures of photochemical experiments and their analysis are described. Detailed summaries of electronic structure calculations for two conformers (A and B) of each double bond isomer ((*E*)-**2b** and (*Z*)-**2b**), photocyclization and oxidation products **8a** and **8b** are given.

File 1 Experimental procedures, analytical data and quantum chemical calulations.
