# Supplementary material for: Azologization and repurposing of a hetero-stilbene-based kinase inhibitor: towards the design of photoswitchable sirtuin inhibitors
Source: Beilstein J Org Chem. 2019 Sep 16;15:2170–83. doi: 10.3762/bjoc.15.214 (PMC6774072; doi:10.3762/bjoc.15.214)
Supplement: File 1 — Experimental procedures, analytical data and quantum chemical calulations. [file Beilstein_J_Org_Chem-15-2170-s001.pdf]

BEILSTEIN JOURNAL OF ORGANIC CHEMISTRY

Supporting Information

for

Azologization and repurposing of a hetero-stilbene-based kinase inhibitor: towards the design of photoswitchable sirtuin inhibitors

Christoph W. Grathwol, Nathalie Wössner, Sören Swyter, Adam C. Smith, Enrico Tapavicza, Robert K. Hofstetter, Anja Bodtke, Manfred Jung and Andreas Link

Beilstein J. Org. Chem. **2019,** *15,* 2170–2183. doi:10.3762/bjoc.15.214

Experimental procedures, analytical data and quantum chemical calulations

License and Terms: This is a supporting information file under the terms of the Creative Commons Attribution License (http://creativecommons.org/licenses/by/4.0). Please note that the reuse, redistribution and reproduction in particular requires that the authors and source are credited.
The license is subject to the *Beilstein Journal of Organic Chemistry* terms and conditions: (https://www.beilstein-journals.org/bjoc)

General remarks **S3**

Experimental procedures and data **S3**

Synthesis of **4a** S4

Synthesis of **4b** S7

Synthesis of **2d** S7

Synthesis of **2e** S8

Synthesis of **2f** S9

Synthesis of **2h** S10

Synthesis of **2c** and **2g** S13

Synthesis of **8a** S15

NMR spectra **S16**

2-Bromo-3,4-dihydro-2*H*-naphthalen-1-one S16

2-Bromo-1,2,3,4-tetrahydronaphthalen-1-ol S17

3-Bromo-1,2-dihydronaphthalene S18

(3,4-Dihydronaphthalen-2-yl)boronic acid S19

5-(3,4-Dihydronaphthalen-2-yl)nicotinamide (**4a**) S20

5-(Naphthalen-2-yl)nicotinamide (**4b**) S21

(*E*)-5-Styrylnicotinamide (**2b**) S22

5-Vinylnicotinamide (**5a**) S23

(*E*)-5-(4-Methoxystyryl)nicotinamide (**2d**) S24

(*E*)-5-(4-Methoxy-2,6-dimethylstyryl)nicotinamide (**2f**) S25

1-(Benzyloxy)-3-vinylbenzene S26

(*E*)-5-[3-(Benzyloxy)styryl]nicotinamide (**2e**) S27

Methyl 5-vinylnicotinate (**5b**) S28

4-(Benzyloxy)-*N*-(3-bromophenyl)benzamide S29

Methyl (*E*)-5-{3-[4-(benzyloxy)benzamido]styryl}nicotinate S30

1-Fluoro-4-vinylbenzene S31

Methyl (*E*)-5-(4-fluorostyryl)nicotinate S32

5-Vinyl-1,3-benzodioxole S33

Methyl (*E*)-5-[2-(benzo-1,3-dioxol-5-yl)vinyl]nicotinate S34

(*E*)-5-(4-fluorostyryl)nicotinamide (**2c**) S35

(*E*)-5-[2-(benzodioxol-5-yl)vinyl]nicotinamide (**2g**) S36

(*E*)-5-{3-[4-(benzyloxy)benzamido]styryl}nicotinamide (**2h**) S37

Methyl 5-[(4-fluorophenyl)diazenyl]nicotinate (**10**) S38

5-[(4-Fluorophenyl)diazenyl]nicotinamide (**11**) S39

Benzo[*h*]quinoline-3-carboxamide (**8a**) S40

SFC setup S41

Irradiation setup S42

Determination of isosbestic points of 11 .... S43

PSS composition of 11 .... S43

PSS thermal half-life of 11 .... S46

Photochemistry of 2b and 2f S48

LCMS-IT-TOF analysis of **2b** S48

LCMS-IT-TOF analysis of **8a** S51

LCMS-IT-TOF analysis of **2f** S52

^{1}H NMR analysis of **2b** S55

^{1}H NMR analysis of **2f** S56

Computational details: S58

General remarks

All solvents and reagents were obtained from commercial suppliers and were used without purification. Anhydrous solvents were purchased from Acros Organics. Thin layer chromatography (TLC) was executed on silica gel 60 F_{254} aluminium plates purchased from Merck. Visualization of the compounds was accomplished by UV-light (254 nm and 366 nm) and by staining with iodine, DNPH/H_2SO_4 (2 g 2,4-dinitrophenylhydrazine and 5 mL H_2SO_4 in 50 mL EtOH and 16 mL water) or vanillin/sulfuric acid (3 g vanillin and 0.5 mL H_2SO_4 in 100 mL EtOH) reagent. Chromatographic purification of products was performed by flash chromatography on silica gel (20–45 µm, Carl Roth) applying pressured air up to 0.8 bar. NMR spectra were recorded on a Bruker Avance III instrument (^{1}H NMR: 400 MHz, ^{13}C NMR: 100.6 MHz). Chemical shifts were referenced to tetramethylsilane (TMS) as internal standard in deuterated solvents and reported in parts per million (ppm). Coupling constants (*J*) are reported in Hz using the abbreviations: s = singlet, d = doublet, t = triplet, q = quartet, m = multiplet and combinations thereof, br = broad. Infrared (IR) spectra were recorded on a Bruker Alpha FT-IR spectrometer equipped with a diamond ATR unit and are indicated in terms of absorbtion frequency [cm^{-1}]. Microwave synthesis was conducted in a Monowave 300 microwave synthesis reactor from Anton Paar equipped with appropriate sealed reaction vessels G10 (6 mL) or G30 (20 mL), applying a maximum initial power of 850 W to reach a given temperature (IR sensor) for a given time with stirring at 600 rpm. Melting points were measured in open capillary tubes using a Melting Point M-565 apparatus from Büchi and are uncorrected. High accuracy mass spectra were recorded on a Shimadzu LCMS-IT-TOF using ESI ionization. An Elementar Vario MICRO cube was used for the experimental determination of elemental configurations of final pure products. Preparative and analytical HPLC were performed using Shimadzu devices CBM-20A, LC-20A P, SIL-20A, FRC-10A with SPD 20A UV/Vis detector and an ELSD-LT Ⅱ. In analytical mode a LiChroCART® (250 × 4 mm) and in preparative mode a Hibar® RT (250 × 25 mm) column, both containing LiChrospher® 100 RP-18e (5 µm), were used. UV–vis spectra were obtained using a Thermo Scientific Genesys 10S UV-VIS spectrophotometer.

Experimental procedures and data

5-Vinylnicotinamide (**5a**)

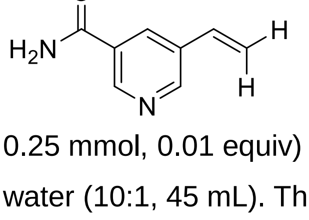

In an inert gas atmosphere **3a** (1.01 g, 5.00 mmol, 1.00 equiv), potassium vinyltrifluoroborate (871 mg, 6.50 mmol, 1.30 equiv), Cs_2CO_3 (4.50 g, 13,81 mmol, 2.76 equiv) and $PdCl_2(PPh_3)_2$ (175 mg, 0.25 mmol, 0.01 equiv) were mixed and suspended in a degassed mixture of acetonitrile and water (10:1, 45 mL). The reaction mixture was stirred at 120 °C for 1.5 h, then cooled to room

temperature and filtered through a pad of Celite®. The filtrate was taken up in EtOAc (30 mL) and aq. sat. NH_4Cl-solution (25 mL) and the phases separated. The watery phase was extracted with EtOAc (3 × 10 mL) and the combined organic phases washed with aq. sat. NaCl-solution (20 mL) and dried over $MgSO_4$. After evaporation of the solvent under reduced pressure, the residue was purified by silica gel column chromatography (EtOAc/MeOH 95:5). The product was obtained as yellow solid (570 mg, 3.85 mmol, 78%): R_f = 0.39 (EtOAc/MeOH 95:5); mp: 145.2 °C; **^{1}H NMR, H,H-COSY (400 MHz, DMSO-*d₆*):** δ (ppm) = 8.93 (d, *J* = 2.0 Hz, 1H), 8.79 (d, *J* = 2.1 Hz, 1H), 8.35 (pseudo-t, *J* = 2.1 Hz, 1H), 8.20 (s, br, 1H), 7.64 (s, br, 1H), 6.84 (dd, *J* = 17.7, 11.1 Hz, 1H), 6.08 (dd, *J* = 17.7, 0.5 Hz, 1H), 5.48 (d, *J* = 11.5 Hz, 1H); **^{13}C NMR, DEPT135, HSQC, HMBC (75.5 MHz, DMSO-d_6):** δ (ppm) = 166.3, 150.0, 147.9, 136.4, 132.9, 132.3, 131.5, 129.6, 117.6; **IR (ATR):** *v* (cm^{-1}) = 3342, 2987, 1677, 1614, 1590, 1385, 922, 902, 715, 610.

Synthesis of **4a**

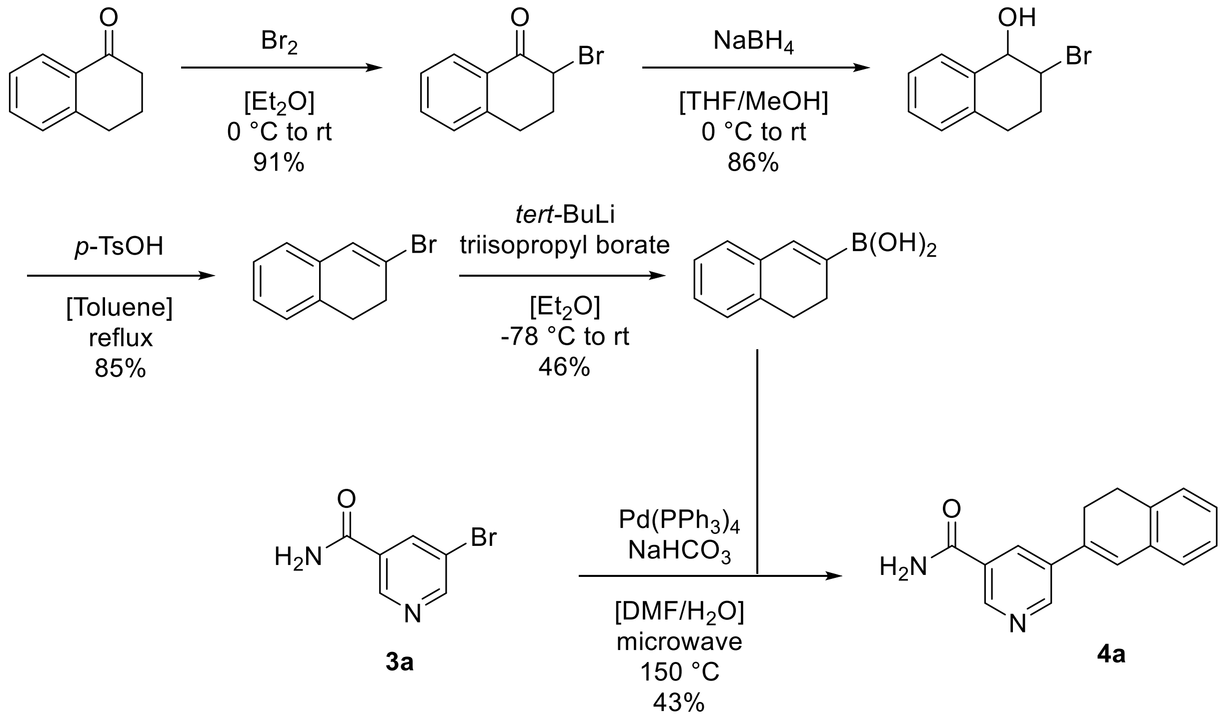

2-Bromo-3,4-dihydro-2*H*-naphthalen-1-one

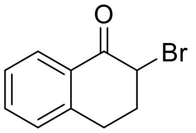

1-Tetralone (14.62 g, 100.0 mmol, 1.00 equiv) was dissolved in Et_2O (60 mL) and cooled to 0 °C. Bromine (17.58 g, 110.0 mmol, 1.10 equiv) was added dropwise under stirring. After complete addition stirring was continued at room temperature until TLC indicated complete consumption of the starting material. The reaction mixture was poured onto water (100 mL) and the aqueous phase was discarded. The organic phase was washed with aq. $Na_2S_2O_3$-solution (5%, 100 mL), water (100 mL) and sat. aq. NaCl-solution (100 mL). The organic phase was separated and dried over Na_2SO_4. The

solvent was evaporated and the residue purified by fractional vacuum distillation (b.p.: 110–112 °C, 0.02 mbar). The product was obtained as yellow oil, which solidified on standing at 4 °C. (20.55 g, 91.30 mmol, 91%): R_f = 0.53 (cyclohexane/EtOAc 4:1); mp: 28.0 °C; **^{1}H NMR, H,H-COSY (400 MHz, DMSO-*d6*):** δ (ppm) = 7.95 (dd, *J* = 8.2, 1.4 Hz, 1H), 7.65–7.59 (m, 1H), 7.44–7.37 (m, 2H), 5.05 (dd, *J* = 6.1, 3.6 Hz, 1H), 3.17–2.92 (m, 2H), 2.65–2.54 (m, 1H), 2.42–2.32 (m, 1H); **^{13}C NMR, DEPT135, HSQC, HMBC (75.5 MHz, DMSO-*d6*):** δ (ppm) = 190.1, 143.3, 134.1, 129.5, 129.0, 127.5, 127.0, 52.1, 31.6, 25.9; **IR (ATR):** *v* (cm^{-1}) = 2943, 1676, 1595, 1452, 1304, 1284, 1192, 886, 796, 736.

2-Bromo-1,2,3,4-tetrahydronaphthalen-1-ol

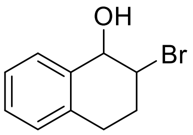

2-Bromo-3,4-dihydronaphthalen-1(2*H*)-one (4.50 g, 20.00 mmol, 1.00 equiv) was dissolved in a mixture of anhydrous MeOH and THF (1:2, 30 mL), set under an argon atmosphere and cooled to 0 °C. $NaBH_4$ (0.53 g, 14.01 mmol, 0.70 equiv) was added in small portions. After complete addition the mixture was stirred at 0 °C for 15 min followed by stirring at room temperature until TLC indicated complete conversion of the starting material. The reaction mixture was poured onto water (100 mL) and extracted with Et_2O (3 × 50 mL). The combined extracts were washed with sat. aq. NaCl-solution (2 × 50 mL) and dried over Na_2SO_4. The solvent was evaporated and the residue recrystallized from hexanes under usage of active charcoal. The product was obtained as slightly ochre crystals. (3.89 g, 17.12 mmol, 86%): R_f = 0.62 (cyclohexane/EtOAc 4:1); mp: 70.7 °C; **^{1}H NMR, H,H-COSY (400 MHz, DMSO-*d6*):** δ (ppm) = 7.38–7.33 (m, 1H), 7.21–7.13 (m, 2H), 7.10–7.05 (m, 1H), 5.37 (s, br, 1H), 4.74–4.66 (m, 2H), 2.99–2.75 (m, 2H), 2.37–2.20 (m, 1H); **^{13}C NMR, DEPT135, HSQC, HMBC (75.5 MHz, DMSO-*d6*):** δ (ppm) = 137.9, 134.2, 128.1, 128.0, 127.0, 125.7, 68.8, 58.2, 27.9, 27.0; **IR (ATR):** *v* (cm^{-1}) = 3386, 2946, 1489, 1455, 1429, 1312, 1058, 814, 744, 524.

3-Bromo-1,2-dihydronaphthalene

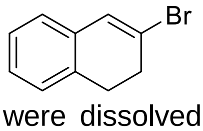

2-Bromo-1,2,3,4-tetrahydronaphthalen-1-ol (2.27 g, 10.00 mmol, 1.00 equiv) and *p*-toluenesulfonic acid monohydrate (190 mg, 1.00 mmol, 0.10 equiv) were dissolved in toluene (50 mL) and refluxed for 1 h. The solution was cooled to room temperature, washed successively with aq. $NaHCO_3$-solution (10 %, 3 × 50 mL) and sat. aq. NaCl-solution (50 mL), then dried over Na_2SO_4. The solvent was evaporated and the crude residue purified by silica gel column chromatography (hexanes), which yielded the product as slightly yellow oil. (1.70 g, 8.47 mmol, 85 %): R_f = 0.86 (hexanes); **^{1}H NMR (400 MHz, $CDCl_3$):** δ (ppm) = 7.17–7.10 (m, 2H), 7.09–7.04 (m, 1H), 6.98–6.92 (m, 1H), 6.78 (s, br, 1H), 4.74–4.66 (m, 2H), 2.99–2.75 (t, *J* = 8.2 Hz, 2H), 2.37–2.20 (m, 2H); **^{13}C NMR, DEPT135, HSQC, HMBC (75.5 MHz, $CDCl_3$):** δ (ppm) = 133.9, 132.9, 129.4, 127.5, 127.4, 126.7, 125.6, 124.1, 33.8, 29.2.

(3,4-Dihydronaphthalen-2-yl)boronic acid

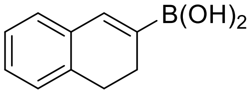

In an inert gas atmosphere 2-bromo-1,2,3,4-tetrahydronaphthalen-1-ol (1.71 g, 8.18 mmol, 1.00 equiv) was dissolved in anhydrous Et_2O (90 mL) and cooled to -78 °C. A solution of *tert*-butyllithium in pentane (1.7 M, 9.80 mL, 16.67 mmol, 2.04 equiv) was added dropwise under stirring so that the reaction temperature did not exceed -65 °C. After complete addition stirring was continued for another 30 min at −78 °C, then triisopropyl borate (3.13 g, 16.64 mmol, 2.03 equiv) was added dropwise. After addition cooling was ceased so that the reaction solution could reach room temperature. Aq. HCl-solution (3 M, 25 mL) was added and after 15 min of stirring, the organic phase was separated, dried over Na_2SO_4 and concentrated. The product was precipitated through addition of *n*-hexane and obtained as a colourless solid after filtration and drying. (657 mg, 3.78 mmol, 46 %): R_f = 0.38 (DCM/MeOH 99:1); mp: 207.4 °C; **^{1}H NMR, H,H-COSY (400 MHz, $CDCl_3$):** δ (ppm) = 7.61 (s, br, 1H), 7.27–7.07 (m, 4H), 2.83 (t, *J* = 8.1 Hz, 2H), 2.61–2.34 (m, 2H); **^{13}C NMR, HSQC, HMBC (75.5 MHz, $CDCl_3$):** δ (ppm) = 143.2, 137.8, 133.8, 132.3, 128.6, 127.7, 127.5, 126.5, 27.6, 23.3; **IR (ATR):** *v* (cm^{-1}) = 3204, 2929, 1613, 1566, 1372, 1340, 1287, 1210, 752, 694; **ESI-HRMS:** calcd. for $[C_{10}H_{11}BO_2\text{-}H]^-$ 173.078, found 173.057.

5-(3,4-Dihydronaphthalen-2-yl)nicotinamide (**4a**)

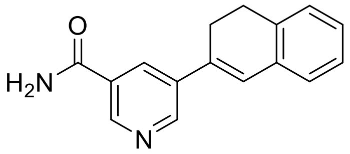

In a microwave reaction vessel **3a** (234 mg, 1.17 mmol, 1.00 equiv), (3,4-dihydronaphthalen-2-yl)boronic acid (226 mg, 1.30 mmol, 1.11 equiv) and $Pd(PPh_3)_4$ (75 mg, 0.065 mmol, 0.05 equiv) were mixed together and suspended in DMF (2 mL). After addition of $NaHCO_3$ (328 mg, 3.90 mmol, 3.33 equiv) in water (3 mL) the mixture was reacted at 150 °C for 15 min. After cooling to room temperature EtOAc (25 mL) and water (25 mL) were added and the catalyst removed by filtration through a pad of Celite®. The watery phase was extracted with EtOAc (3 × 10 mL) and the combined organic phases washed with water (3 × 20 mL) and sat. aq. NaCl-solution (25 mL), then dried over $MgSO_4$. After evaporation of the solvent the residue was recrystallized from acetone and obtained as colourless solid (125 mg, 0.50 mmol, 43%): R_f = 0.36 (hexanes/acetone 1:1); mp: 217.7 °C; **^{1}H NMR (400 MHz, DMSO-*d$_6$*):** δ (ppm) = 8.97 (d, *J* = 1.8 Hz, 1H), 8.93 (d, *J* = 1.5 Hz, 1H), 8.39 (pseudo-t, *J* = 2.1 Hz, 1H), 8.24 (s, br, 1H), 7.65 (s, br, 1H), 7.30–7.12 (m, 5H), 2.93 (t, *J* = 8.1 Hz, 1H), 2.76 (t, *J* = 8.1 Hz, 1H); **^{13}C NMR, DEPT135, HSQC, HMBC (75.5 MHz, DMSO-*d$_6$*):** δ (ppm) = 166.4, 148.4, 147.3, 135.2, 134.5, 134.5, 133.7, 131.0, 129.3, 127.6, 127.2, 126.9, 126.6, 125.9, 27.1, 24.9; **IR (ATR):** *v* (cm^{-1}) = 3363, 3193, 1672, 1623, 1397, 1140, 891, 778, 751, 610; **ESI-HRMS:** calcd. for $[C_{16}H_{14}N_2O+H]^+$ 250.1106, found 250.1097; **Comp. Purity (220 nm):** 100 %; **Elem. Anal.:** calcd. for $C_{16}H_{14}N_2O$: N 11.19, C 76.78, H 5.64, found: N 10.90, C 76.72, H 5.38.

Synthesis of **4b**

5-(Naphthalen-2yl)nicotinamide (**4b**)

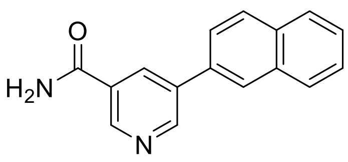

Synthesis was conducted according to the procedure of **4a** using **3a** (100 mg, 0.50 mmol, 1.00 equiv), naphthalen-2-ylboronic acid (112 mg, 0.65 mmol, 1.30 equiv), $Pd(PPh_3)_4$ (29 mg, 0.025 mmol, 0.05 equiv) in DMF (3 mL), together with $NaHCO_3$ (126 mg, 1.50 mmol, 3.00 equiv) in water (2 mL). The reaction was conducted at 150 °C for 15 min in a microwave reactor. The product was obtained as colourless crystals (79 mg, 0.32 mmol, 64%): R_f = 0.37 (hexanes/acetone 1:1); mp: 217.7 °C; **^{1}H NMR (400 MHz, $CDCl_3$):** δ (ppm) = 9.19 (d, *J* = 2.2 Hz, 1H), 9.06 (d, *J* = 2.0 Hz, 1H), 8.64 (pseudo-t, *J* = 2.1 Hz, 1H), 8.40 (d, *J* = 1.0 H, 1H), 8.32 (s, br, 1H), 8.08 (d, *J* = 8.6 Hz, 1H), 8.06–8.01 (m, 1H),7.65 (s, br, 1H), 7.30–7.12 (m, 5H), 2.93 (t, *J* = 8.1 Hz, 1H), 2.76 (t, *J* = 8.1 Hz, 1H); **^{13}C NMR, DEPT135, HSQC, HMBC (75.5 MHz, $CDCl_3$):** δ (ppm) = 166.4, 150.1, 147.7, 134.9, 133.7, 133.2, 133.1, 132.6, 129.7, 128.8, 128.3, 127.6, 126.7, 126.5, 124.8; **IR (ATR):** *v* (cm^{-1}) = 3385, 3190, 1686, 1397, 1144, 1129, 822, 775, 700, 479; **ESI-HRMS:** calcd. for $[C_{16}H_{12}N_2O+H]^+$ 248.0950, found 248.0943; **Comp. Purity (220 nm):** 100 %; **Elem. Anal.:** calcd. for $C_{16}H_{16}N_2O$: N 11.28, C 77.40, H 4.87, found: N 11.20, C 77.29, H 4.68.

Synthesis of **2d**

(*E*)-5-(4-Methoxystyryl)nicotinamide (**2d**)

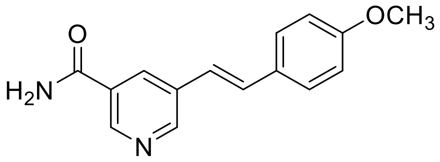

In an inert gas atmosphere 4-bromoanisole (468 mg, 2.50 mmol, 1.00 equiv) was mixed with **5a** (407 mg, 2.75 mmol, 1.0 equiv), $Pd_2(dba)_3$ (114 mg, 0.13 mmol, 0.05 equiv) and tris(*o*-tolyl)phosphine (76 mg, 0.25 mmol, 0.10 equiv) and suspended in anhydrous DMF (10 mL). After addition of NEt_3 (1.04 mL, 759 mg, 7.50 mmol, 3.00 equiv) the reaction mixture was stirred at 120 °C for 24 h. The mixture was filtrated through a pad of Celite® and taken up in EtOAc (70 mL). The organic phase was washed with water (3 × 30 mL) and aq. sat. NaCl-solution (30 mL), then dried over $MgSO_4$. After evaporation of the solvent under reduced pressure, the residue was recrystallized from cyclohexane/EtOAc. The product was obtained as colourless solid (110 mg, 0.43 mmol, 17%): R_f = 0.25 (DCM/MeOH 95:5); mp: 222.3 °C; **^{1}H NMR, H,H-COSY (400 MHz, DMSO-*d_6*):** 8.88 (d, *J* = 1.9 Hz, 1H), 8.84 (d, *J* = 1.9 Hz, 1H), 8.45–8.43 (m, 1H), 8.20 (s, br, 1H), 7.65 (s, br, 1H), 7.59 (d, 8.7 Hz, 1H), 7.42 (d, *J* = 16.5 Hz, 1H), 7.19 (d, *J* = 16.5 Hz, 1H), 6.99 (d, 8.7 Hz, 1H), 3.80 (s, 3H); **^{13}C NMR, DEPT135, HSQC, HMBC (75.5 MHz, DMSO-*d_6*):** 166.4, 159.4, 150.1, 146.8, 132.7, 131.0, 130.9, 129.6, 129.1, 128.1, 121.8, 114.2, 55.1; **IR (ATR):** *v* (cm^{-1}) = 3339, 2969, 1675, 1603, 1387, 1022, 953, 818, 696, 626; **ESI-HRMS:** calcd.

for $[C_{15}H_{14}N_2O_2+H]^+$ 254.1055, found 254.1056; **Comp. Purity (220 nm):** 100 %; **Elem. Anal.:** calcd. for $C_{15}H_{14}N_2O_2$: N 11.02, C 70.85, H 5.55, found: N 10.95, C 70.85, H 5.28.

Synthesis of **2e**

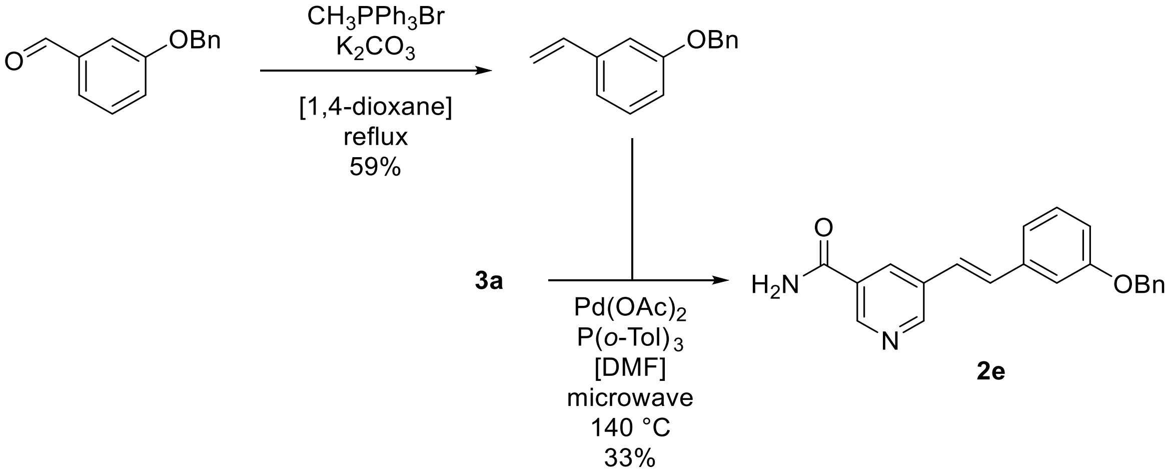

1-(Benzyloxy)-3-vinylbenzene

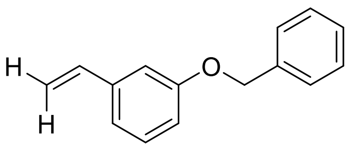

In an inert gas atmosphere 3-benzyloxybenzaldehyde (2.12 g, 10.00 mmol, 1.00 equiv) was added to a suspension of methyltriphenylphosphonium bromide (4.29 g, 12.00 mmol, 1.20 equiv) and K_2CO_3 (2.21 g, 16.00 mmol, 1.60 equiv) in anhydrous 1,4-dioxane (10 mL). The reaction mixture was stirred under reflux overnight, cooled, filtered and concentrated under reduced pressure. The residue was dissolved in hot *n*-hexane, filtered through a pad of silica gel and washed with *n*-hexane. After evaporation of the solvent under reduced pressure the product was obtained as a colourless oil (1.24 g, 5.91 mmol, 59%): R_f = 0.23 (*n*-hexane); **^{1}H NMR, H,H-COSY (400 MHz, DMSO-*d₆*):** 7.49–7.43 (m, 2H), 7.43–7.36 (m, 2H), 7.35–7.29 (m, 1H), 7.26 (pseudo-t, *J* = 7.9 Hz, 1H), 7.14 (m, 1H), 7.05 (d, *J* = 7.6 Hz, 1H), 6.93 (ddd, *J* = 8.2, 2.5, 0.7 Hz, 1H), 6.70 (dd, *J* = 17.7, 10.9 Hz, 1H), 5.84 (dd, *J* = 17.6, 0.9 Hz, 1H), 5.26 (dd, *J* = 10.9, 0.9 Hz), 5.12 (s, 2H); **^{13}C NMR, DEPT135, HSQC, HMBC (75.5 MHz, DMSO-d_6):** 158.6, 138.6, 137.1, 136.5, 129.6, 128.4, 127.8, 127.7, 118.9, 114.6, 114.4, 112.2, 69.1; **IR (ATR):** v (cm^{-1}) = 3031, 2866, 1597, 1574, 1485, 1441, 1154, 1024, 989, 781.

(*E*)-5-[3-(Benzyloxy)styryl]nicotinamide (**2e**)

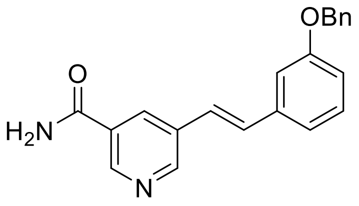

Synthesis was conducted according to the procedure of **2b** using **3a** (603 mg, 3.00 mmol, 1.00 equiv), 1-(benzyloxy)-3-vinylbenzene (946 mg, 4.50 mmol, 1.50 equiv), tris(*o*-tolyl)phosphine (91 mg, 0.30 mmol, 0.10 equiv), $Pd(OAc)_2$ (34 mg, 0.15 mmol, 0.05 equiv) and NEt_3 (1.25 mL, 913 mg, 9.00 mmol, 3.00 equiv) in anhydrous DMF (4 mL). The reaction was conducted at 140 °C for

1.5 h in a microwave reactor. The raw product was recrystallized from ACN yielding a colourless solid (328 mg, 0.99 mmol, 33%): R_f = 0.51 (EtOAc/MeOH 4:1); mp: 190.4 °C; **^{1}H NMR, H,H-COSY (400 MHz, DMSO-*d₆*):** δ (ppm) = 8.92 (s, 1H), 8.88 (s, 1H), 8.48 (s, 1H), 8.22 (s, br, 1H), 7.67 (s, br, 1H), 7.49 (d, *J* = 7.6 Hz, 2H), 7.46–7.38 (m, 4H), 7.37–7.27 (m, 3H), 7.23 (d, *J* = 7.4 Hz, 1H), 6.98 (d, *J* = 7.9 Hz, 1H), 5.17 (s, 2H); **^{13}C NMR, DEPT135, HSQC, HMBC (75.5 MHz, DMSO-d_6):** δ (ppm) = 166.3, 158.7, 150.4, 147.3, 137.9, 137.0, 132.3, 131.4, 131.1, 129.8, 129.7, 128.4, 127.8, 127.7, 124.6, 119.6, 114.8, 112.5, 69.2; **IR (ATR):** *v* (cm^{-1}) = 3382, 3190, 1652, 1619, 1585, 1292, 1249, 1012, 965, 696; **ESI-HRMS:** calcd. for $[C_{21}H_{18}N_2O_2+H]^+$ 330.1368, found 330.1365; **Comp. Purity (220 nm):** 99.9 %; **Elem. Anal.:** calcd. for $C_{21}H_{18}N_2O_2$: N 8.48, C 76.34, H 5.49, found: N 8.35, C 75.89, H 5.19.

Synthesis of **2f**

(*E*)-5-(4-Methoxy-2,6-dimethylstyryl)nicotinamide (**2f**)

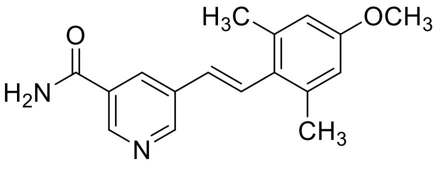

Synthesis was conducted according to the procedure of **2d** using **5a** (593 mg, 2.76 mmol, 1.00 equiv), 2-bromo-5-methoxy-1,3-dimethylbenzene (532 mg, 3.59 mmol, 1.30 equiv), $Pd_2(dba)_3$ (128 mg, 0.14 mmol, 0.05 equiv), tris(*o*-tolyl)phosphine (85 mg, 0.28 mmol, 0.10 equiv) and NEt_3 (1.15 mL, 838 mg, 8.28 mmol, 3.00 equiv) in anhydrous DMF (10 mL). The reaction was conducted at 120 °C for 3 h. The crude product was purified by silica gel column chromatography (EtOAc/MeOH 95:5), which yielded a colourless solid (88 mg, 0.31 mmol, 11 %): R_f = 0.39 (EtOAc/MeOH 95:5); mp: 188.3 °C; **^{1}H NMR, H,H-COSY (400 MHz, DMSO-d_6):** 8.90 (d, *J* = 2.0 Hz, 1H), 8.86 (d, *J* = 2.0 Hz, 1H) , 8.43 (pseudo-t, *J* = 2.0 Hz, 1H), 8.21 (s, br, 1H), 7.65 (s, br, 1H), 7.39 (d, *J* = 16.8 Hz, 1H), 6.72 (d, *J* = 17.4 Hz, 1H), 6.69 (s, 2H), 3.75 (s, 3H), 2.35 (s, 6H); **^{13}C NMR, DEPT135, HSQC, HMBC (75.5 MHz, DMSO-d_6):** 166.4, 157.8, 150.0, 147.1, 137.4, 132.7, 131.1, 129.6, 129.2, 128.5, 128.3, 113.4, 54.8, 21.1; **IR (ATR):** *v* (cm^{-1}) = 3171, 2958, 1670, 1598, 1383, 1308, 1299, 1145, 1064, 691; **ESI-HRMS:** calcd. for $[C_{17}H_{18}N_2O_2+H]^+$ 282.1368, found 282.1361; **Comp. Purity (220 nm):** 100%; **Elem. Anal.:** calcd. for $C_{17}H_{18}N_2O_2$: N 9.92, C 72.32, H 6.41, found: N 9.61, C 71.77, H 6.61.

Synthesis of **2h**

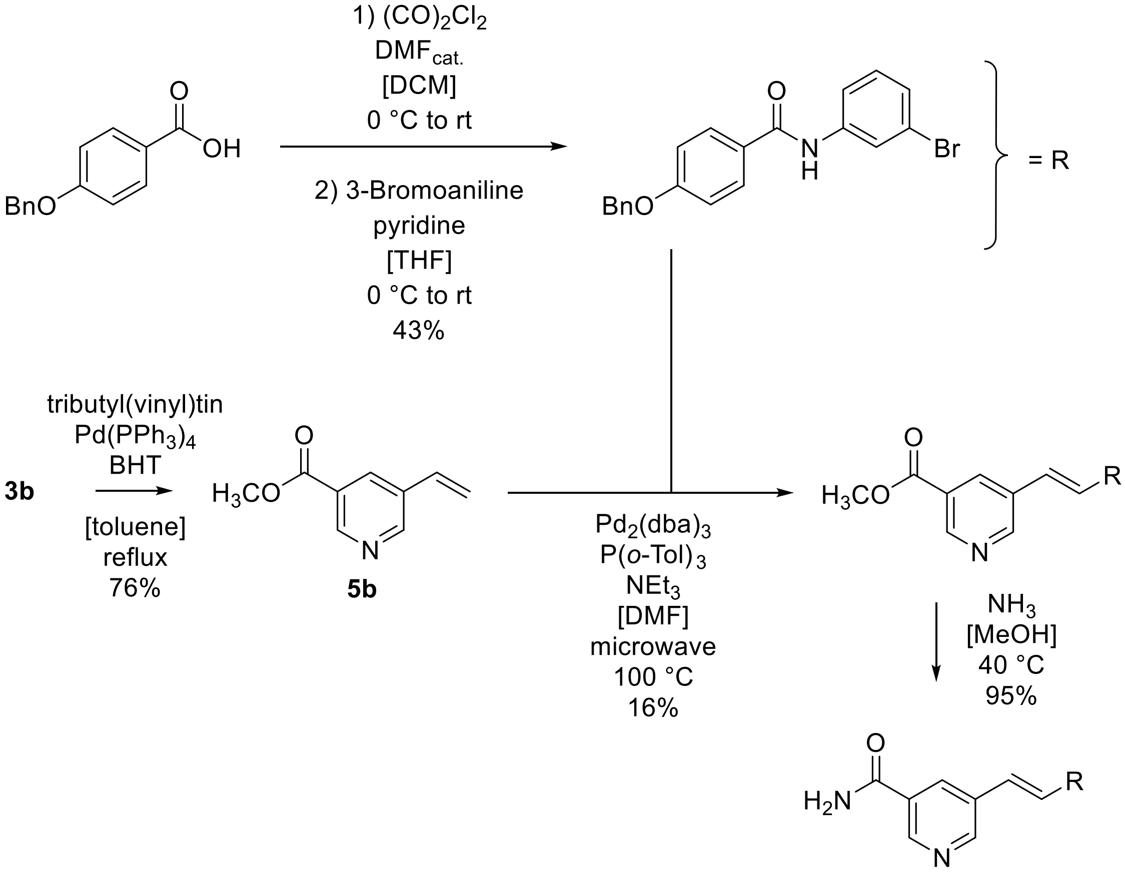

2h

Methyl 5-vinylnicotinate (**5b**)

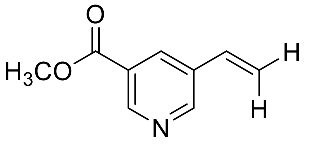

In an inert gas atmosphere **3b** (262 mg, 1.21 mmol, 1.00 equiv) was dissolved in anhydrous toluene (10 mL) and tributyl(vinyl)tin (577 mg, 1.82 mmol, 1.50 equiv), $Pd(PPh_3)_4$ (75 mg, 0.06 mmol, 0.05 equiv) and a few crystals of BHT were added. The mixture was stirred under reflux for 3 h, cooled to room temperature and filtrated through a pad of Celite®. The filtrate was taken up in EtOAc (30 mL) and the organic phase washed with water (3 × 10 mL) and aq. sat. NaCl-solution (30 mL), then dried over $MgSO_4$. After evaporation of the solvent under reduced pressure, the residue was purified by silica gel column chromatography (*n*-hexane/EtOAc 1:1). The product was obtained as colourless oil that crystallized on storage at -20 °C (150 mg, 0.92 mmol, 76%): R_f = 0.58 (*n*-hexane/EtOAc 1:1); mp: 30.5 °C; **^{1}H NMR (400 MHz, $CDCl_3$):** 9.10 (d, *J* = 1.8 Hz, 1H), 8.79 (d, *J* = 2.1 Hz, 1H), 8.39 (pseudo-t, *J* = 2.0 Hz, 1H), 6.76 (dd, *J* = 17.7, 11.0 Hz, 1H), 5.96 (d, *J* = 17.6 Hz, 1H), 5.52 (d, *J* = 11.0 Hz, 1H), 3.98 (s, 3H); **^{13}C NMR, DEPT135, HSQC, HMBC (75.5 MHz, $CDCl_3$):** 165.5, 150.8, 148.9, 134.6, 133.6, 132.3, 126.5, 118.5, 52.8; **IR (ATR):** ν (cm^{-1}) = 3006, 2955, 1718, 1455, 1434, 1294, 1205, 1112, 941, 768.

4-(Benzyloxy)-*N*-(3-bromophenyl)benzamide

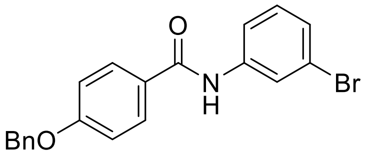

In an inert gas atmosphere a solution of 4-benzyloxy benzoic acid (1.14 g, 5.00 mmol, 1.00 equiv) in anhydrous DCM (30 mL) was cooled to 0 °C and treated with anhydrous DMF (75 μL) and oxalyl chloride (0.64 mL, 947 mg, 5.50 mmol 1.10 equiv). After complete addition, the solution was stirred at room temperature until no further formation of gas was observed. Under reduced pressure the solution was concentrated to a volume of 10 mL and slowly added to a solution of 3-bromoaniline (0.60 mL, 946 mg, 5.50 mmol, 1.10 equiv) and pyridine (1.21 mL, 1.19 g, 15.00 mmol, 3.00 equiv) in anhydrous THF under inert gas at 0 °C. After complete addition the reaction mixture was stirred at room temperature for 3 h, then freed from solvent under reduced pressure. Purification of the residue by silica gel column chromatography (cyclohexane/EtOAc 2:1) yielded the product as colourless solid (816 mg, 2.13 mmol, 43%): R_f = 0.53 (cyclohexane/EtOAc 4:1); mp: 167.1 °C; **^{1}H NMR, H,H-COSY (400 MHz, DMSO-*d$_6$*):** δ (ppm) = 10.22 (s, 1H), 8.11 (t, *J* = 1.9 Hz, 1H), 7.98–7.92 (m, 2H), 7.78–7.73 (m, 1H), 7.51–7.45 (m, 2H), 7.44–7.38 (m, 2H), 7.37–7.32 (m, 1H), 7.30 (d, *J* = 7.8 Hz, 1H), 7.27 (dt, *J* = 8.0, 1.5 Hz, 1H), 7.18–7.12 (m, 2H), 5.21 (s, 2H); **^{13}C NMR, DEPT135, HSQC, HMBC (75.5 MHz, DMSO-*d$_6$*):** δ (ppm) =165.1, 161.1, 141.0, 136.6, 130.6, 129.7, 128.5, 128.0, 127.8, 126.7, 125.9, 122.4, 121.4, 118.9, 114.5, 69.4; **IR (ATR):** *v* (cm^{-1}) = 3294,1644, 1582, 1499, 1413, 1284, 1229, 1069, 887, 749.

Methyl (*E*)-5-{3-[4-(benzyloxy)benzamido]styryl}nicotinate

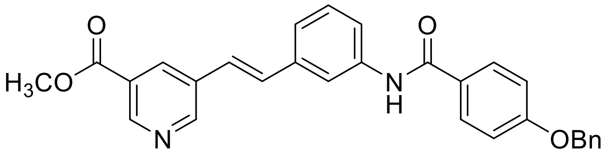

In a microwave reaction vessel methyl 5-vinylnicotinate (130 mg, 0.80 mmol, 1.80 equiv) was mixed with 4(benzyloxy)-*N*-3-bromophenyl)benzamide (170 mg, 0.44 mmol, 1.00 equiv), tris(*o*-tolyl)phosphine (27 mg, 0.09 mmol, 0.20 equiv), $Pd_2(dba)_3$ (40 mg, 0.04 mmol, 0.10 equiv) and NEt_3 (183 μL, 1.32 mmol, 3.00 equiv) and suspended in anhydrous DMF (3 mL). The reaction was conducted at 100 °C for 1.5 h. After cooling to room temperature the mixture was taken up in EtOAc (50 mL) and filtered through a pad of Celite®. The filtrate was washed with water (3 × 30 mL) and sat. aq. NaCl-solution (30 mL), then dried over $MgSO_4$. The solvent was evaporated under reduced pressure and the residue purified by silica gel column chromatography (*n*-hexane/EtOAc 2:1). The product was obtained as a colourless solid (32 mg, 0.07 mmol, 16%): R_f = 0.23 (*n*-hexane/EtOAc 2:1); mp: 197.9 °C; **^{1}H NMR, H,H-COSY (400 MHz, DMSO-*d$_6$*):** δ (ppm) = 10.17 (s, 1H), 9.06 (d, *J* = 2.0 Hz, 1H), 8.96 (d, *J* = 1.9 Hz, 1H), 8.52 (pseudo-t, *J* = 1.9 Hz, 1H), 8.12 (s, 1H), 8.00 (d, *J* = 8.8 Hz, 2H), 7.68 (dt, *J* = 6.8, 1.9 Hz, 1H), 7.55 (d, *J* = 16.5 Hz, 1H, 1H), 7.49 (d, *J* = 7.1 Hz, 2H), 7.45–7.35 (m, 5H), 7.32 (d, 1H, J = 16.6 Hz, 1H), 7.17 (d, *J* = 8.9 Hz, 2H), 5.22 (s, 2H), 3.93 (s, 3H); **^{13}C NMR,**

DEPT135, HSQC, HMBC (75.5 MHz, DMSO-*d*6): δ (ppm) =165.2, 164.9, 161.0, 151.9, 148.4, 139.7, 136.8, 136.7, 133.3, 132.9, 132.0, 129.6, 128.6, 128.5, 128.0, 127.8, 127.1, 125.7, 123.9, 122.1, 121.0, 118.8, 114.5, 69.4, 52.5.

(*E*)-5-{3-[4-(Benzyloxy)benzamido]styryl}nicotinamide (**2h**)

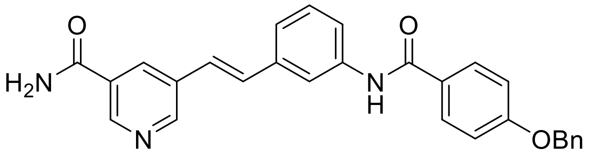

Synthesis was conducted following the general procedure of nicotinamides from methyl nicotinates, using methyl (*E*)-5-{3-[4-(benzyloxy)benzamido]styryl}nicotinate (30 mg, 0.065 mmol, 1.00 equiv). The product was obtained as colourless solid (28 mg, 0.062 mmol, 95%): R_f = 0.50 (EtOAc/MeOH 95:5); mp: 255.0 °C; **^{1}H NMR (400 MHz, DMSO-*d*6):** δ (ppm) = 10.15 (s, br), 8.90 (pseudo-d, *J* = 1.8 Hz, 2H), (pseudo-t, *J* = 2.0 Hz, 1H), 8.20 (s, br, 1H), 8.11 (s, 1H), 8.02–7.95 (m, 2H), 7.71–7.62 (m, 2H), 7.52–7.45 (m, 3H), 7.45–7.38 (m, 4H), 7.38–7.33 (m, 1H), 7.29 (d, *J* = 16.5 Hz, 1H), 7.19–7.14 (m, 2H), 5.22 (s, 2H); **^{13}C NMR, DEPT135, HSQC, HMBC (75.5 MHz, DMSO-*d*6):** δ (ppm) = 166.4, 165.0, 161.0, 150.5, 147.5, 139.8, 136.8, 136.7, 132.3, 131.4, 129.7, 129.6, 129.0, 128.5, 128.0, 127.8, 127.1, 124.4, 122.0, 120.4, 118.6, 114.5, 69.4; **IR (ATR):** *v* (cm^{-1}) = 3381, 3300, 3189, 1651, 1640, 1624, 1604, 1226, 965, 698; **ESI-HRMS:** calcd. for $[C_{28}H_{23}N_3O_3+H]^+$ 449.1739, found 449.1744; **Comp. Purity (220 nm):** 100 %; **Elem.Anal.:** calcd. for $C_{28}H_{23}N_3O_3$: N 9.35, C 74.82, H 5.16, found: N 9.04, C 73.95, H 4.87.

Synthesis of **2c** and **2g**

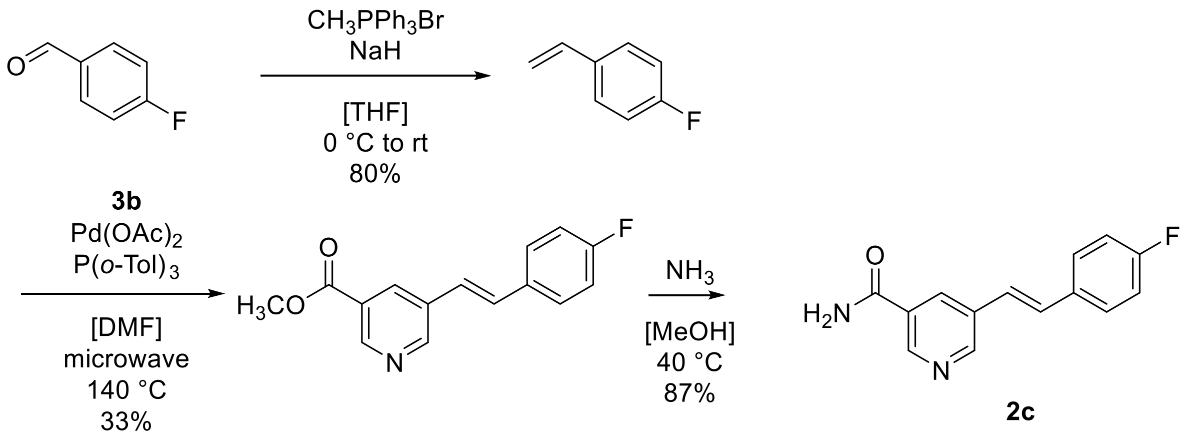

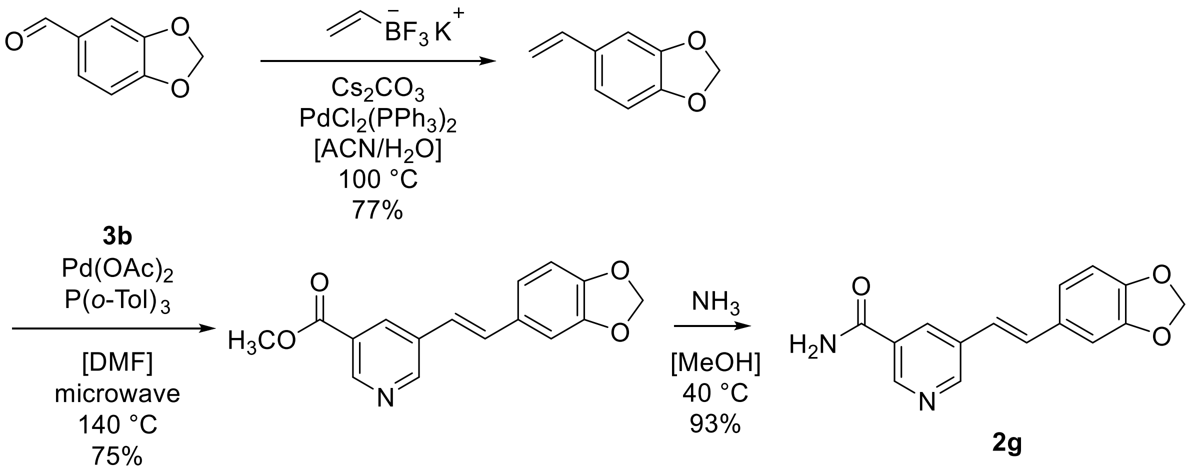

1-Fluoro-4-vinylbenzene

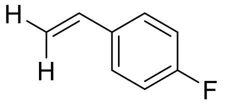

In an inert gas atmosphere 4-fluorobenzaldehyde (1.24 g, 10.00 mmol, 1.00 equiv) and methyltriphenylphosphonium bromide (4.29 g, 12.00 mmol, 1.20 equiv) were dissolved in anhydrous THF (50 mL). At 0 °C sodium hydride (60 % dispersion in mineral oil, 1.80 g, 45.00 mmol, 4.50 equiv) was added in small portions and the mixture was stirred over night at room temperature. The reaction mixture was taken up in EtOAc (100 mL) and washed with aq. sat. NaCl-solution (3 × 20 mL). It was dried over $MgSO_4$ and freed from solvent under reduced pressure. The residue was purified by silica gel column chromatography (*n*-hexane), which yielded the product as a colourless oil (980 mg, 8.02 mmol, 80%): R_f = 0.74 (*n*-hexane); **^{1}H NMR, H,H-COSY (400 MHz, $CDCl_3$):** 7.40–7.34 (m, 1H), 7.06–6.96 (m, 2H), 6.68 (dd, *J* = 17.6, 10.9 Hz, 1H), 5.66 (d, *J* = 17.6 Hz, 1H), 5.21 (d, = 10.9 Hz, 1H); **^{13}C NMR, DEPT135, HSQC, HMBC (75.5 MHz, $CDCl_3$):** 162.5 (d, *J* = 246.8 Hz), 135.7, 133.8 (d, *J* = 3.3 Hz), 127.7 (d, *J* = 8.0 Hz), 115.4 (d, *J* = 21.6 Hz), 113.5 (d, *J* = 2.2 Hz); **IR (ATR):** *v* (cm^{-1}) = 2953, 2921, 2852, 1744, 1713, 1458, 1377, 1260, 1168, 1111.

5-Vinyl-1,3-benzodioxole

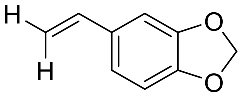

Synthesis was conducted according to the procedure of **5a** using 5-bromo-1,3-benzodioxole (4.02 g, 20.00 mmol, 1.00 equiv), potassium vinyltrifluoroborate (3.48 g, 26.00 mmol, 1.30 equiv), Cs_2CO_3 (19.55 g, 60.00 mmol, 3.00 equiv) and $PdCl_2(PPh_3)_2$ (1.40 g, 2.00 mmol, 0.10 equiv). The reaction mixture was stirred at 100 °C for 24 h. The crude product was purified by silica gel column chromatography (*n*-hexane), which yielded a colourless oil (2.29 g, 15.48 mmol, 77%): R_f = 0.37 (*n*-hexane); **^{1}H NMR, H,H-COSY (400 MHz, DMSO-*d*$_6$):** 7.14 (d, *J* = 1.5 Hz, 1H), 6.90 (dd, *J* = 8.1, 1.5 Hz, 1H), 6.87 (dd, *J* = 8.0, 0.5 Hz, 1H), 6.64 (dd, *J* = 17.6, 10.9 Hz, 1H), 6.02 (s, 2H), 5.68 (dd, *J* = 17.6, 0.9 Hz, 1H), 5.12 (dd, *J* = 10.9, 0.9 Hz, 1H); **^{13}C NMR, DEPT135, HSQC, HMBC (75.5 MHz, DMSO-*d*$_6$):** 147.7, 147.0, 136.2, 131.5, 121.0, 112.3, 108.1, 105.1, 101.0; **IR (ATR):** *v* (cm^{-1}) = 3085, 2889, 1604, 1502, 1487, 1442, 1348, 1243, 1039, 810.

Methyl (*E*)-5-[2-(benzo-1,3-dioxol-5-yl)vinyl]nicotinate

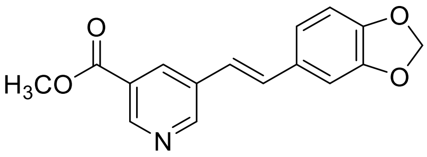

In a microwave reaction vessel **3b** (648 mg, 3.00 mmol, 1.00 equiv) was mixed with 5-vinyl-1,3-benzodioxole (667 mg, 4.50 mmol, 1.50 equiv), tris(*o*-tolyl)phosphine (183 mg, 0.60 mmol, 0.20 equiv), $Pd(OAc)_2$ (67 mg, 0.30 mmol, 0.10 equiv) and NEt_3 (1.25 mL, 9.00 mmol, 3.00 equiv) and suspended in anhydrous DMF (4 mL). The reaction was conducted at 140 °C for 1.5 h. After cooling to room temperature the mixture was taken up in EtOAc (50 mL) and filtered through a pad of Celite®. The filtrate was washed with water (3 × 30 mL) and sat. aq. NaCl-solution (30 mL), then dried over $MgSO_4$. The solvent was evaporated under reduced pressure and the residue purified by silica gel column chromatography (*n*-hexane/EtOAc 2:1). The product was obtained as a colourless solid (636 mg, 2.25 mmol, 75 %): R_f = 0.34 (*n*-hexane/EtOAc 2:1); mp: 120.1 °C; **^{1}H NMR, H,H-COSY (400 MHz, DMSO-*d*$_6$):** 8.96 (d, *J* = 2.0 Hz, 1H), 8.91 (d, *J* = 1.8 Hz, 1H), 8.41 (d, *J* = 1.8 Hz, 1H), 7.44 (d, *J* = 16.5 Hz, 1H), 7.32 (d, *J* = 1.4 Hz, 1H), 7.22 (d, *J* = 16.5 Hz, 1H), 7.10 (dd, *J* = 8.1, 1.4 Hz, 1H), 6.95 (d, *J* = 8.0 Hz, 1H), 6.07 (s, 2H), 3.92 (s, 3H); **^{13}C NMR, DEPT135, HSQC, HMBC (75.5 MHz, DMSO-*d*$_6$):** 165.2, 151.5, 148.0, 147.9, 147.5, 133.1, 132.8, 131.5, 131.0, 122.3, 121.9, 108.4, 105.5, 101.2; **IR (ATR):** *v* (cm^{-1}) = 2953, 2901, 1716, 1491, 1444, 1430, 1307, 1036, 930, 611.

(*E*)-5-[2-(Benzo[*d*][1,3]dioxol-5-yl)vinyl]nicotinamide (**2g**)

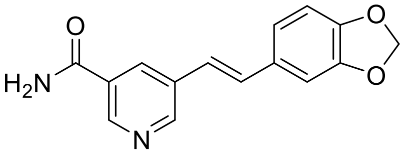

Synthesis was conducted following the general procedure of nicotinamides from methyl nicotinates, using methyl (*E*)-5-[2-(benzo-1,3-dioxol-5-yl)vinyl]nicotinate (200 mg, 0.71 mmol, 1.00 equiv). The product was obtained as colourless solid (178 mg, 0.66 mmol, 93 %): R_f = 0.60 (EtOAc/MeOH 95:5); mp: 249.7 °C; **^{1}H NMR, H,H-COSY**

(400 MHz, DMSO-d_6): δ (ppm) = 8.89 (d, *J* = 1.9 Hz, 1H), 8.83 (d, *J* = 1.9 Hz, 1H), 8.43 (pseudo-t, *J* = 1.8 Hz, 1H), 8.22 (s, br, 1H), 7.66 (s, br, 1H), 7.40 (d, *J* = 16.5 Hz, 1H), 7.33 (d, *J* = 1.3 Hz, 1H), 7.21 (d, *J* = 16.5 Hz, 1H), 7.09 (dd, *J* = 8.1, 1.3 Hz, 1H), 6.96 (d, *J* = 8.0 Hz, 1H), 6.07 (s, 2H); **^{13}C NMR, DEPT135, HSQC, HMBC (75.5 MHz, DMSO-d_6):** δ (ppm) = 166.4, 150.1, 147.9, 147.4, 147.0, 132.6, 131.1, 131.0, 131.0, 129.6, 122.4, 122.2, 108.4, 105.4, 101.2; **IR (ATR):** *v* (cm^{-1}) = 3306, 3111, 1685, 1627, 1488, 1436, 1025, 917, 794, 697; **ESI-HRMS:** calcd. for $[C_{15}H_{12}N_2O_3+H]^+$ 268.0848, found 268.0850; **Comp. Purity (220 nm):** 100 %; **Elem.Anal.:** calcd. for $C_{15}H_{12}N_2O_3$: N 10.44, C 67.16, H 4.51, found: N 10.40, C 67.22, H 4.28.

Synthesis of **8a**

Benzo[*h*]quinoline-3-carboxamide (**8a**)

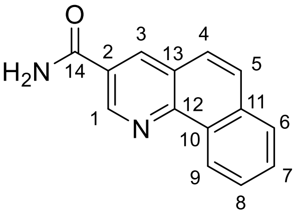

To a solution of **2b** (673 mg, 3.00 mmol, 1.00 equiv) in methanol (350 mL) was added a solution of iodine (38 mg, 0.15 mmol, 0.05 equiv) in methanol (50 mL). A slow stream of compressed air was bubbled through the solution, while it was irradiated with UV-light of 254 nm (Figure S2). After complete consumption of **2b** (24 hours), the solvent was removed under reduced pressure. Purification of the residue by silica gel column chromatography (*n*-hexane/THF 1:1) yielded **8a** as colourless solid (80 mg, 0.36 mmol, 12 %): R_f = 0.32 (*n*-hexane/THF 1:1); mp: 276.8 °C (Dekomp.); **^{1}H NMR, H,H-COSY (400 MHz, DMSO-d_6):** δ (ppm) = 9.48 (d, *J* = 2.2 Hz, 1H, 1-H), 9.26–9.19 (m, 1H, 9-H), 8.89 (d, *J* = 2.1 Hz, 1H, 3-H), 8.36 (s, br, 1H, *N*-H), 8.12–8.07 (m, 1H, 6-H), 8.03 (d, *J* = 8.9 Hz, 1H, 5-H), 7.95 (d, *J* = 8.9 Hz, 1H, 4-H), 7.85–7.78 (m, 2H, 7-H, 8-H), 7.74 (s, br, 1H, *N*-H); **^{13}C NMR, DEPT135, HSQC, HMBC (75.5 MHz, DMSO-d_6):** δ (ppm) = 166.4 (14), 147.8 (1), 146.7 (12), 135.5 (3), 133.8 (13), 130.3 (10), 129.0 (7), 128.1 (5/6), 128.0 (5/6), 127.8 (2), 127.3 (8), 125.8 (4), 124.9 (11), 124.1 (9); **IR (ATR):** *v* (cm^{-1}) = 3336, 3136, 1686, 1482, 1395, 1295, 801, 691, 539, 489; **ESI-HRMS:** calcd. for $[C_{14}H_{10}N_2O+H]^+$ 222.0793, found 222.0796; **Comp. Purity (220 nm):** 100 %.

NMR spectra

2-Bromo-3,4-dihydro-2*H*-naphthalen-1-one

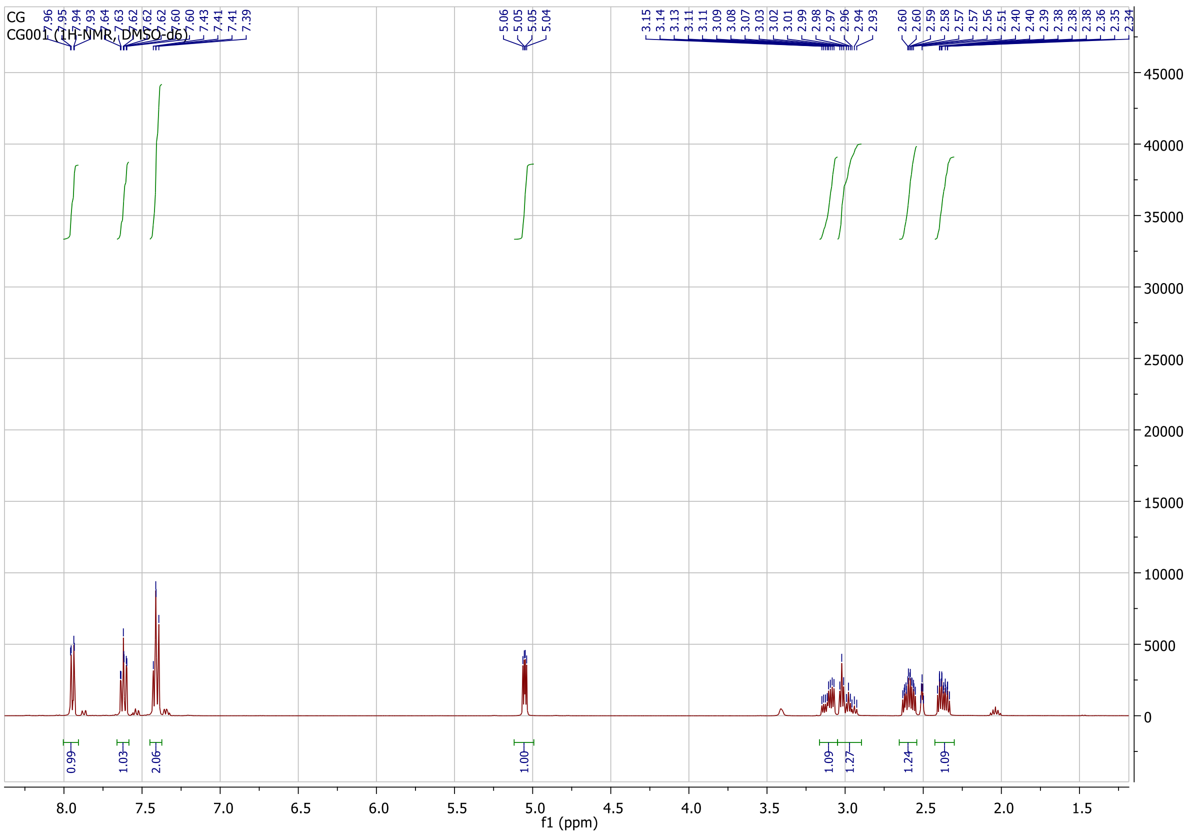

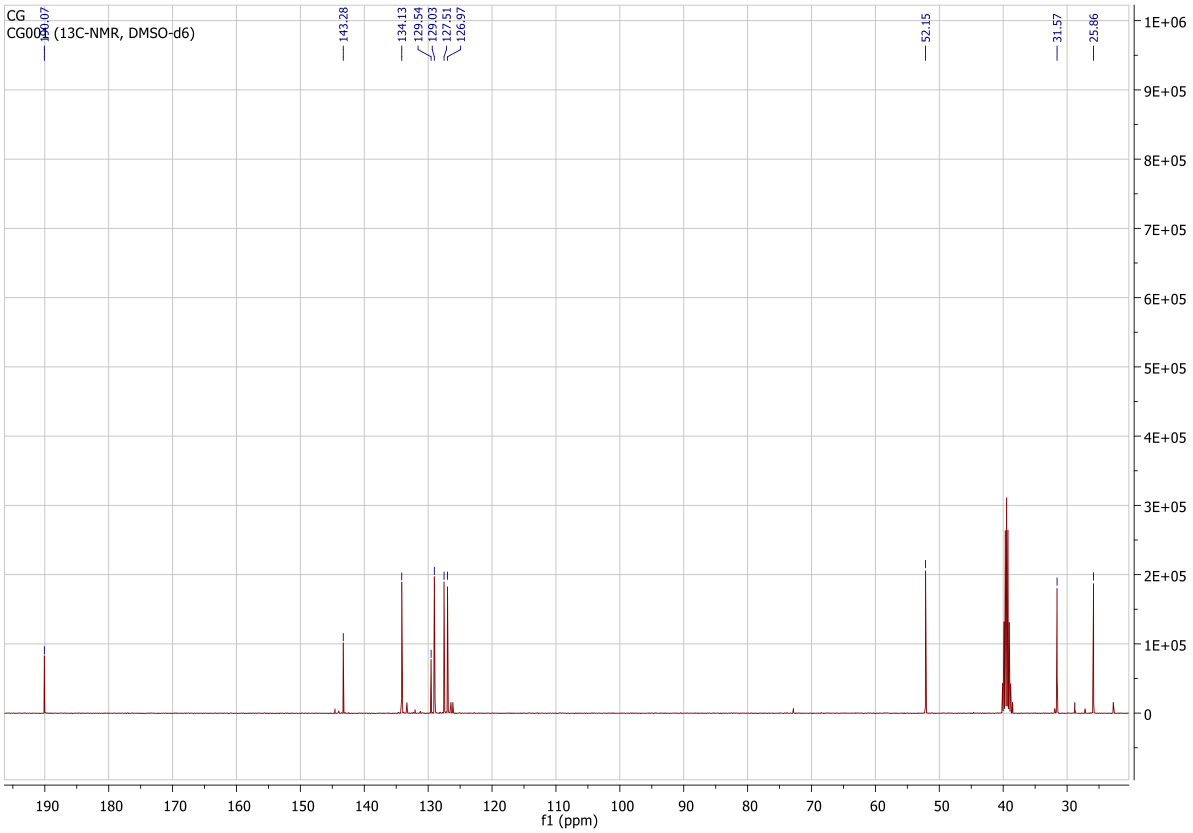

2-Bromo-1,2,3,4-tetrahydronaphthalen-1-ol

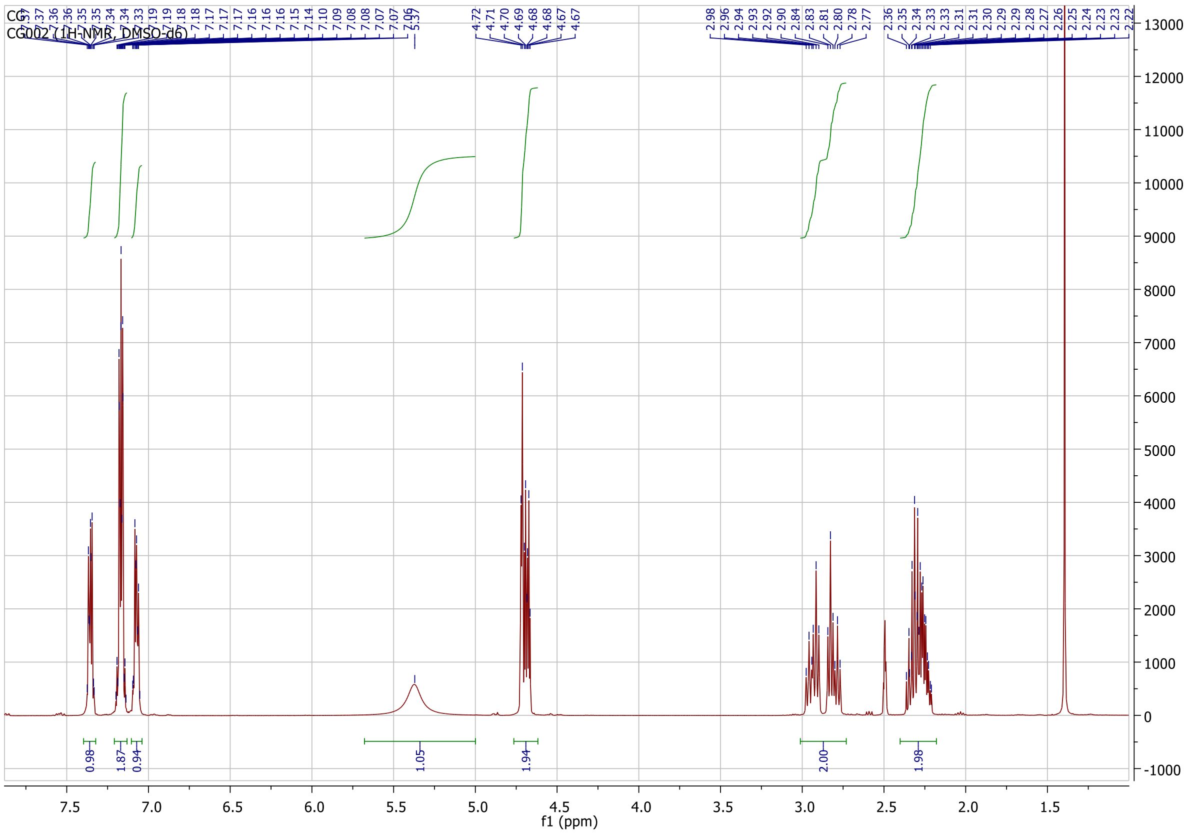

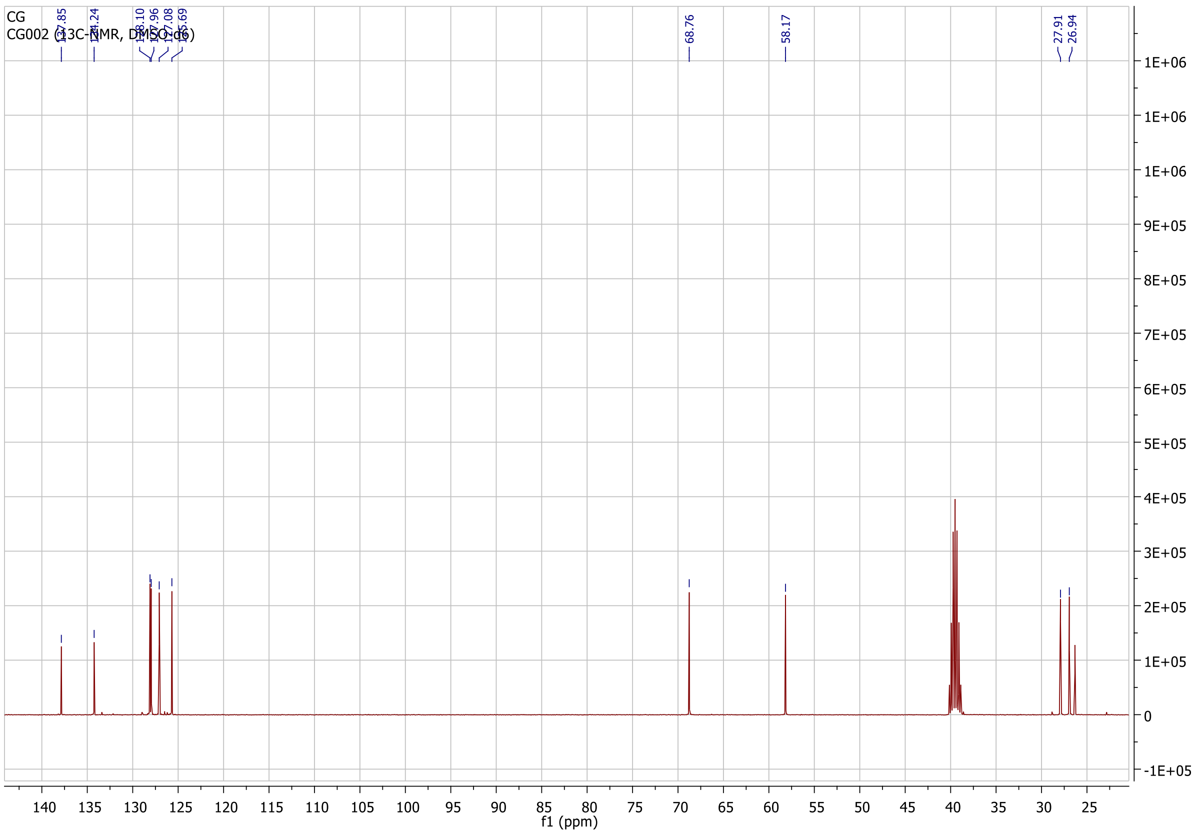

3-Bromo-1,2-dihydronaphthalene

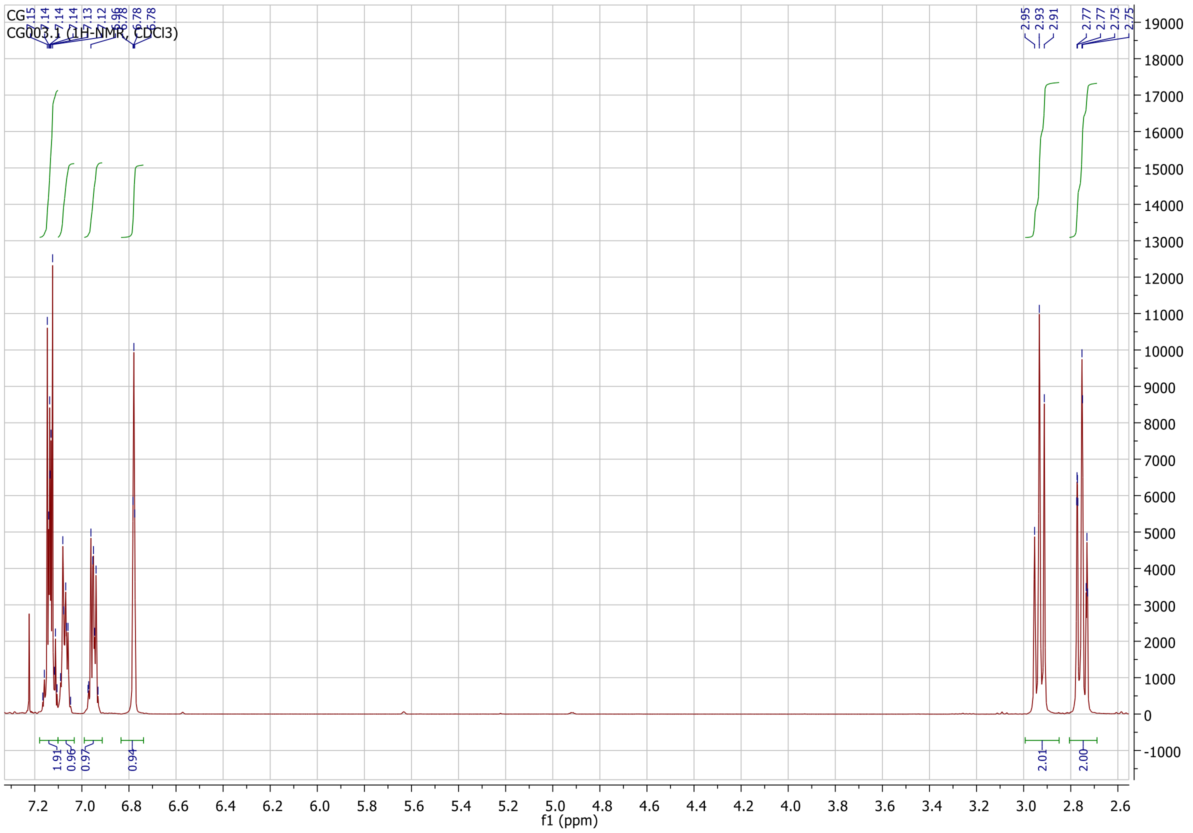

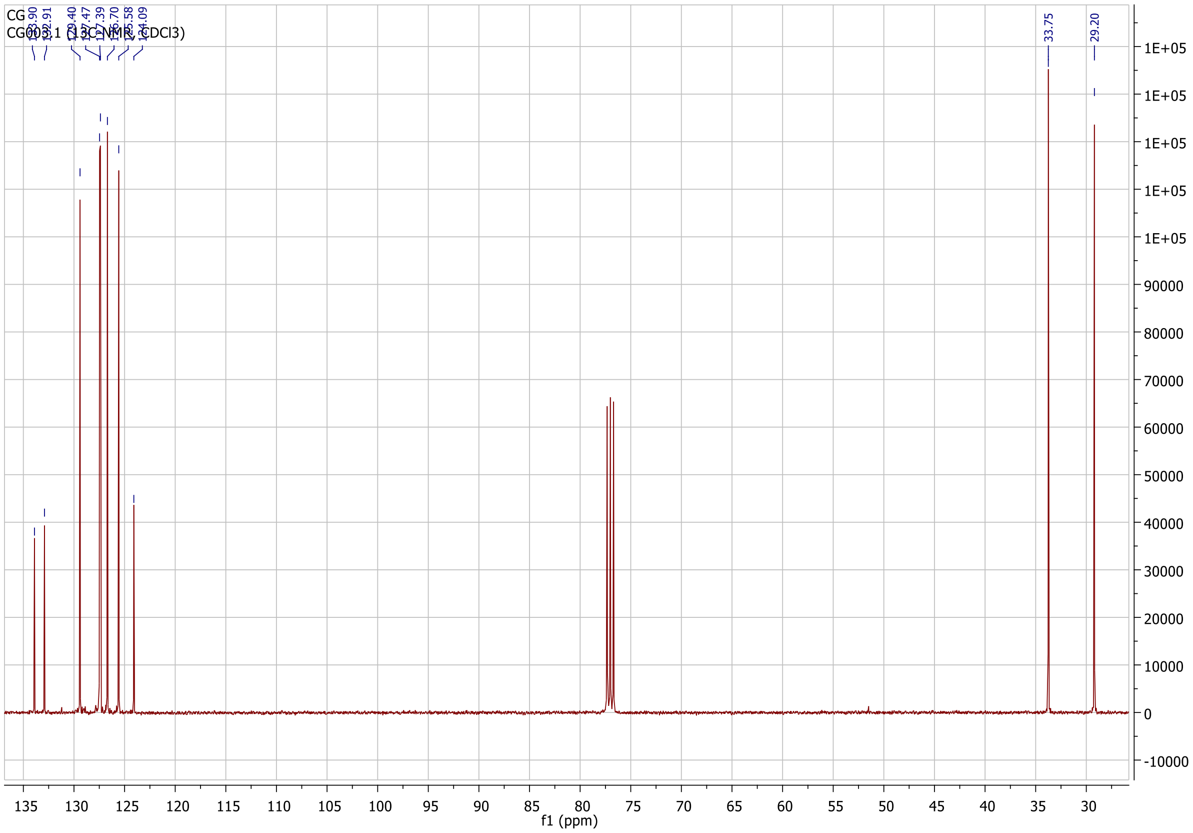

(3,4-Dihydronaphthalen-2-yl)boronic acid

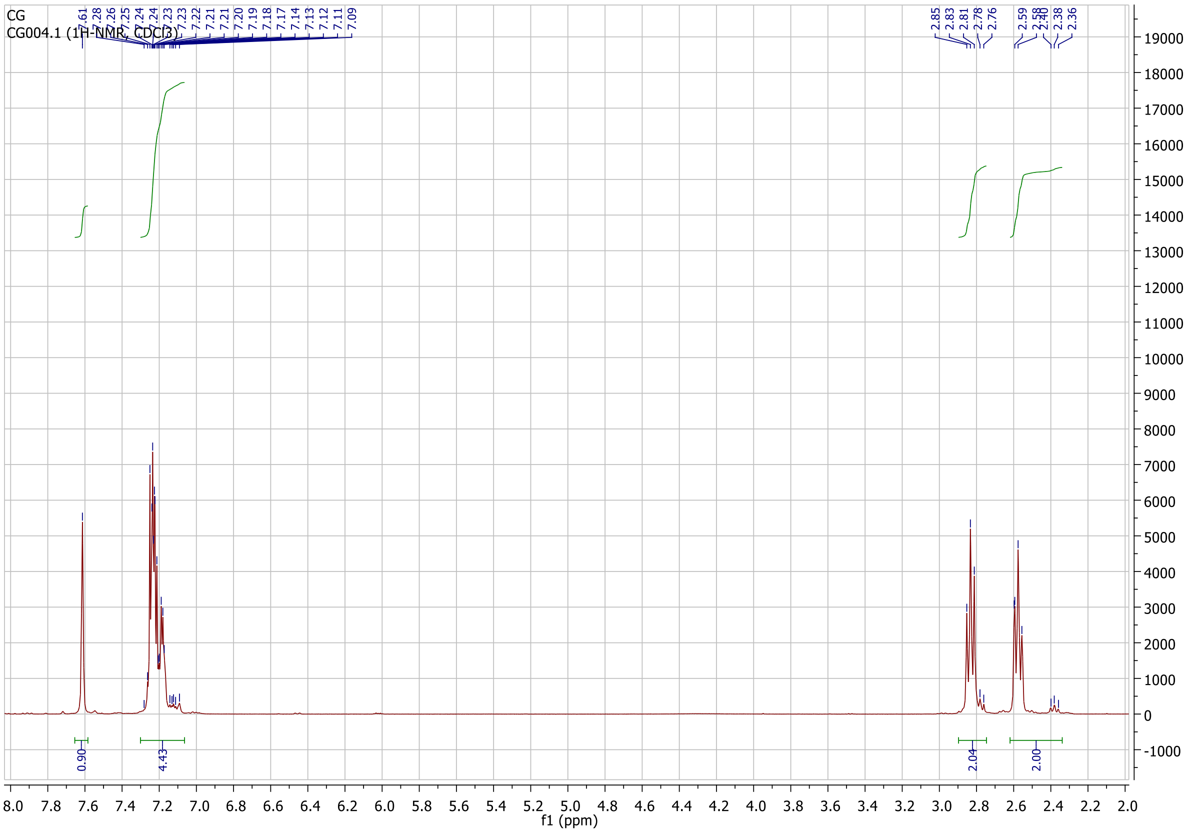

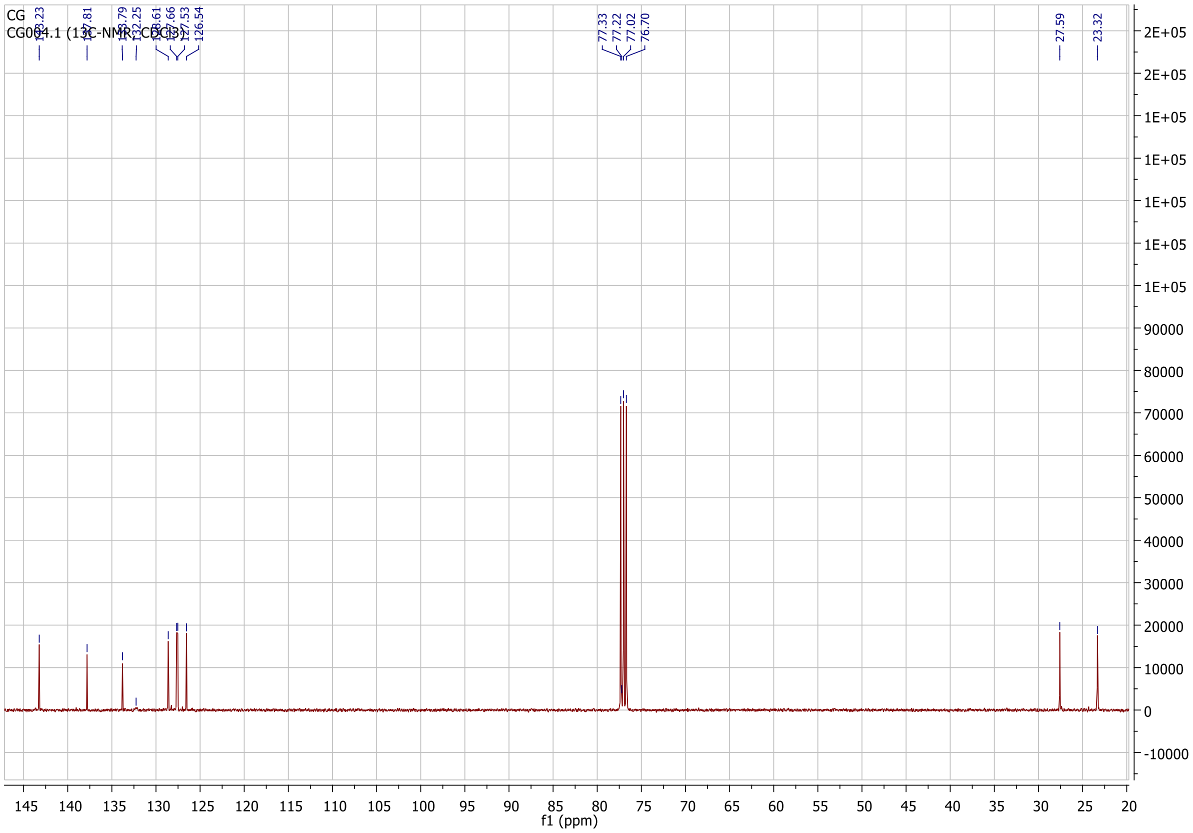

5-(3,4-Dihydronaphthalen-2-yl)nicotinamide (**4a**)

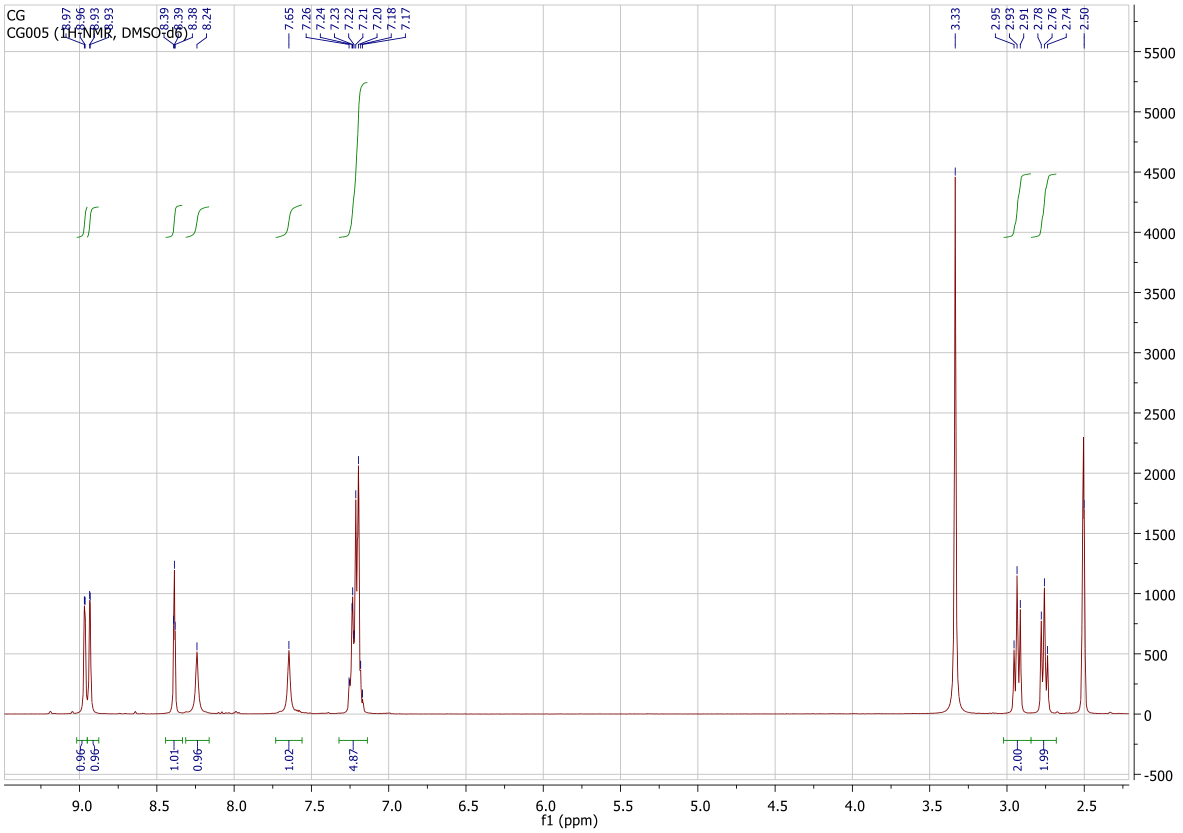

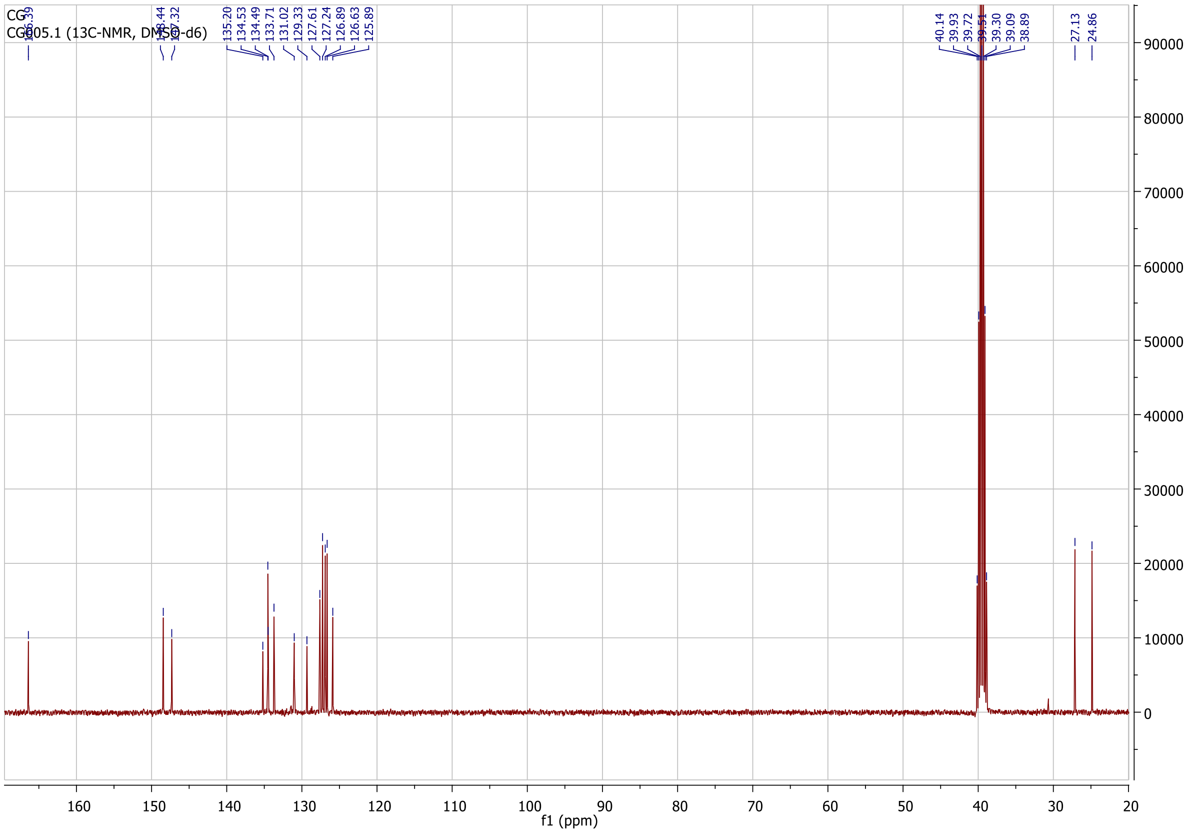

5-(Naphthalen-2-yl)nicotinamide (**4b**)

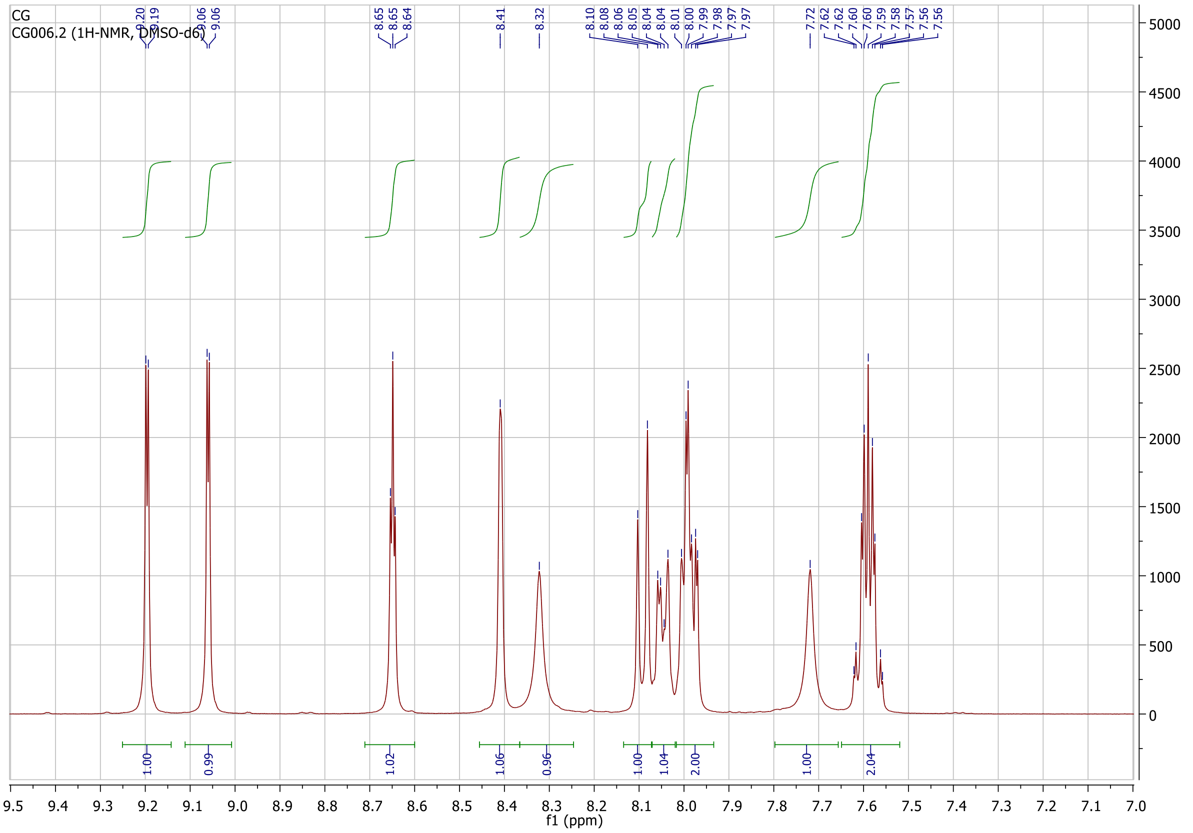

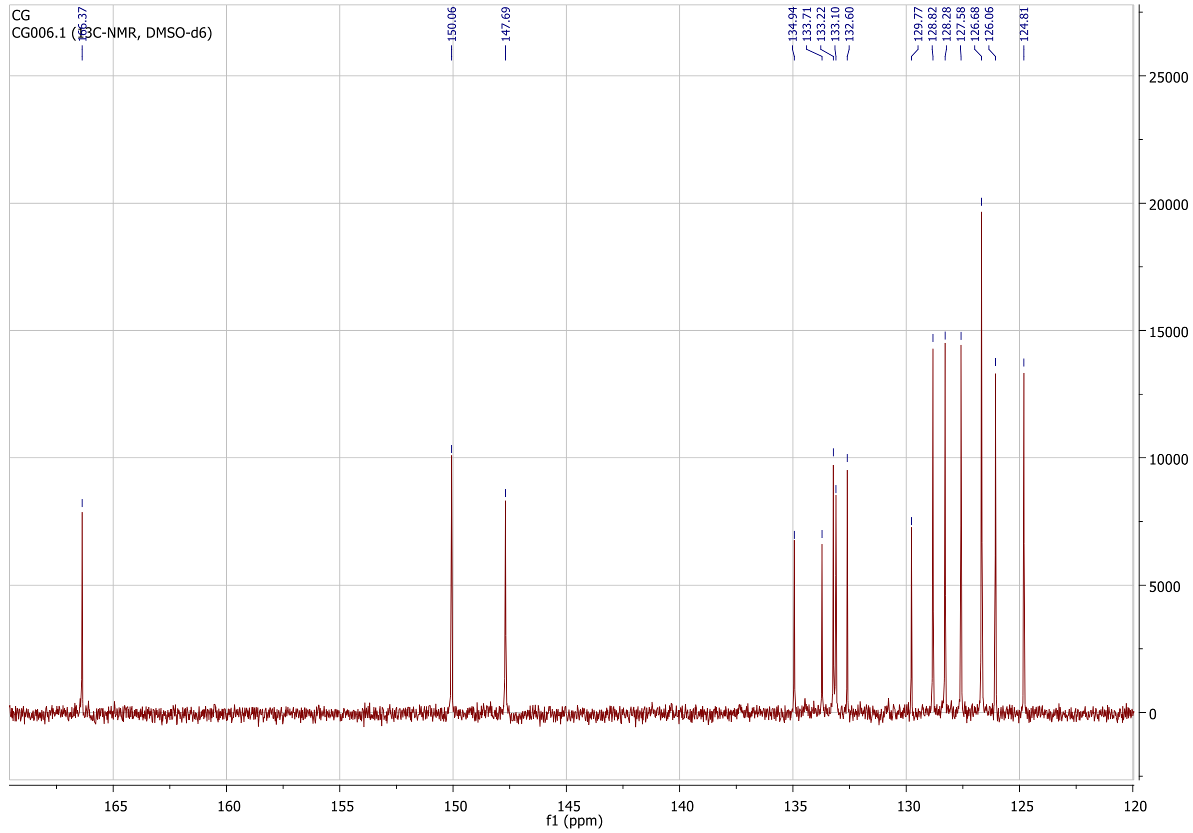

(*E*)-5-Styrylnicotinamide (**2b**)

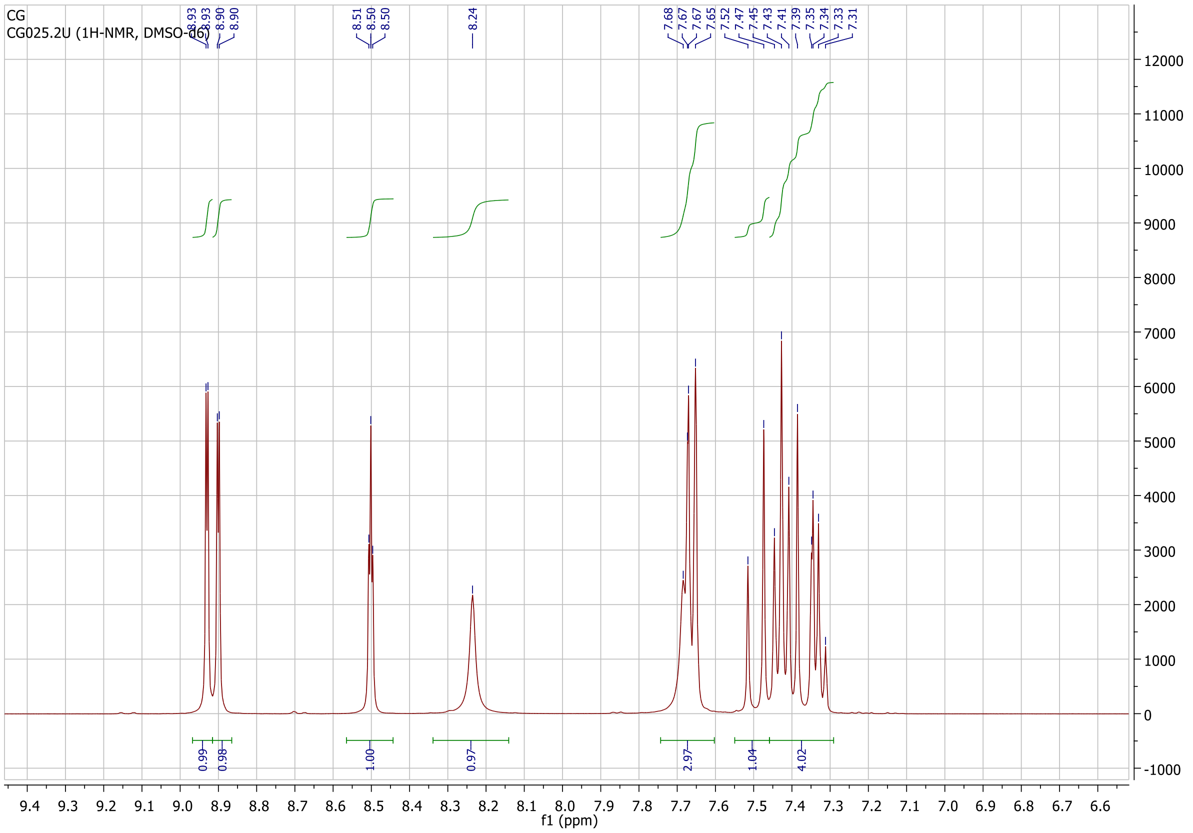

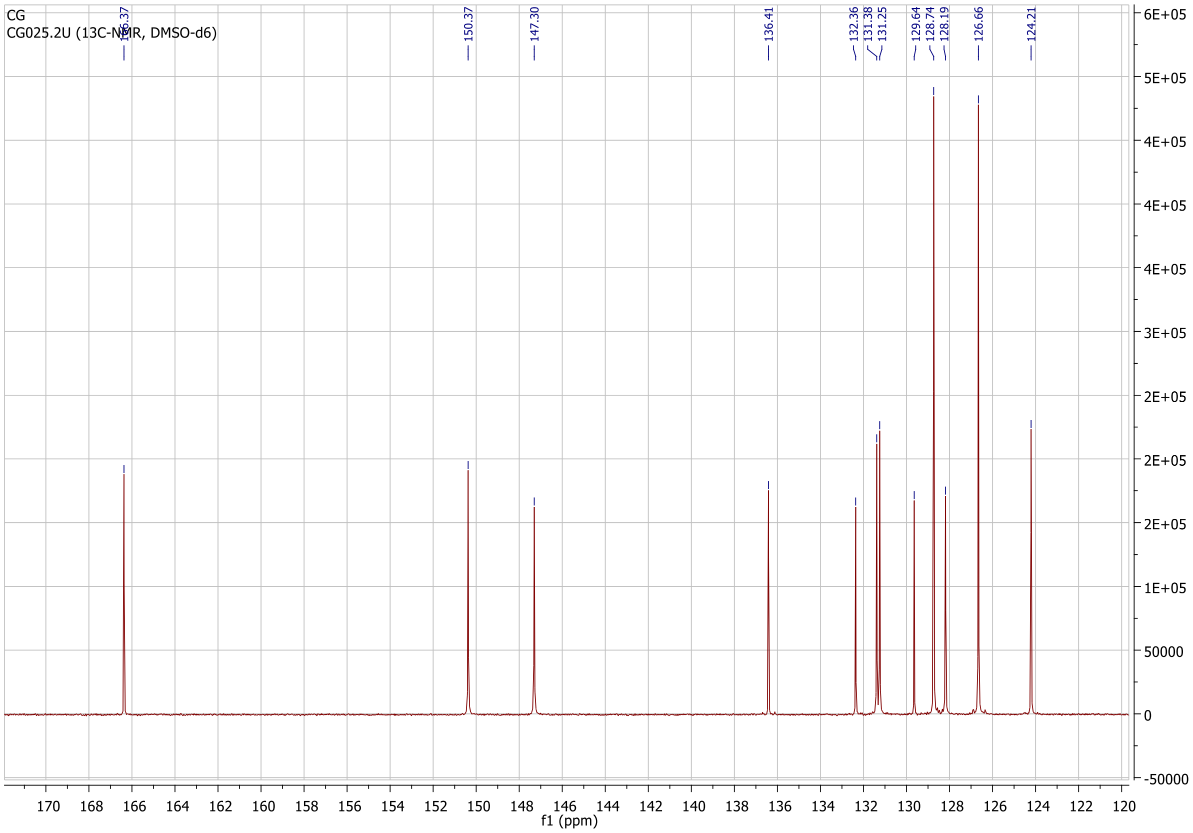

5-Vinylnicotinamide (**5a**)

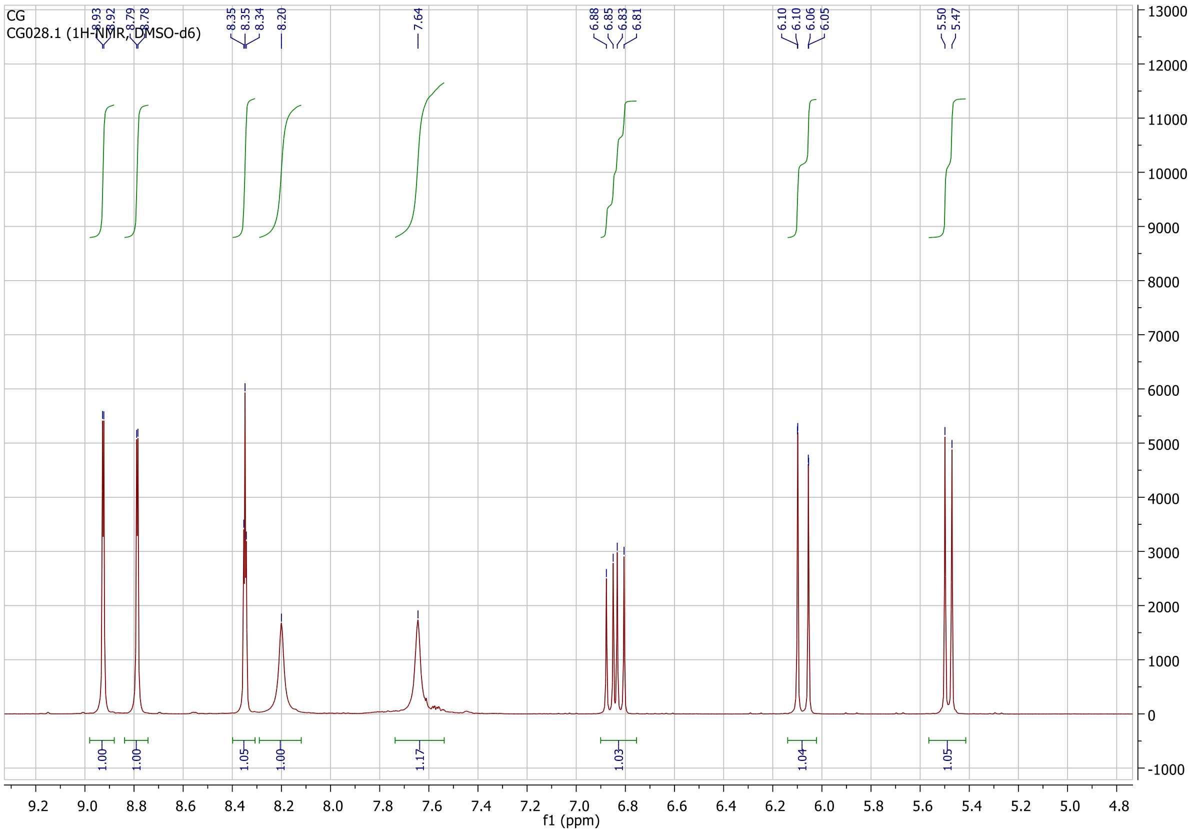

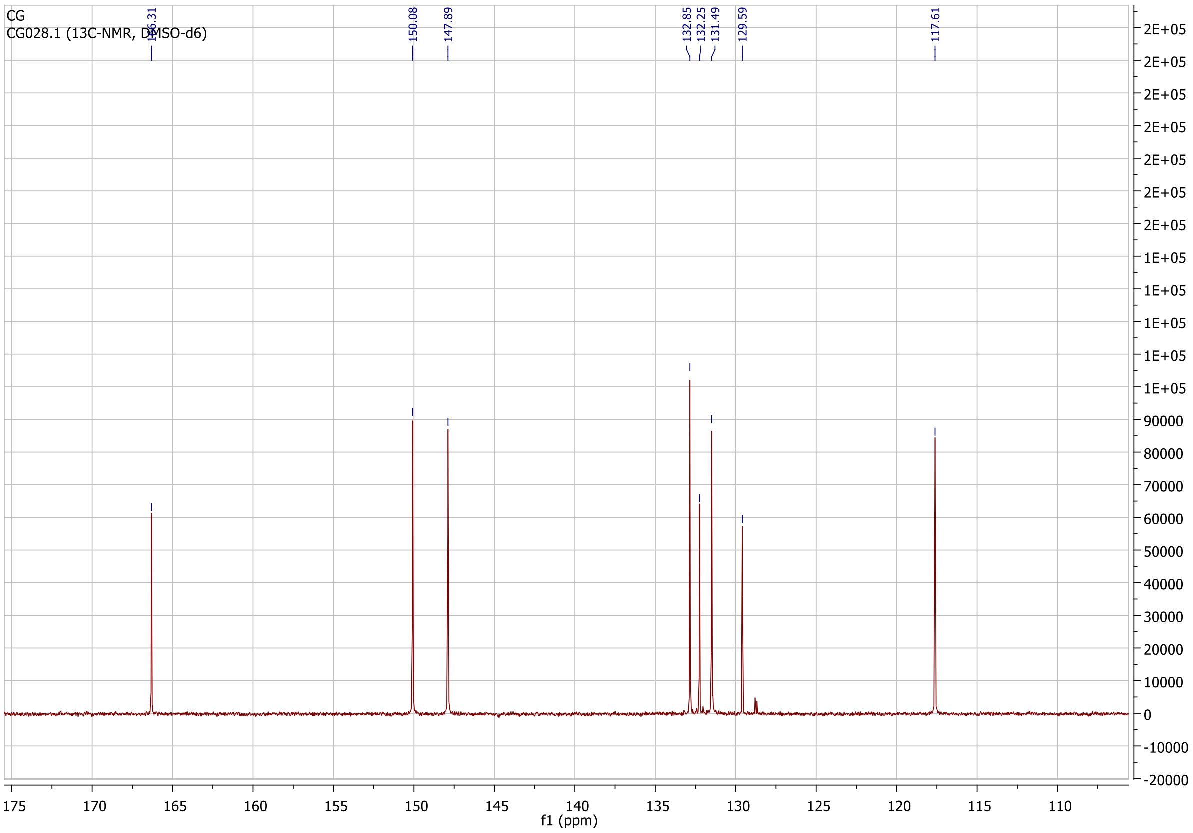

(*E*)-5-(4-Methoxystyryl)nicotinamide (**2d**)

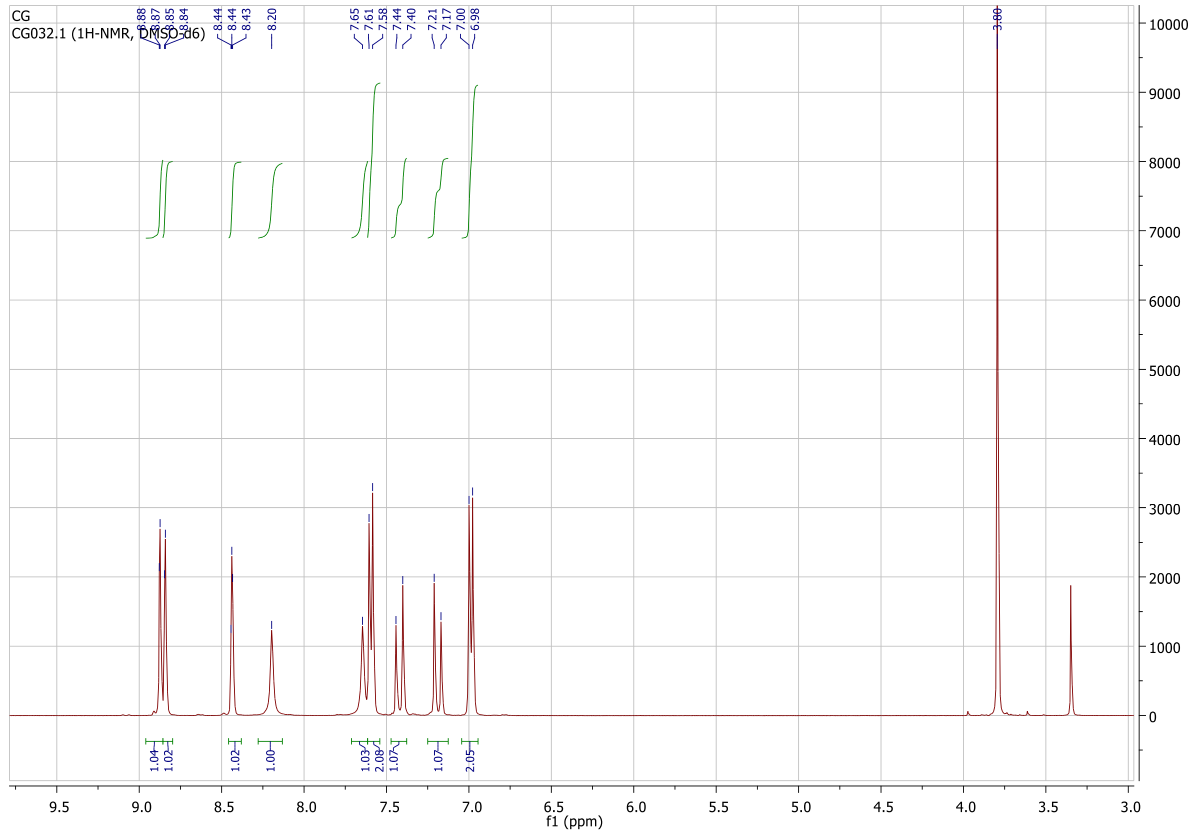

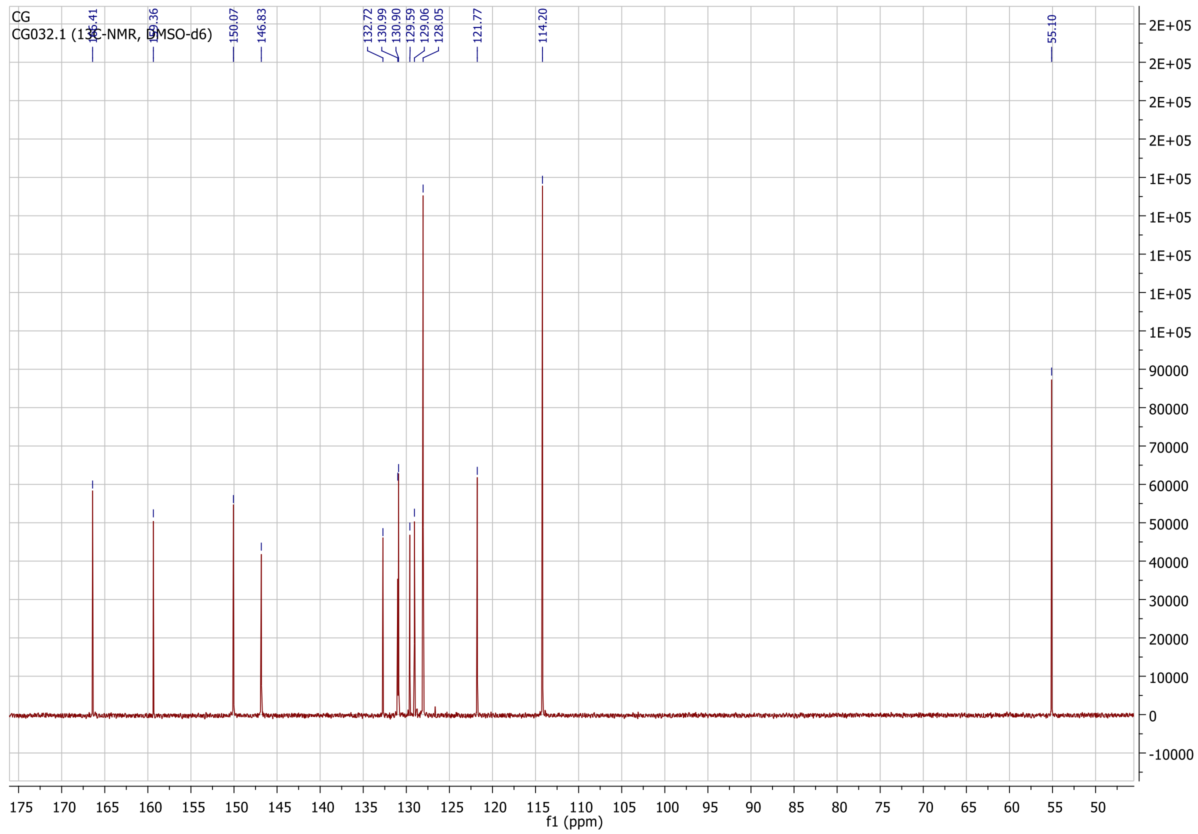

(*E*)-5-(4-Methoxy-2,6-dimethylstyryl)nicotinamide (**2f**)

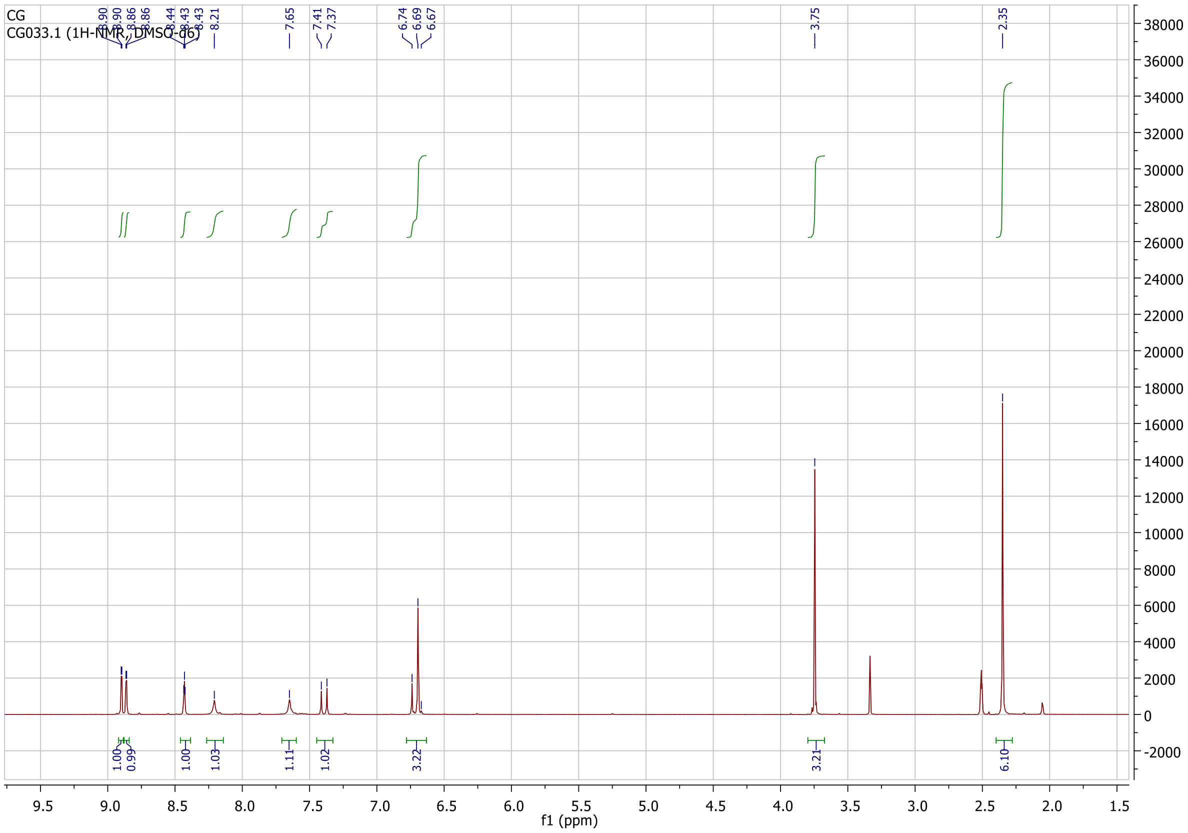

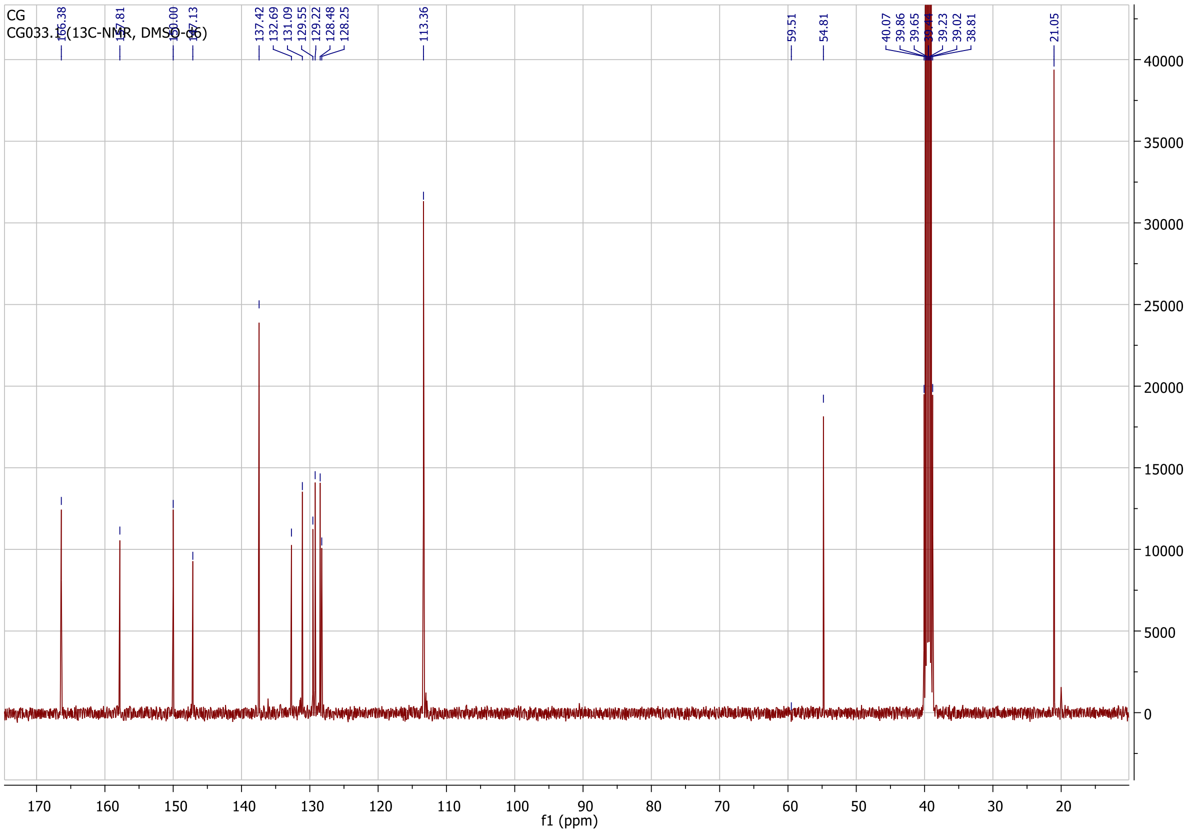

1-(Benzyloxy)-3-vinylbenzene

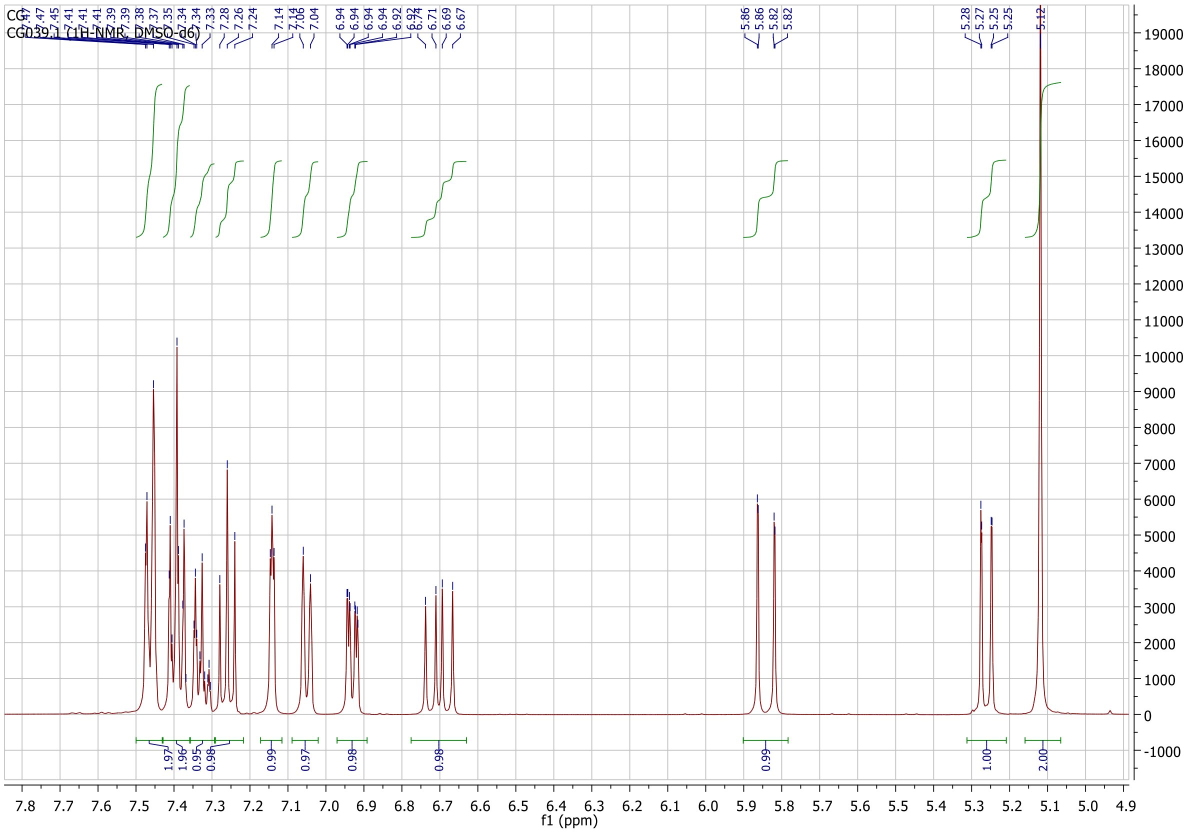

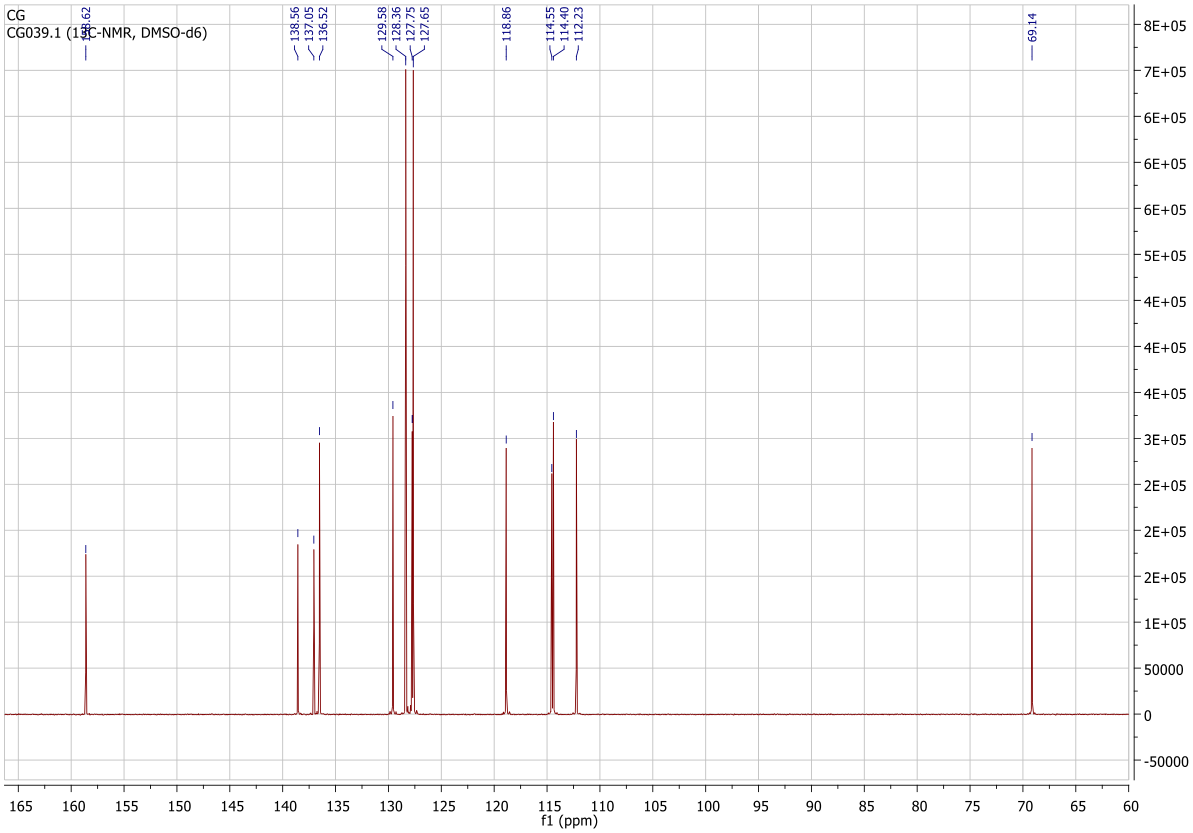

(*E*)-5-[3-(Benzyloxy)styryl]nicotinamide (**2e**)

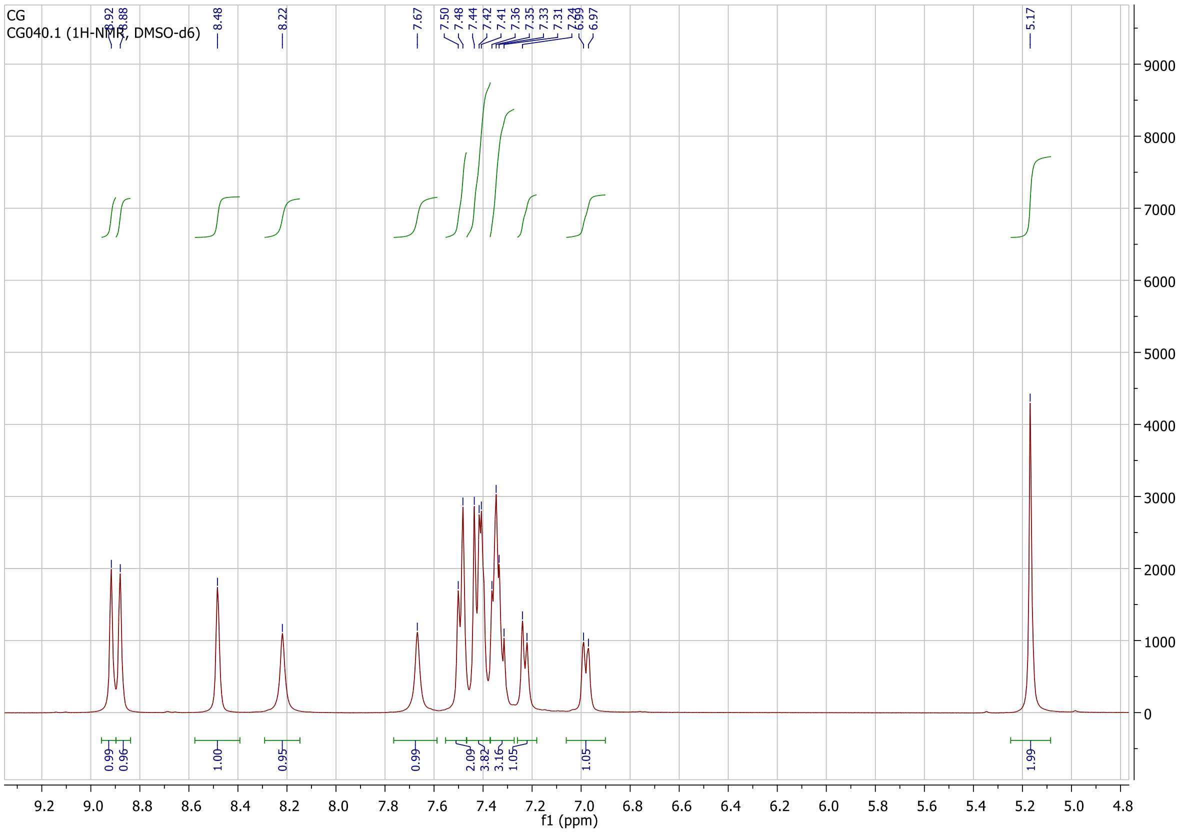

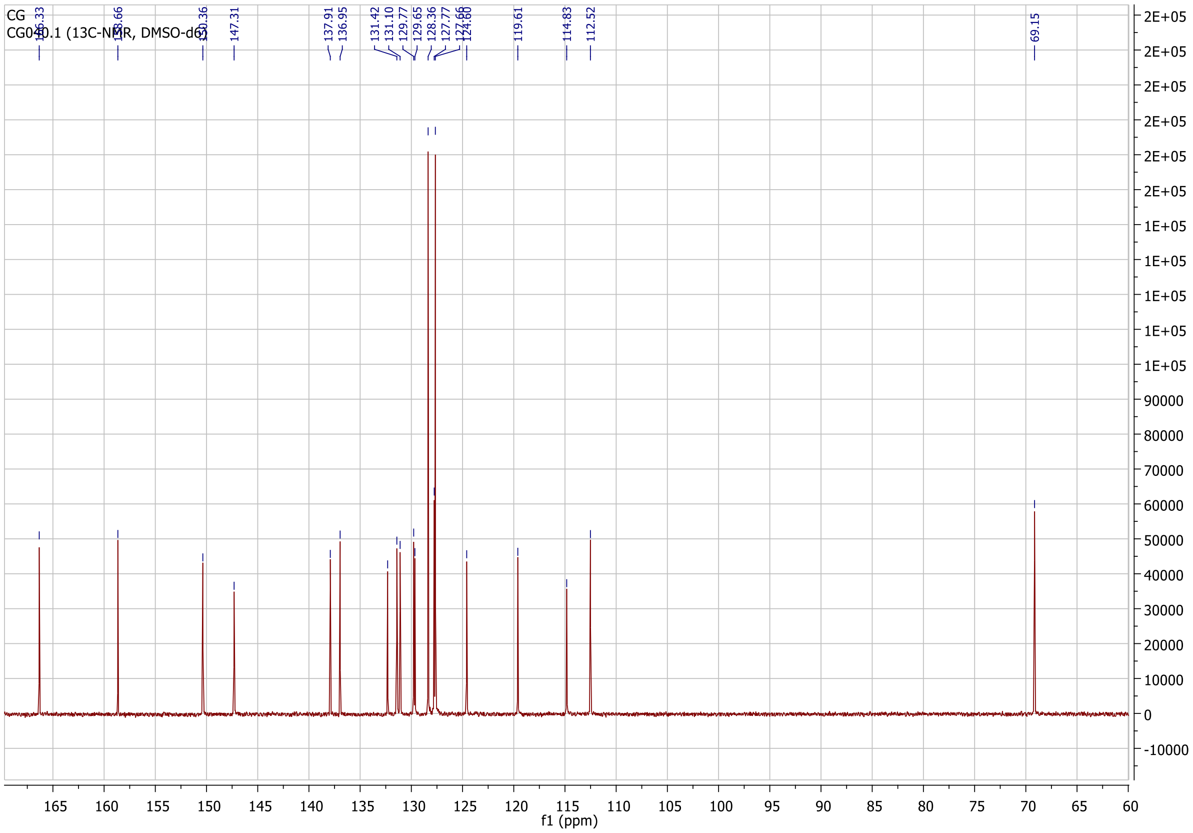

Methyl 5-vinylnicotinate (**5b**)

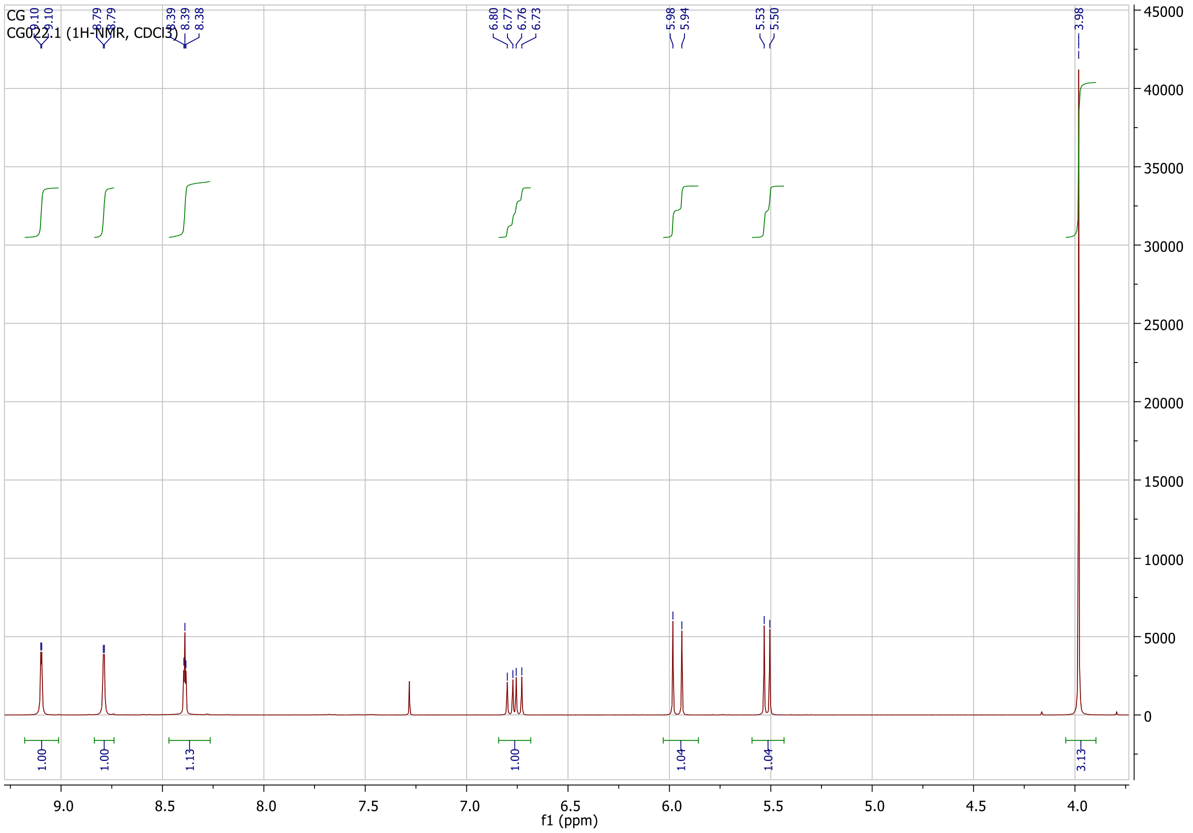

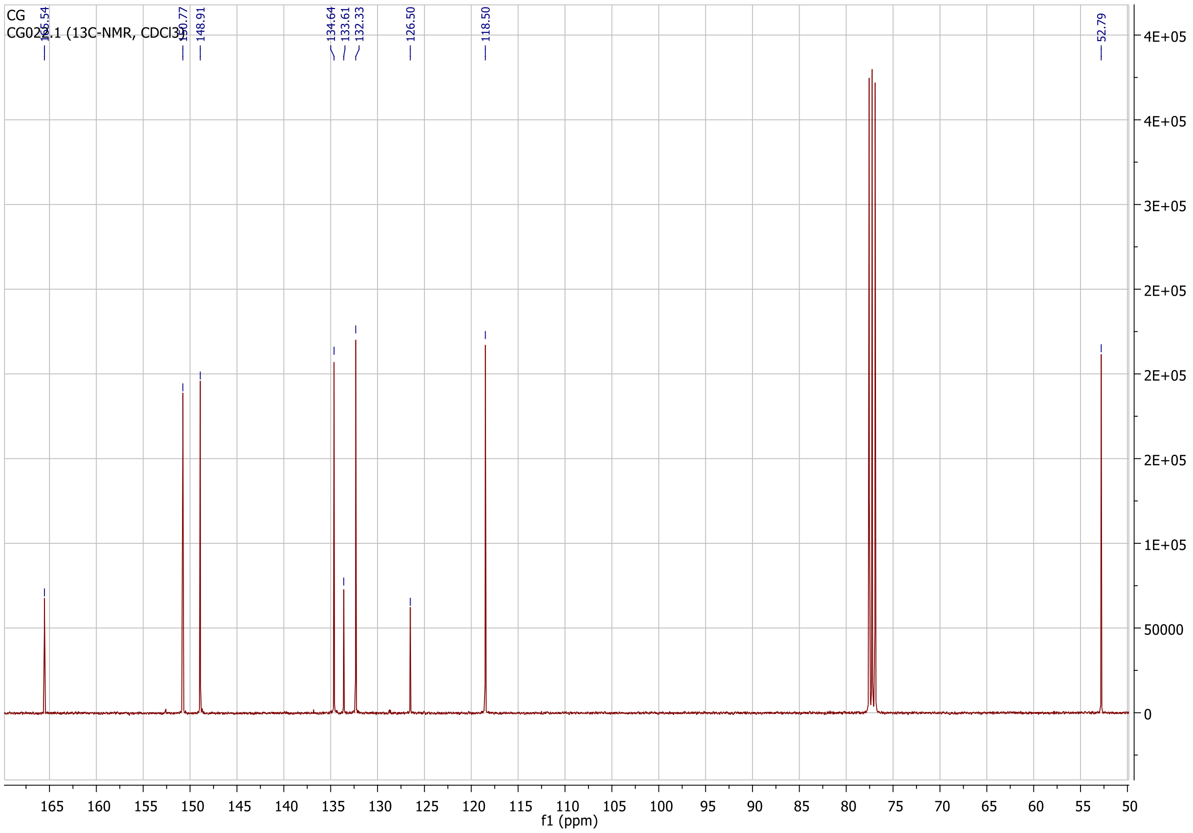

4-(Benzyloxy)-*N*-(3-bromophenyl)benzamide

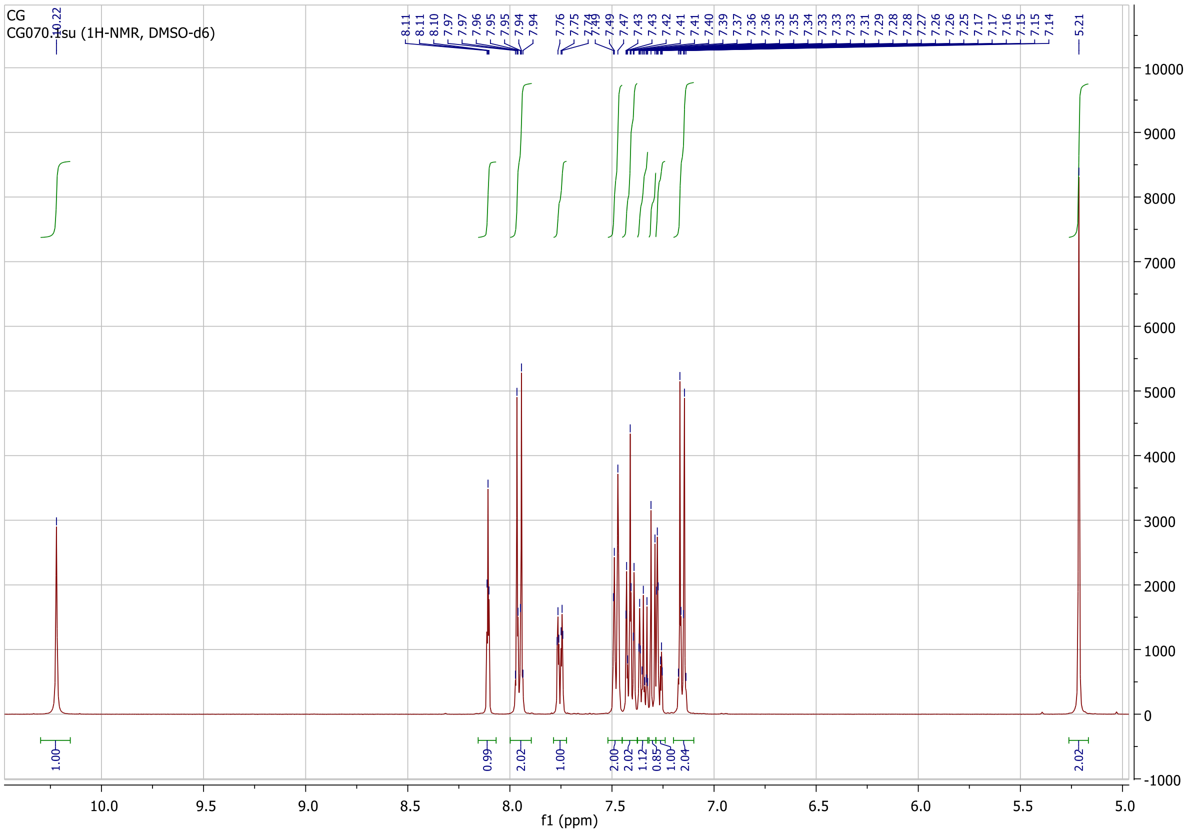

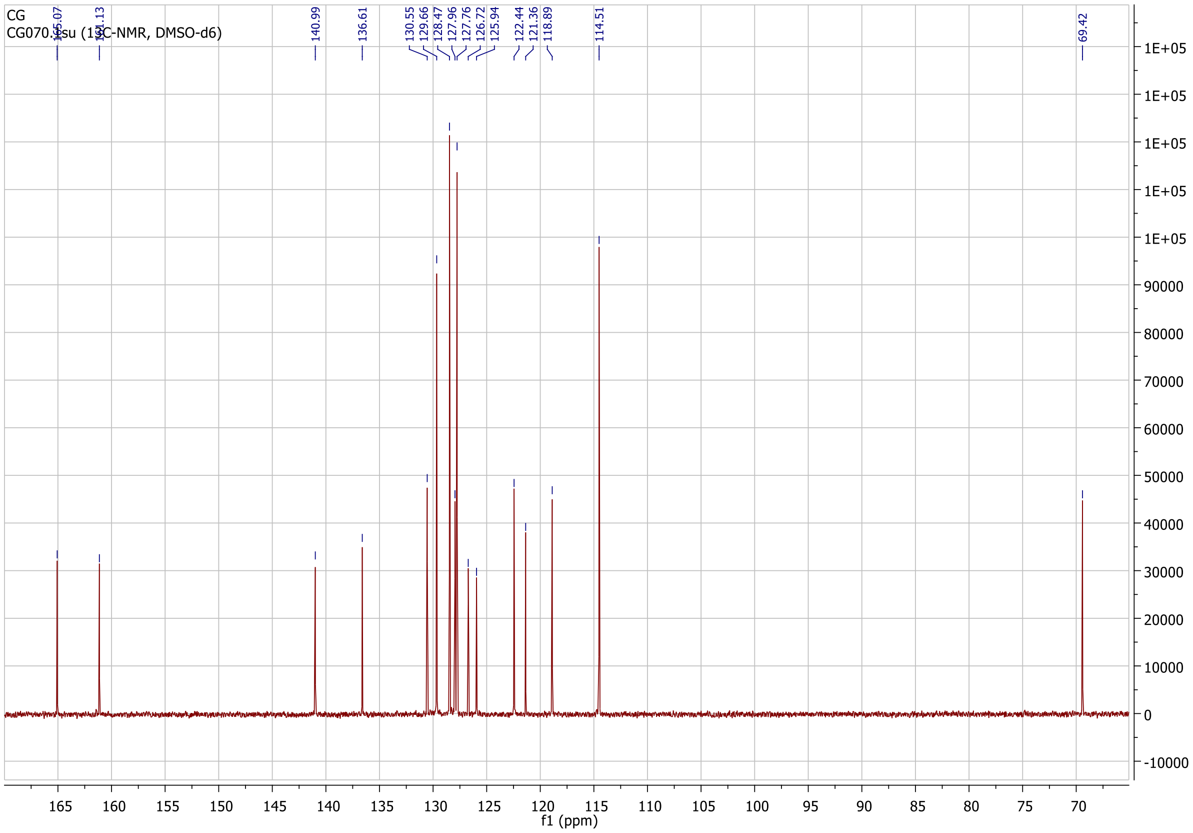

Methyl (*E*)-5-{3-[4-(benzyloxy)benzamido]styryl}nicotinate

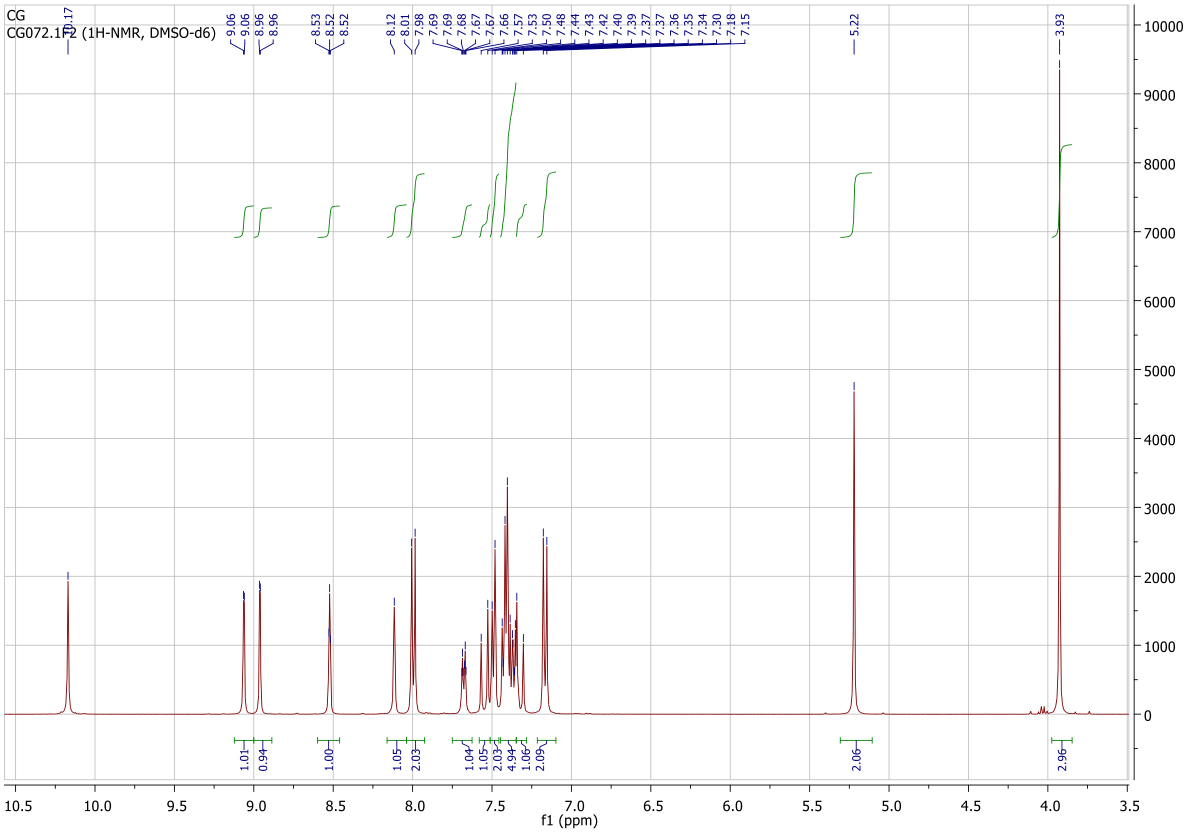

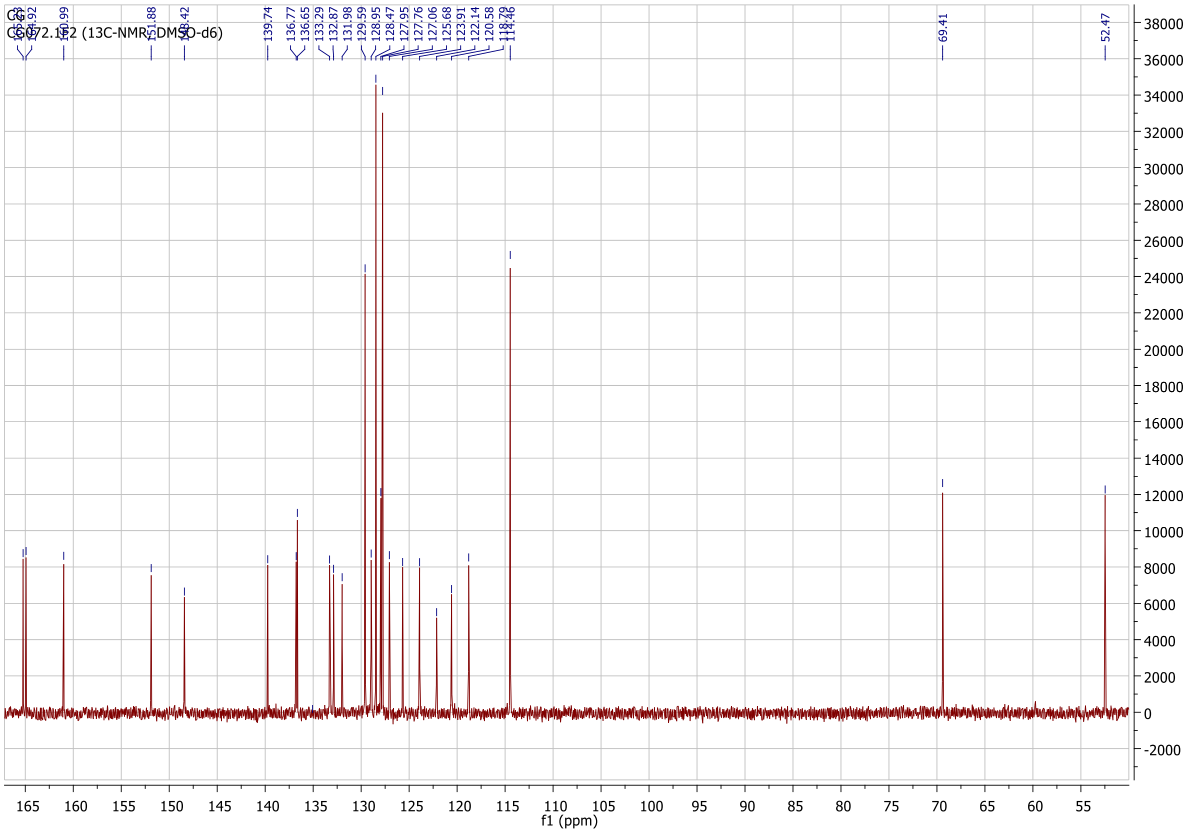

1-Fluoro-4-vinylbenzene

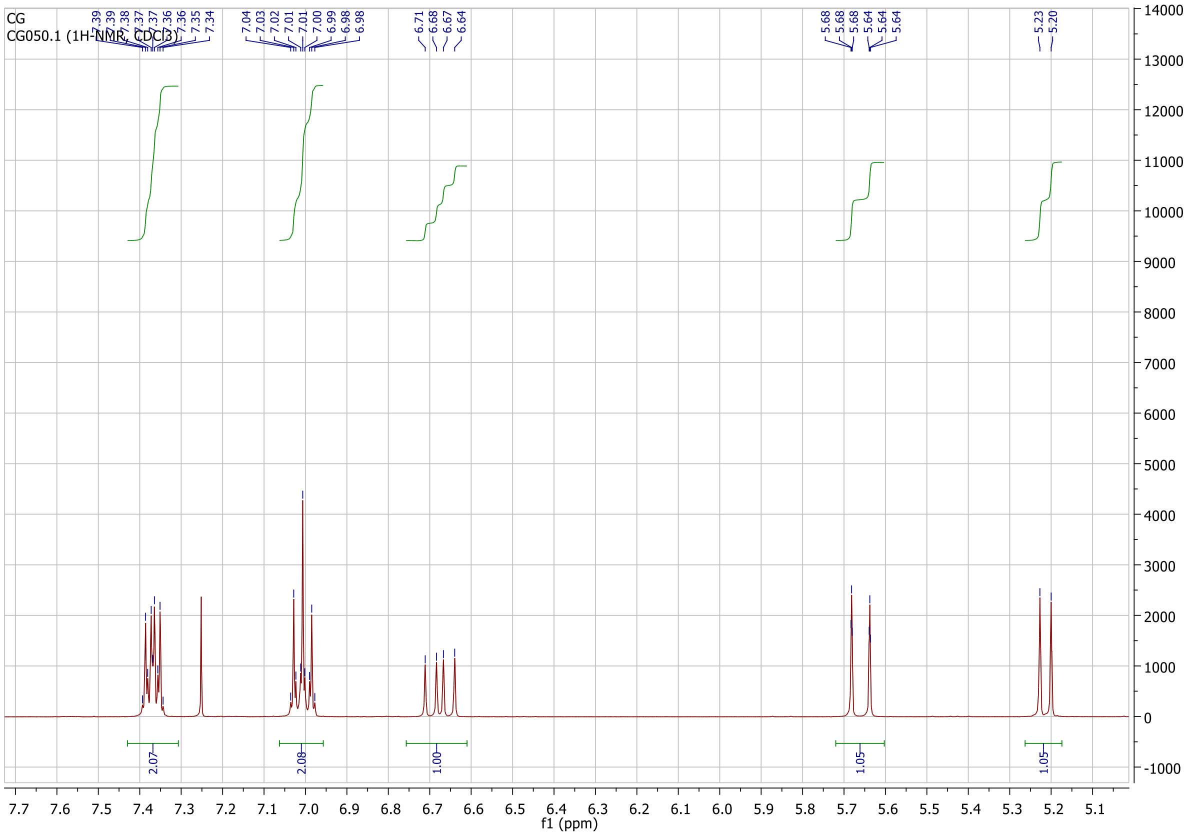

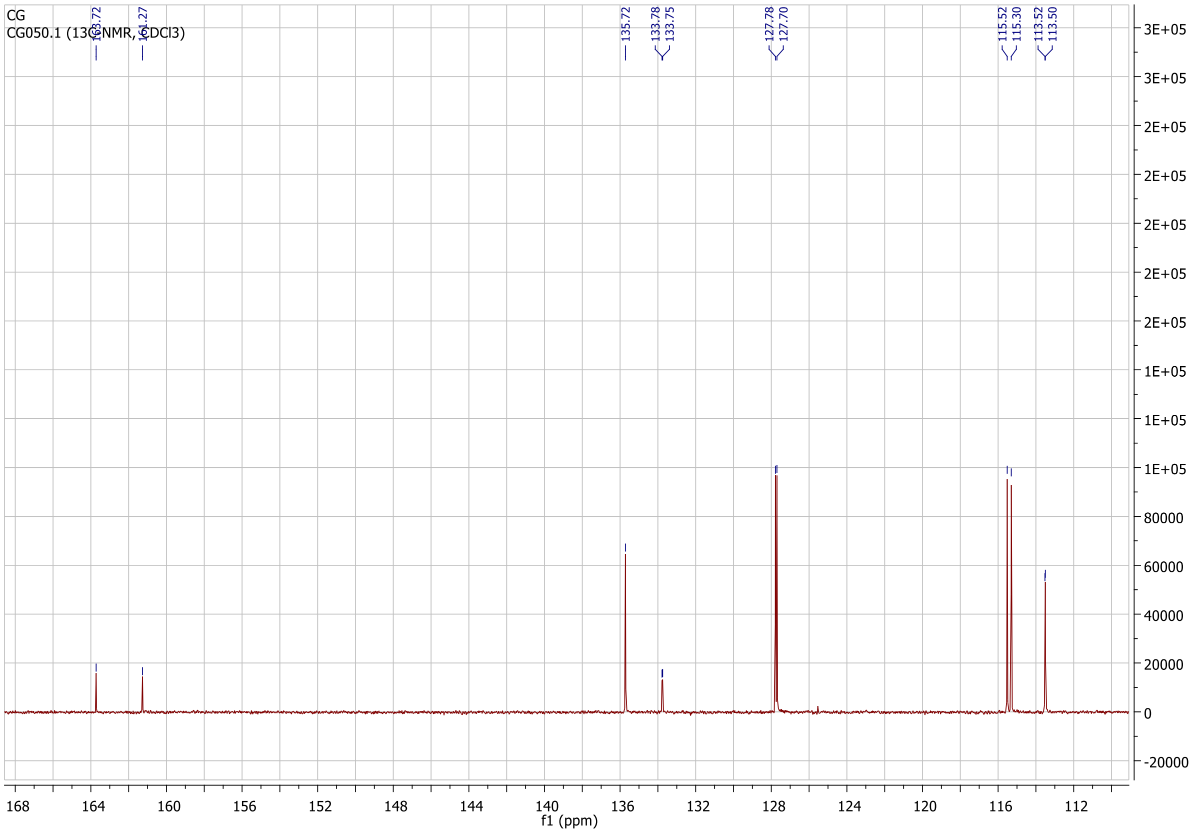

Methyl (*E*)-5-(4-fluorostyryl)nicotinate

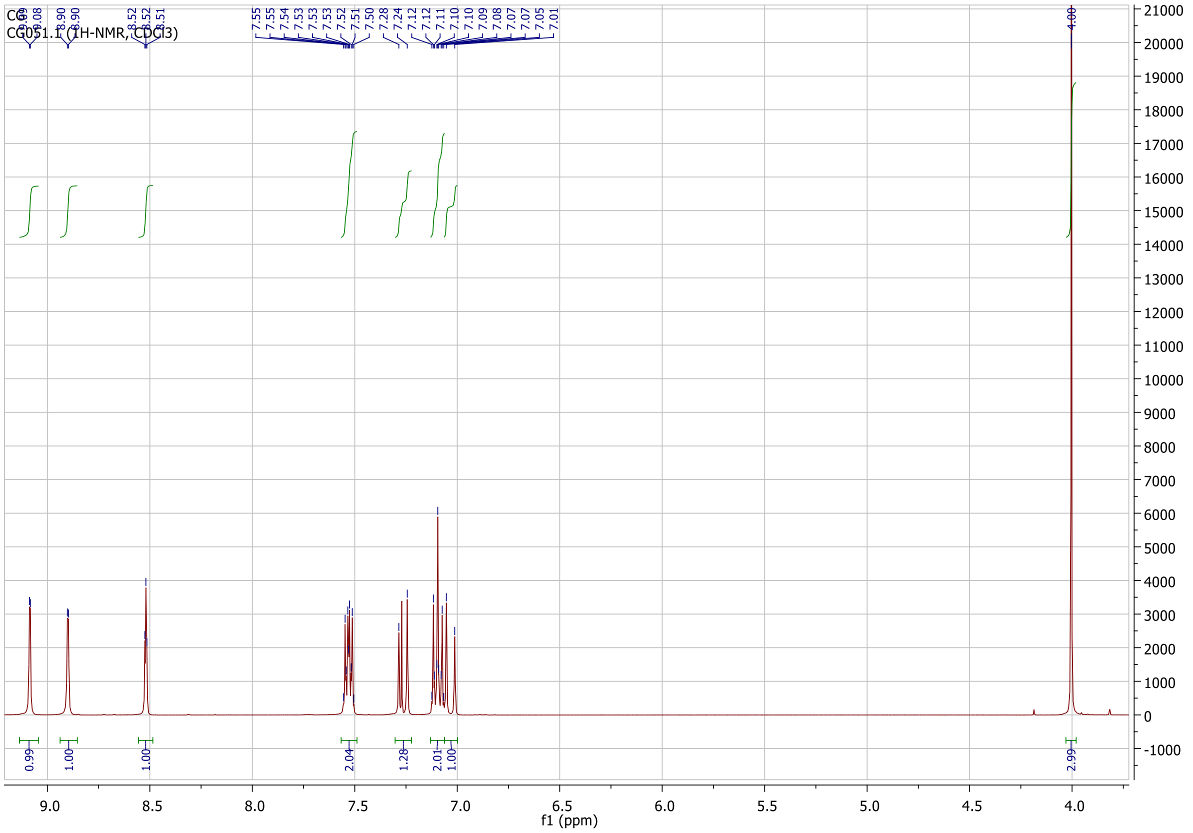

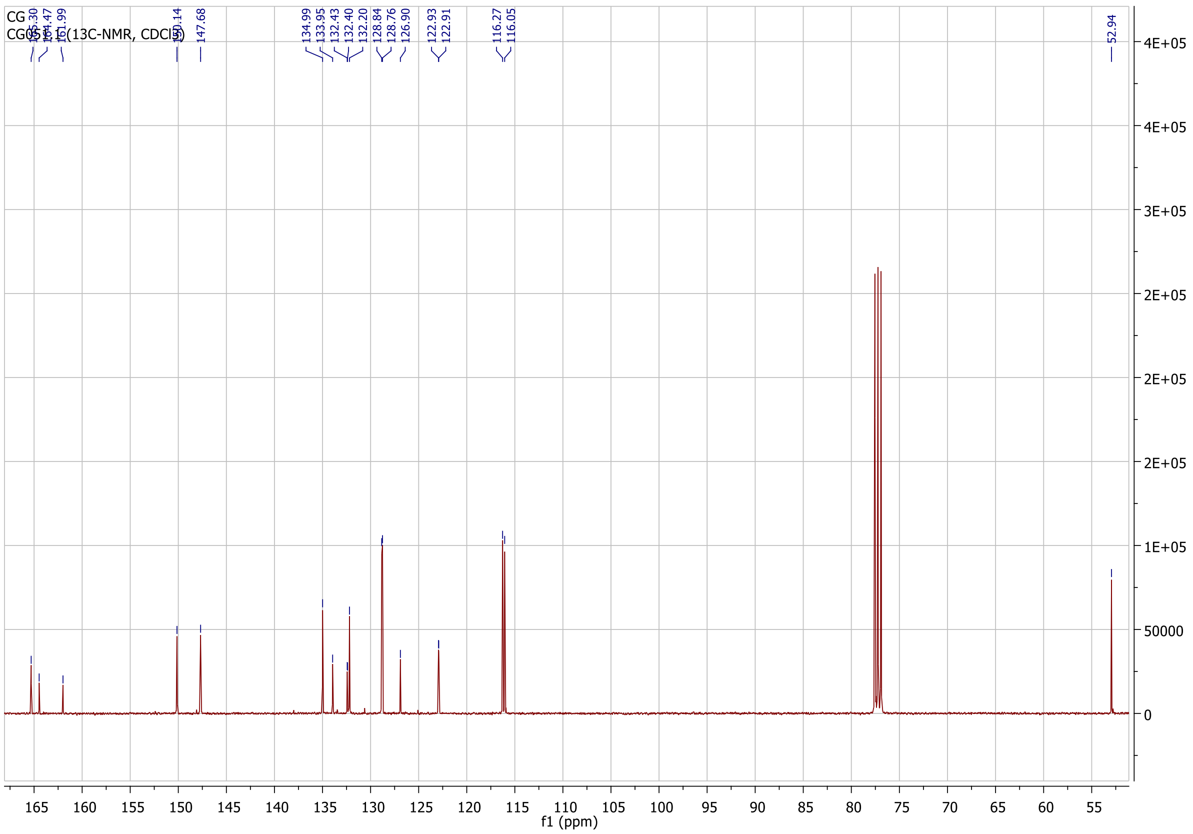

5-Vinyl-1,3-benzodioxole

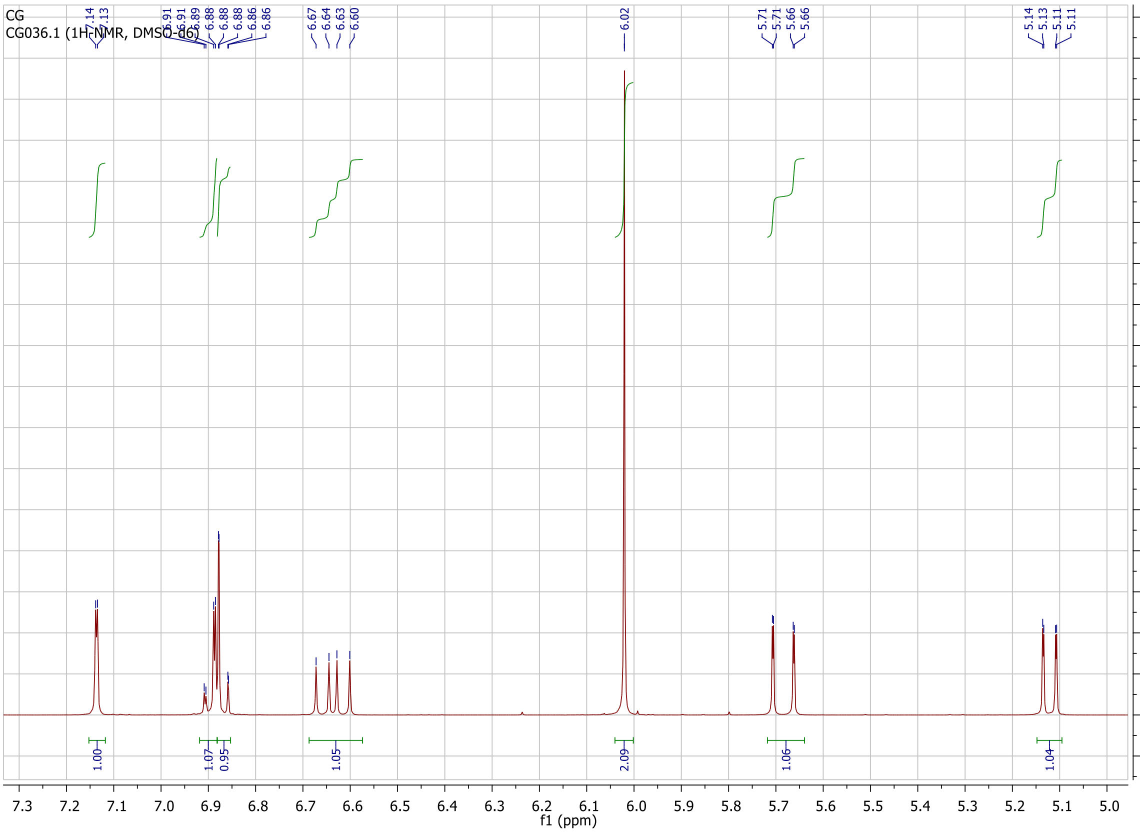

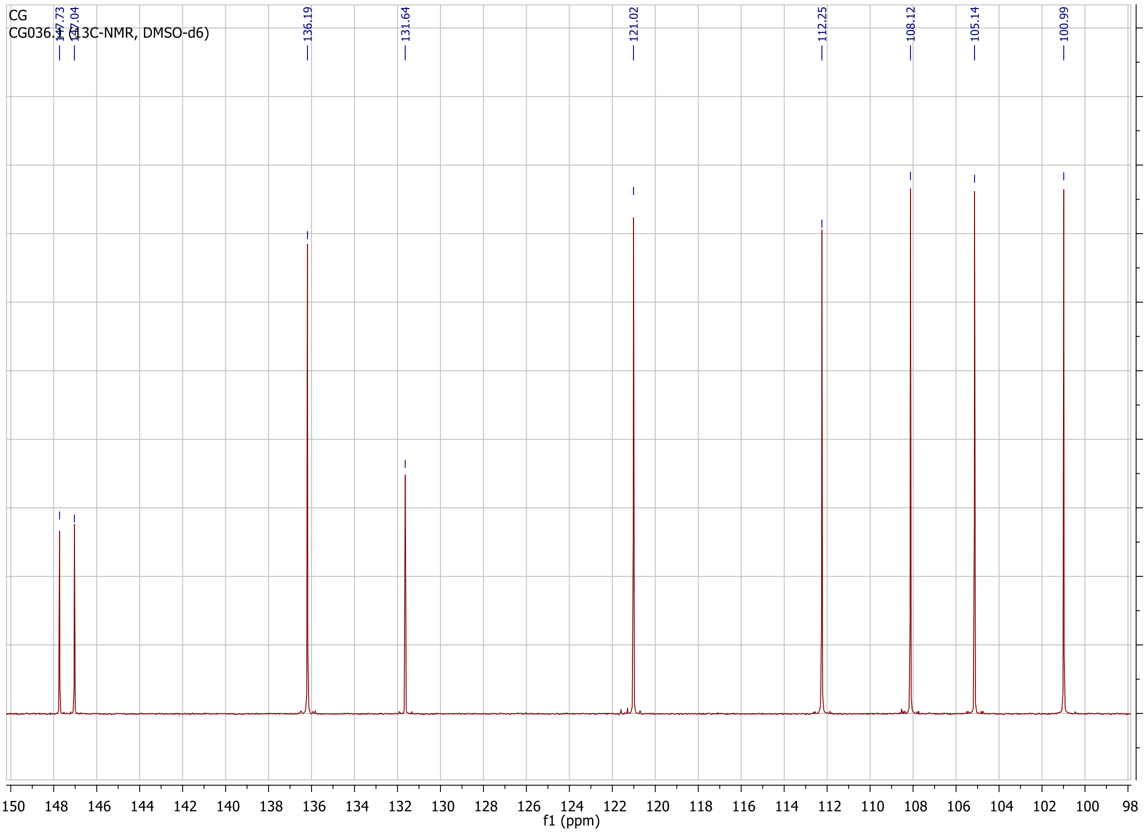

Methyl (*E*)-5-[2-(benzo-1,3-dioxol-5-yl)vinyl]nicotinate

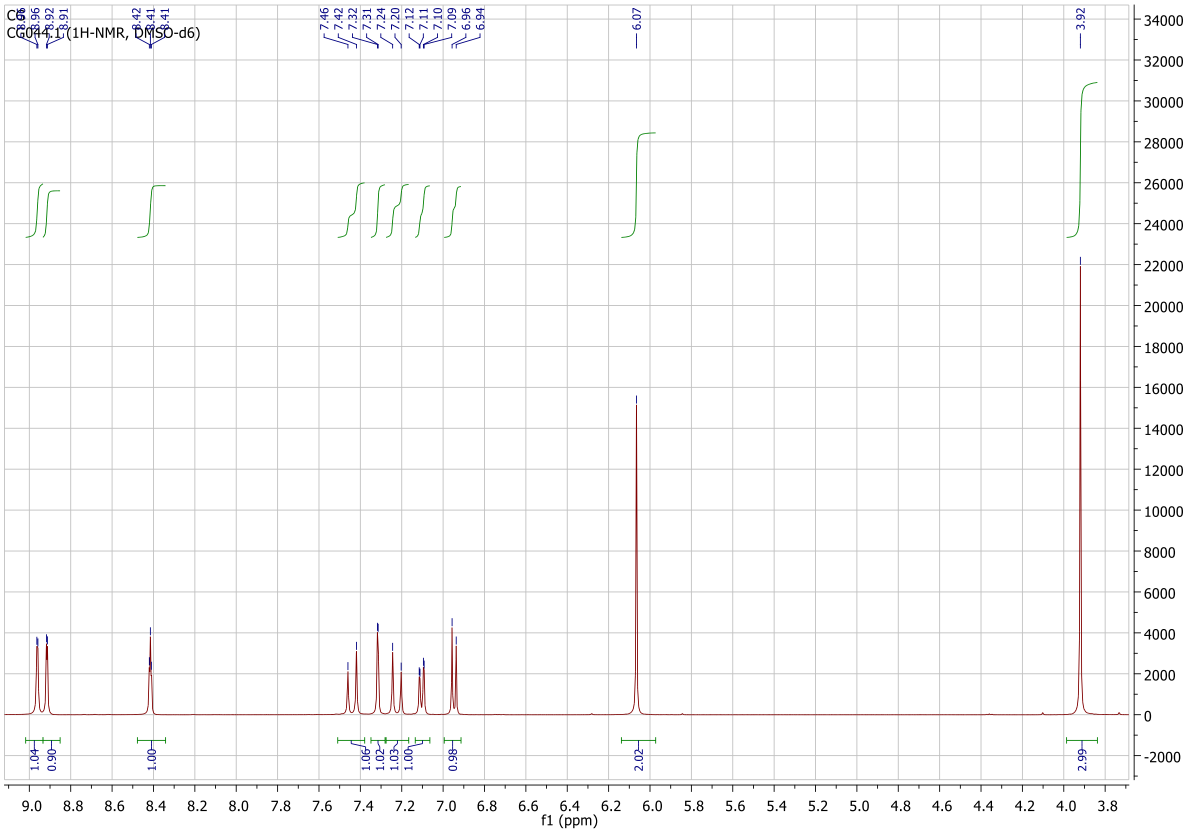

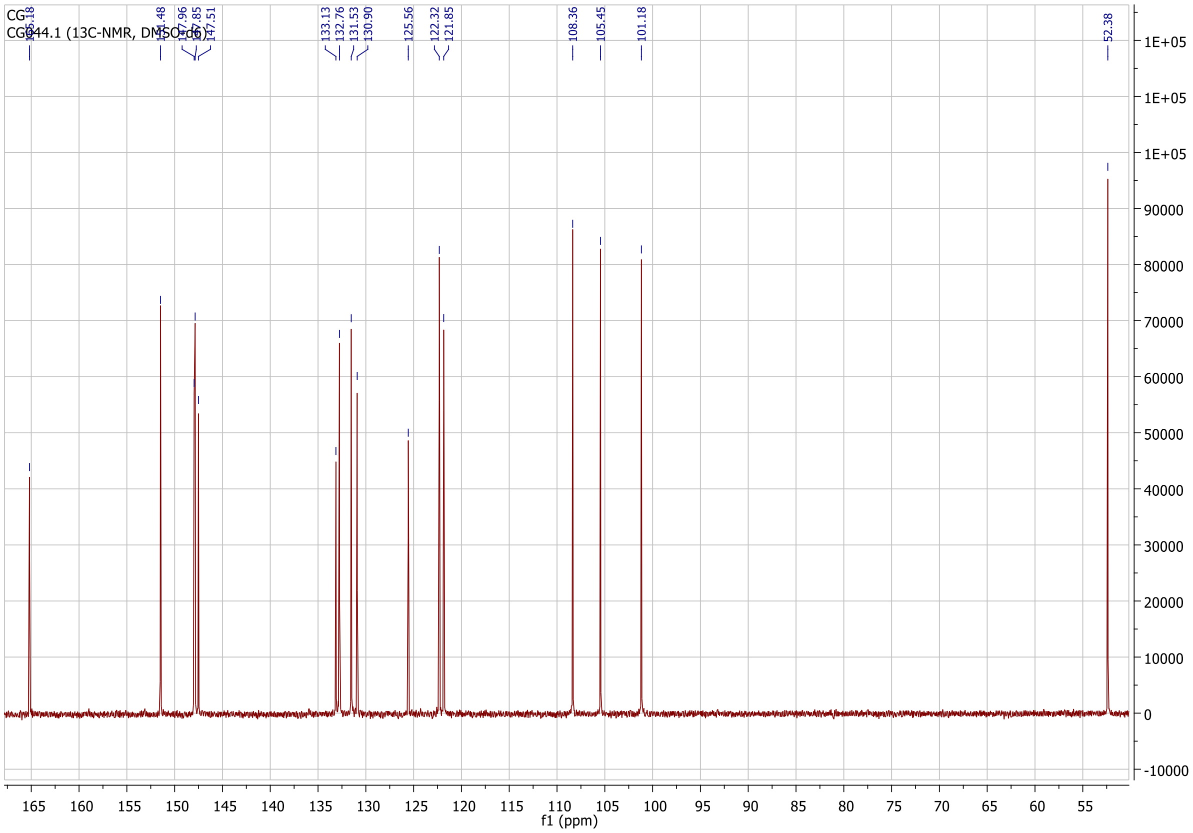

(*E*)-5-(4-fluorostyryl)nicotinamide (**2c**)

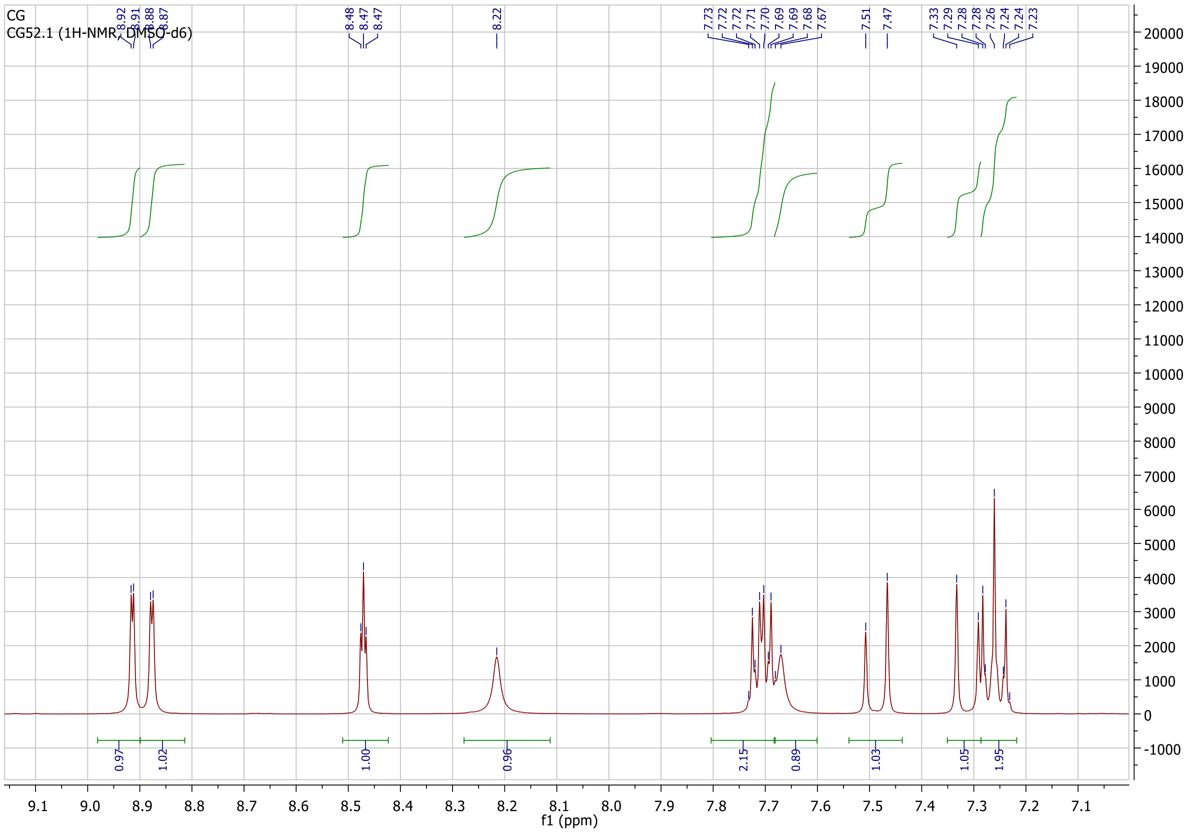

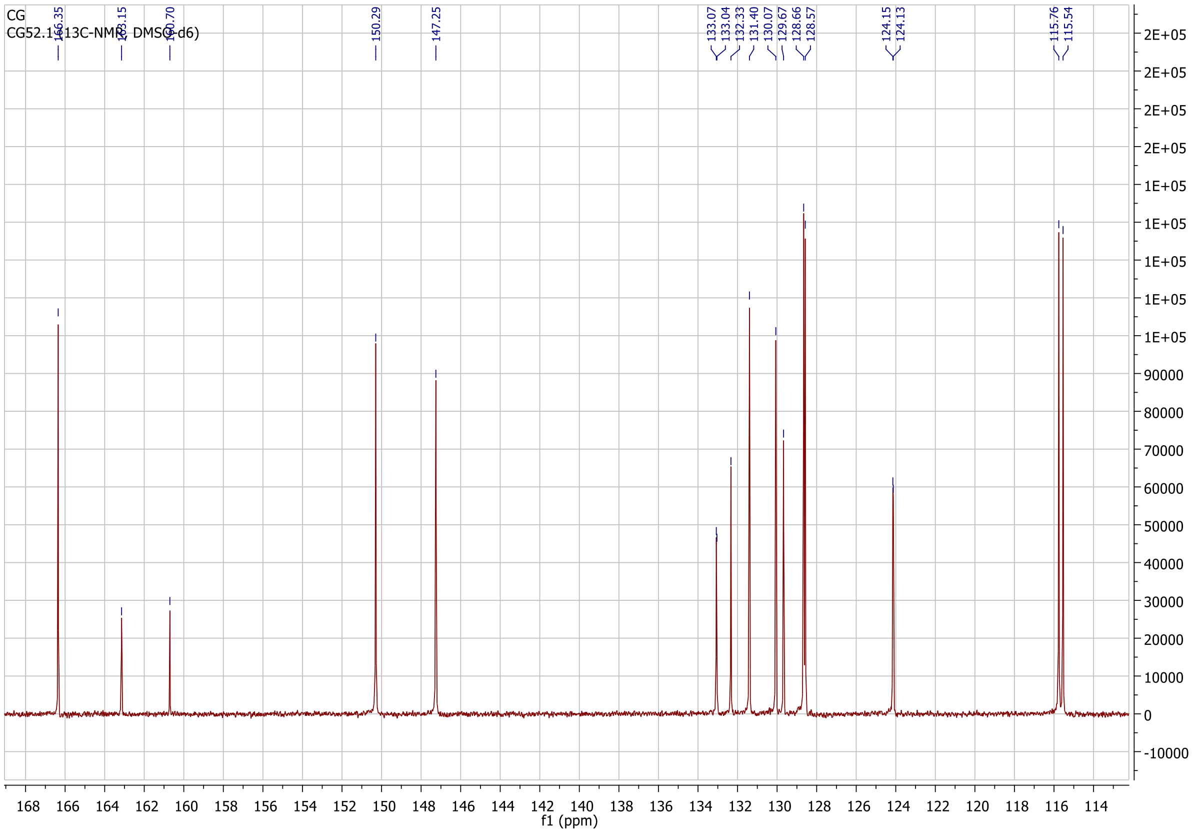

(*E*)-5-[2-(benzodioxol-5-yl)vinyl]nicotinamide (**2g**)

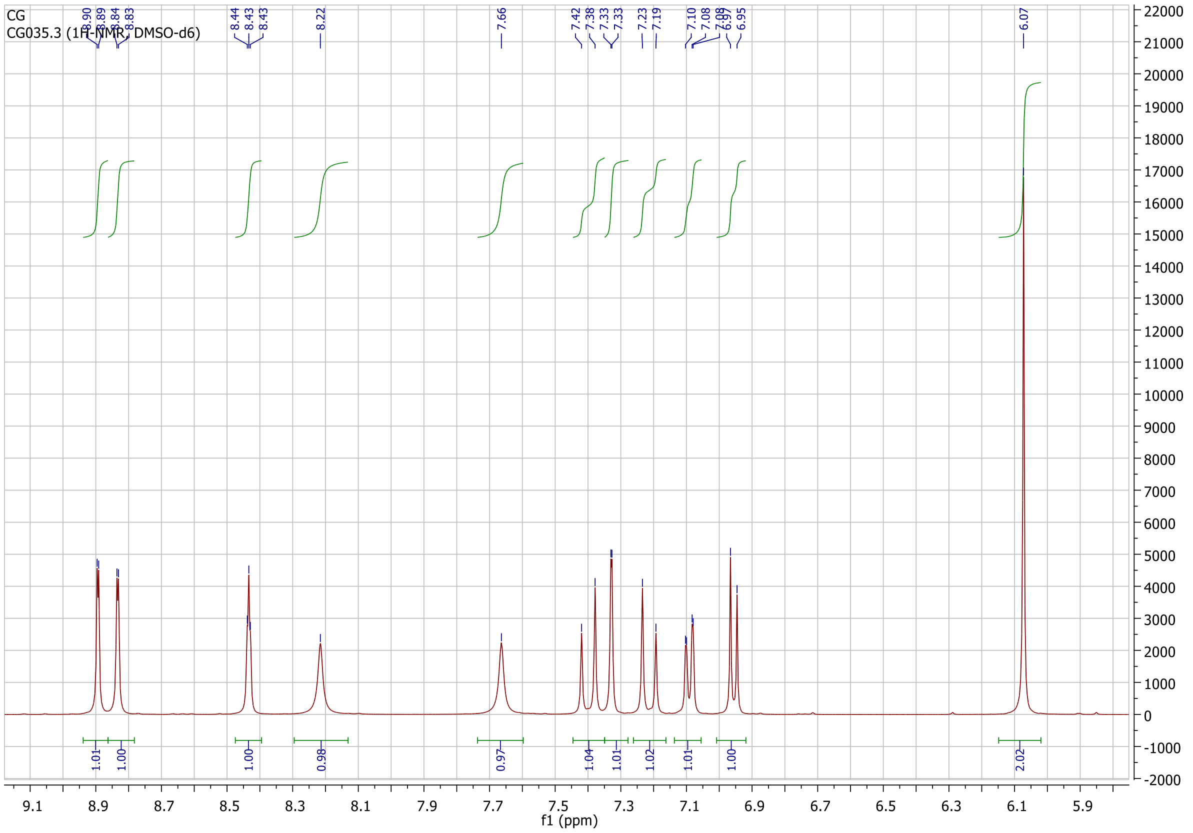

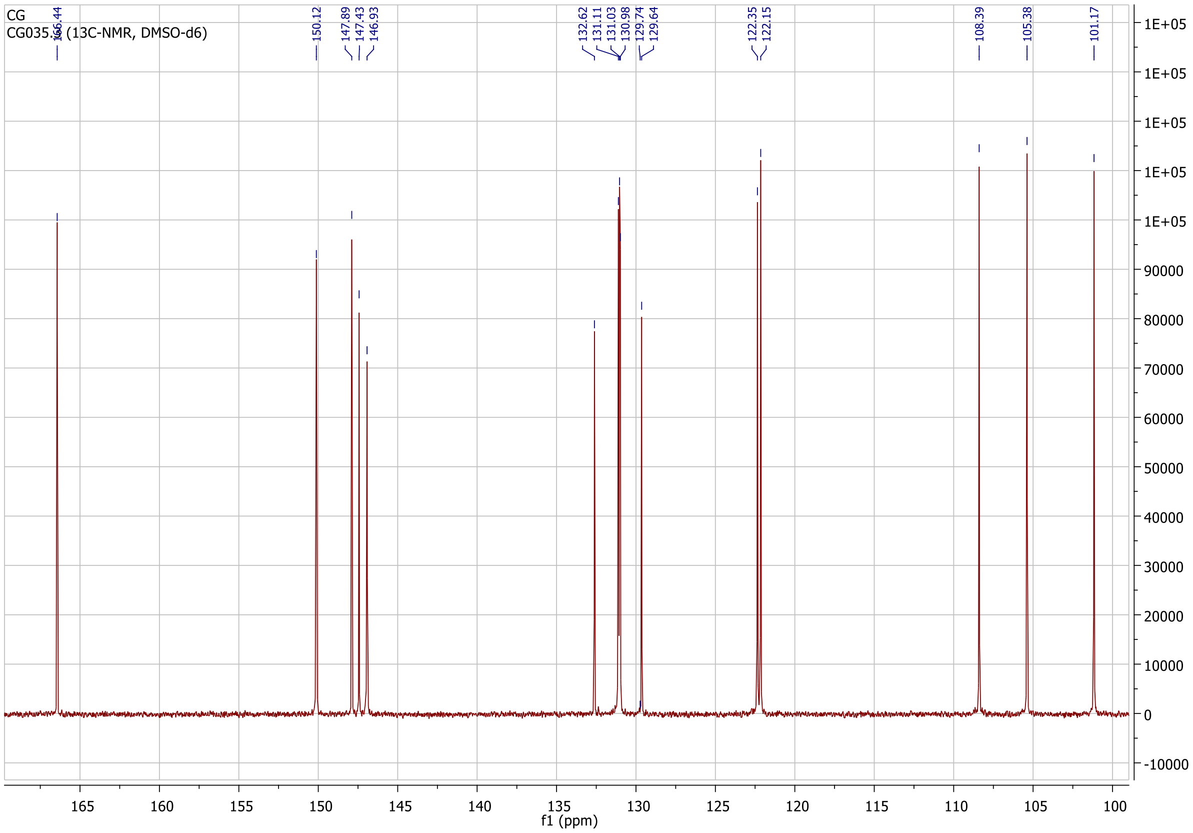

(*E*)-5-{3-[4-(benzyloxy)benzamido]styryl}nicotinamide (**2h**)

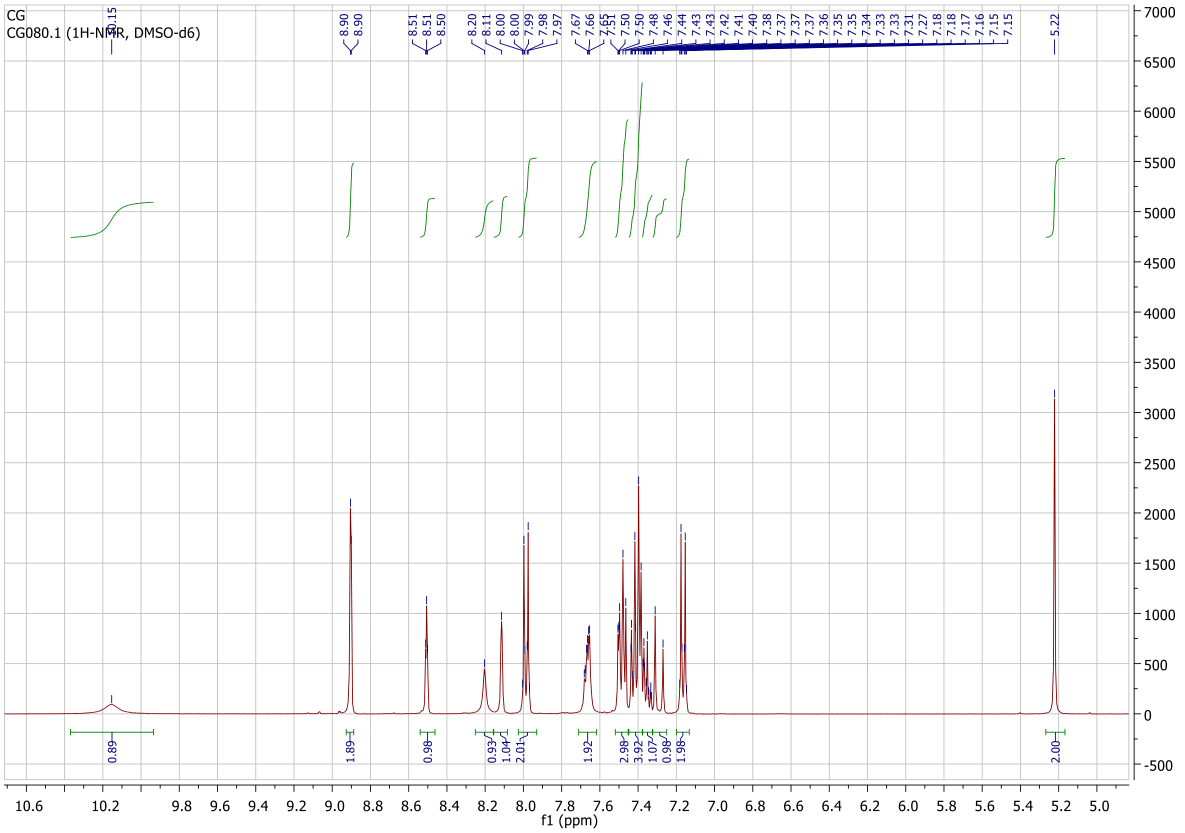

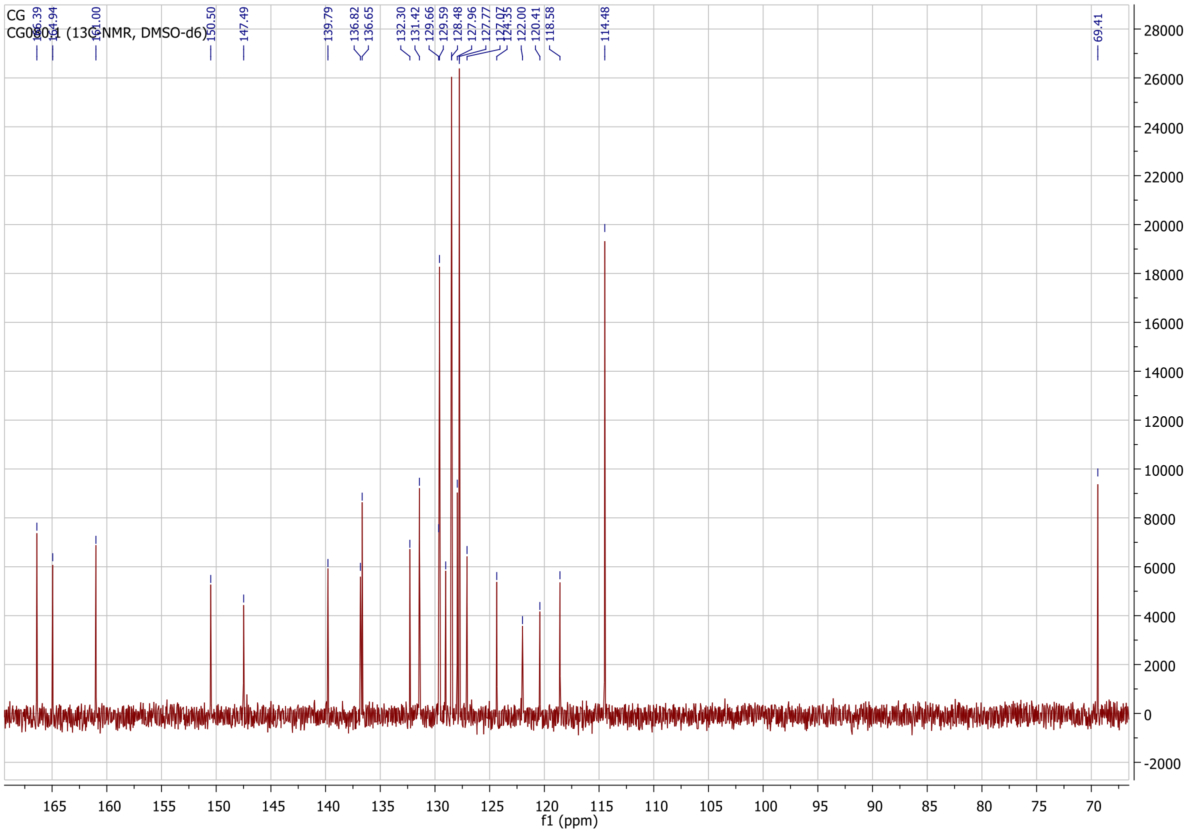

Methyl 5-[(4-fluorophenyl)diazenyl]nicotinate (**10**)

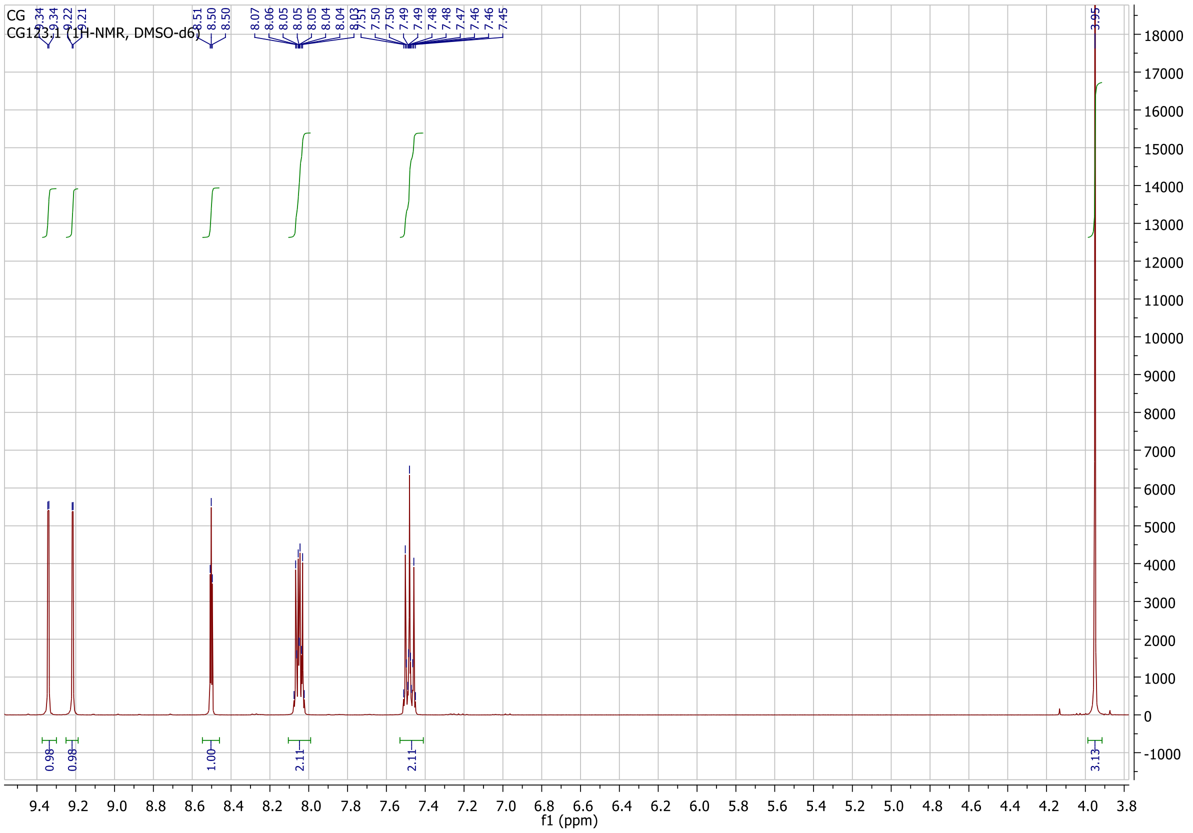

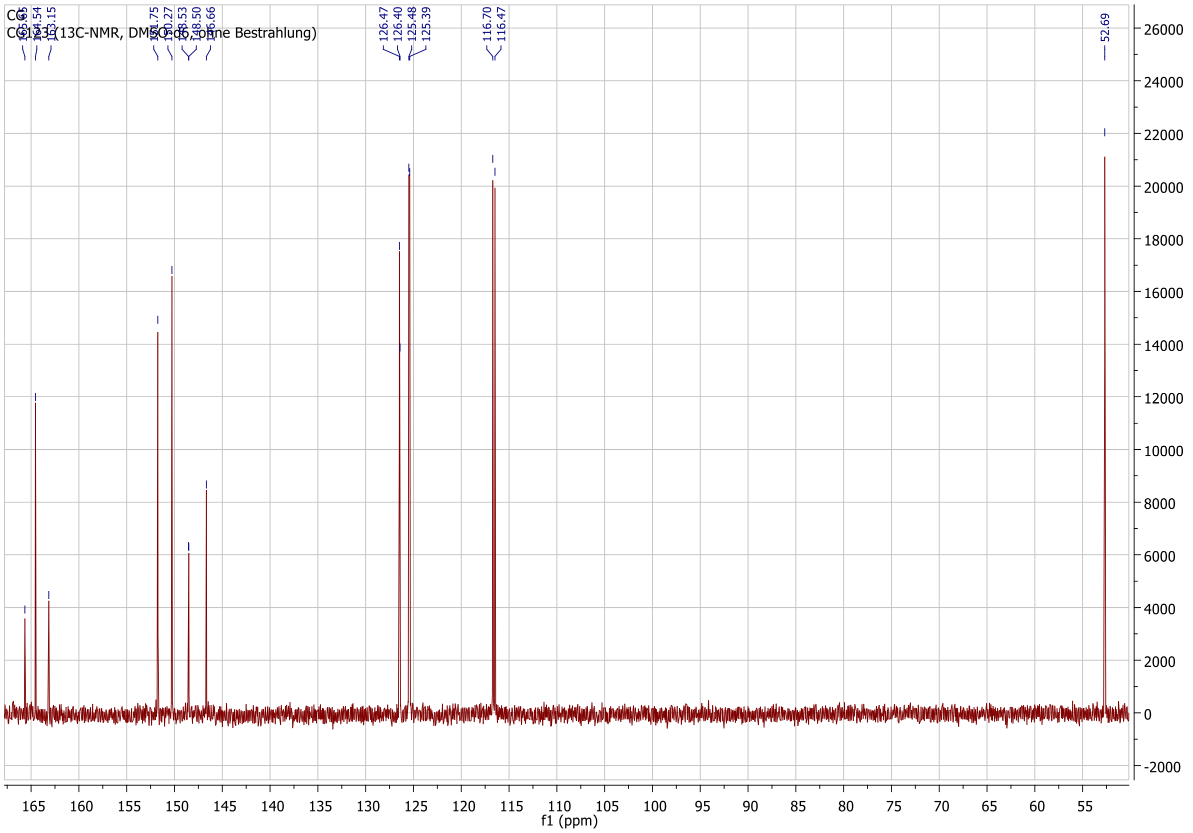

5-[(4-Fluorophenyl)diazenyl]nicotinamide (**11**)

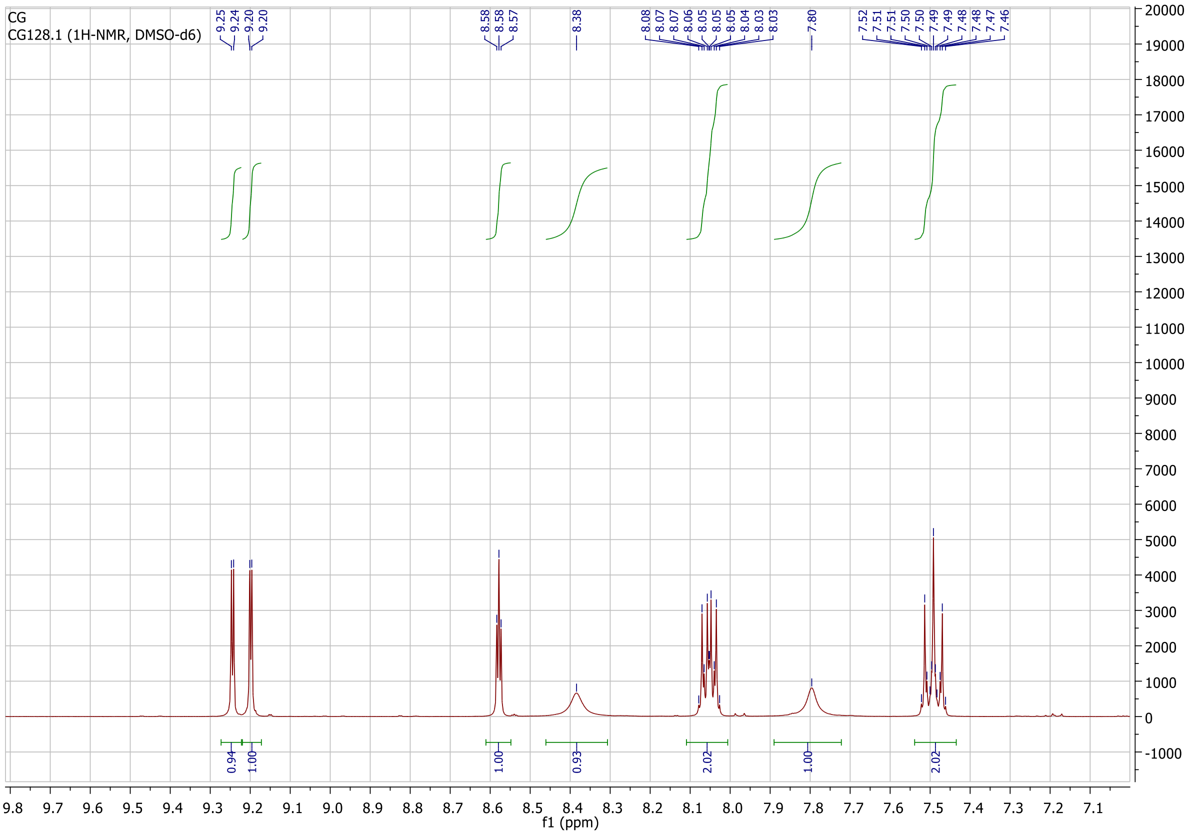

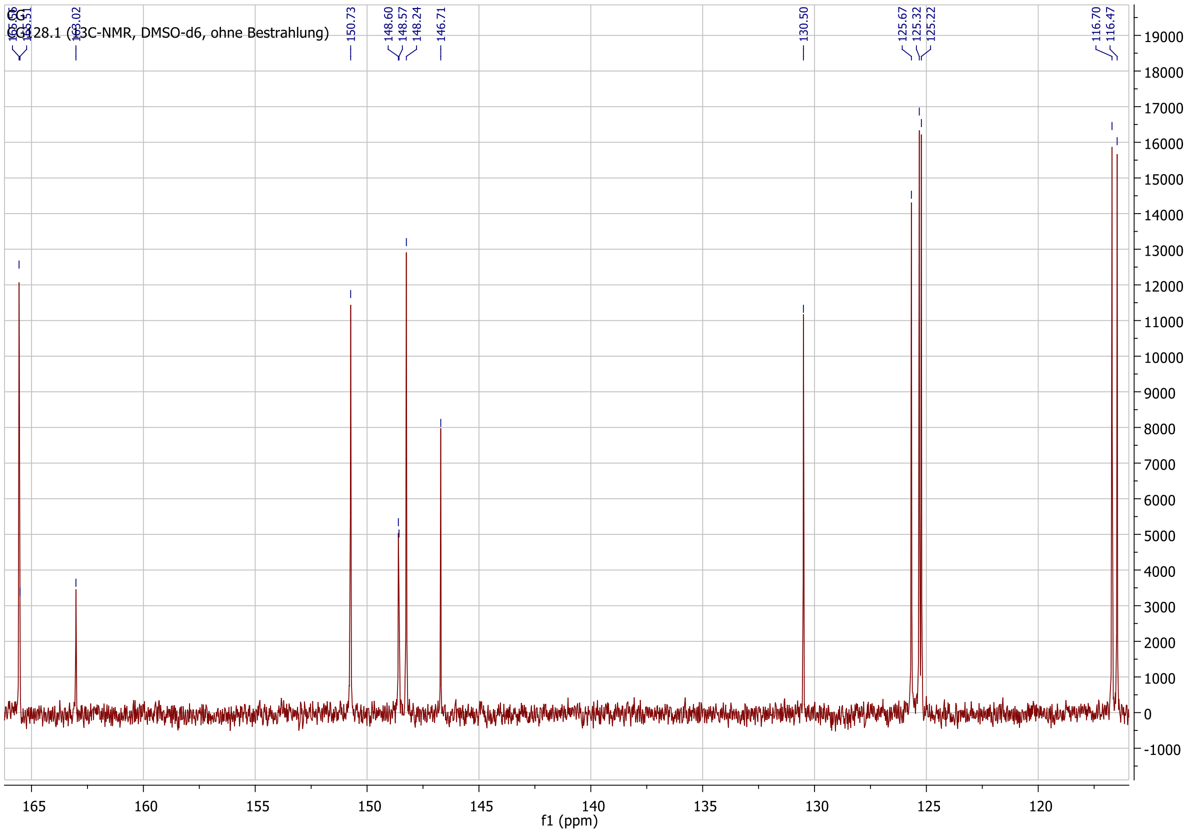

Benzo[*h*]quinoline-3-carboxamide (**8a**)

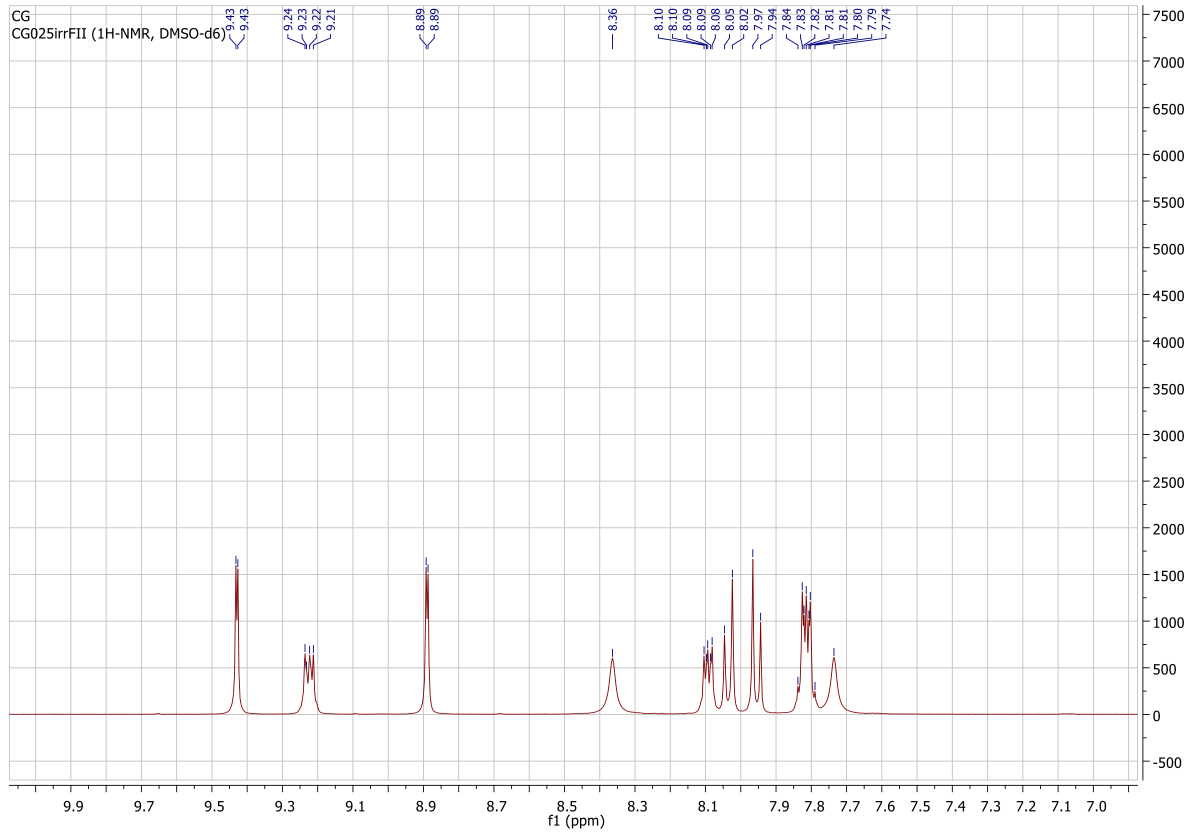

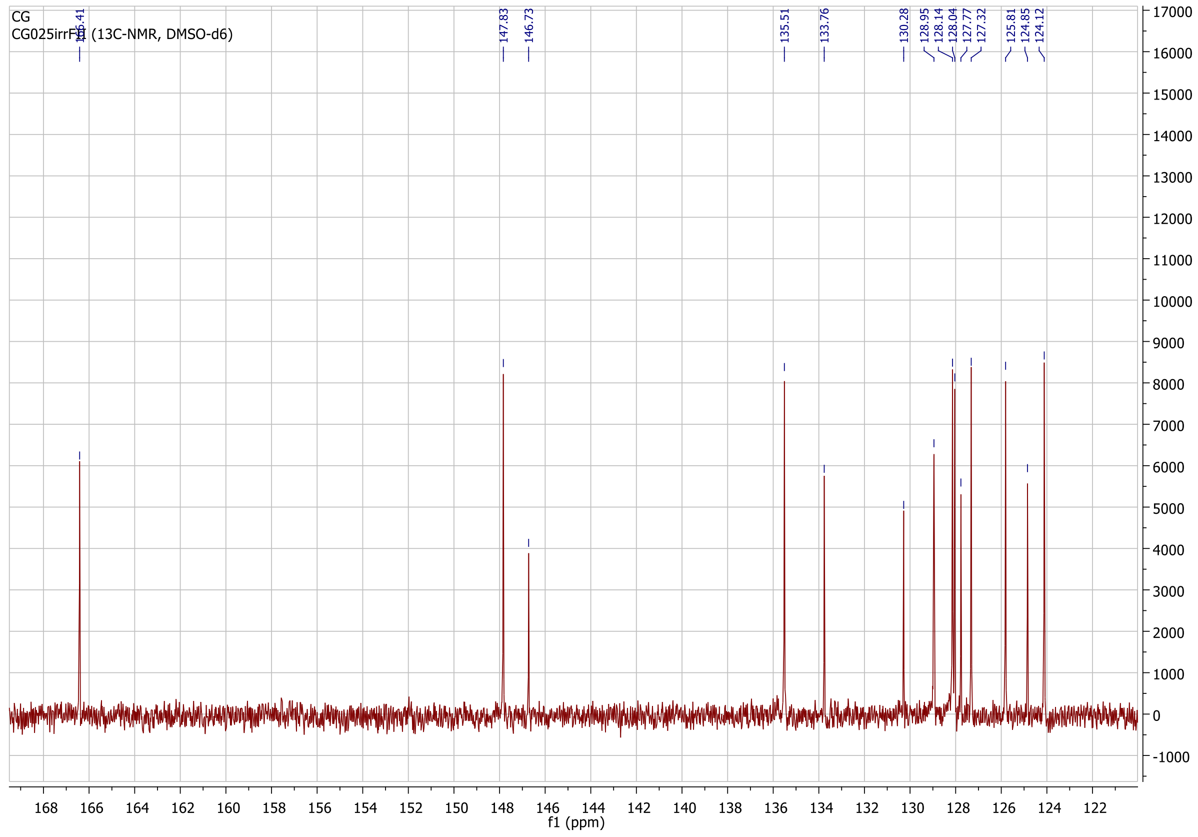

SFC setup

Synthesis was additionally monitored using high speed SFC/MS runs performed by a Nexera SFE-SFC/UHPLC switching system (Shimadzu Corporation, Kyoto, Japan) consisting of a pumping system (one LC-30ADSF for liquid CO2 and two LC-20ADXR for modifier and make-up delivery), an on-line supercritical fluid extraction module (SFE-30A auto extractor equipped with 0.2 mL extraction vessels) for reaction monitoring, an autosampler (SIL-30AC) for purified compounds, a column thermostat (CTO-20AC) equipped with a Torus DIOL (Waters) or Phenomenex CSP (Lux Amylose-2, i-Amylose-3, i-Cellulose-5), a degasser (DGU-20A5R), a communications module (CBM-20A), and two back pressure regulators BPR A and B (SFC-30A) (Figure S1). UV and MS spectra were recorded via photodiode array detection (SPD-M20A) and electrospray ionization single quadrupole MS (LCMS-2020) controlled by Shimadzu LabSolution software (Version 5.91).

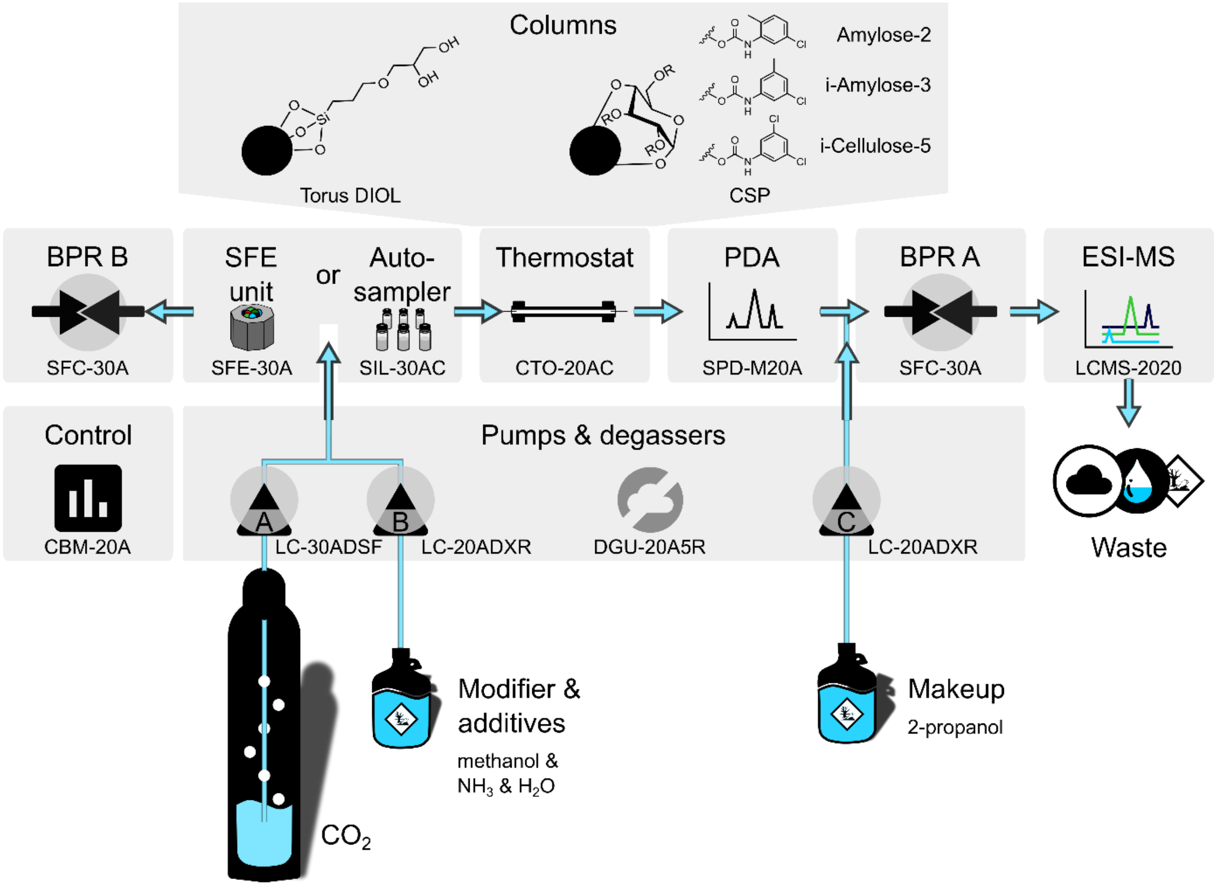

Figure S1: Schematic setup of the super-/subcritical fluid chromatograph. SFE, supercritical fluid extraction; PDA, photo diode array detector; BPR, back pressure regulator; ESI-MS, electrospray ionization-mass spectrometry.

Irradiation setup

Examination of the photochromic properties was conducted at room temperature under total exclusion of daylight using ruby light in a dark room. For all photochemical experiments, spectrophotometric grade solvents and stoppered quartz glass cuvettes (114-QS, Hellma Analytics) were used. UV irradiation was conducted in a Vilber-Lourmat Bio-Link 254 Crosslinker equipped with six Vilber-Lourmat T8-C lamps (8W, 254 nm) or six Vilber-Lourmat T8-L lamps (8W, 365 nm), respectively (Figure S2).

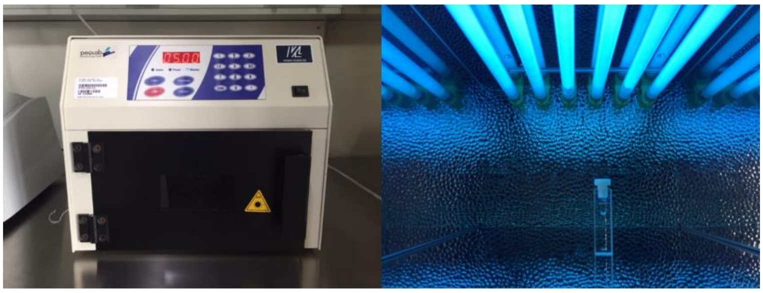

Figure S2: Setup for UV radiation.

As visible light source 1.5 m of a 11 $W \cdot m^{-1}$ RGB-LED strip (Paulmann FlexLED 3D Set, 63 LEDs) wound in a hollow glass cylinder (h = 12 cm, ø 10 cm) were applied. The quartz glass cuvette was put on a slice of aluminium foil and the cylinder placed over it for irradiation (Figure S3).

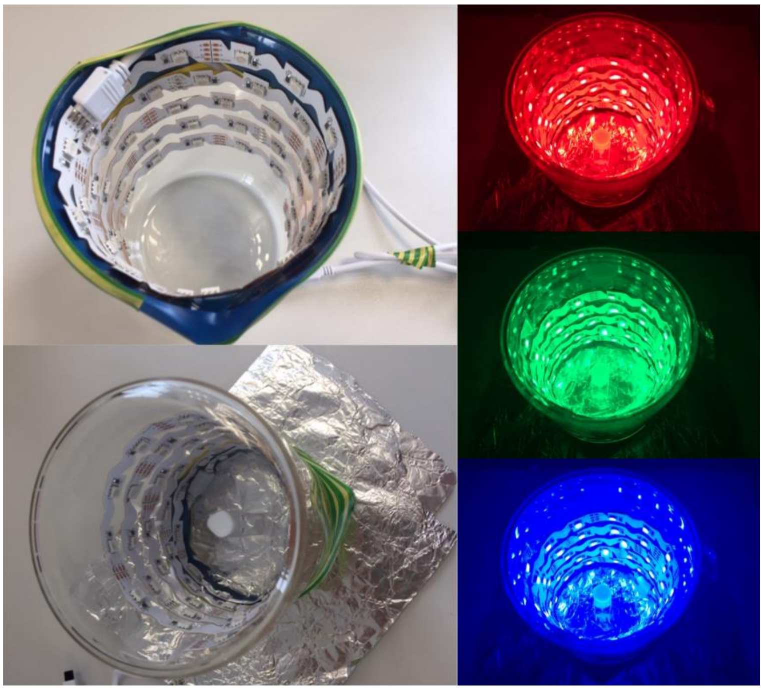

Figure S3: Setup for irradiation with visible light of 630 nm (red), 500 nm (green) or 452 nm (blue).

Determination of isosbestic points of **11**

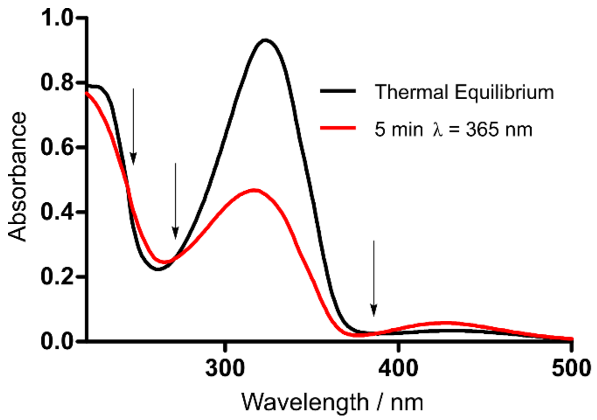

Figure S4: UV–vis spectrum of **11** (50 µM in 60% MeOH (v/v) in water) at the thermal equilibrium and the PSS after 5 minutes of 365 nm radiation. Isosbestic points are assigned by arrows at 244 nm, 270 nm and 387 nm.

PSS composition of **11**

Isomer compositions of the thermal equilibrium and the PSS (365 nm and 452 nm) of **11** were determined via analytical HPLC (Table S1). Therefore 20 µL probes of a methanolic solution

of **11** (1 mM) were injected before and after 5 minutes of 365 nm or 1 minute of 452 nm irradiation. Isocratic elution with 60% MeOH and 40% water (+ 0.1% HCOOH) for 12 minutes at 1 mL·min^{-1} was applied and gave complete separation of the isomers. Integration of the peak areas at the isosbestic points (λ^{obs} = 244 nm and 270 nm) gave the relative percentage of (*E*)-**11** and (*Z*)-**11**, respectively (Figure S5).

Table S1. Isomer composition ((*E*)-**11** / (*Z*)-**11**) at the thermal equilibrium (Δ) and the PSS after 5 minutes of 365 nm and 1 minute of 452 nm radiation.

λ^{obs}	Δ	PSS (365 nm)	PSS (452 nm)
244 nm	98 / 2	17 / 83	76 / 24
270 nm	99 / 1	16 / 84	74 / 26

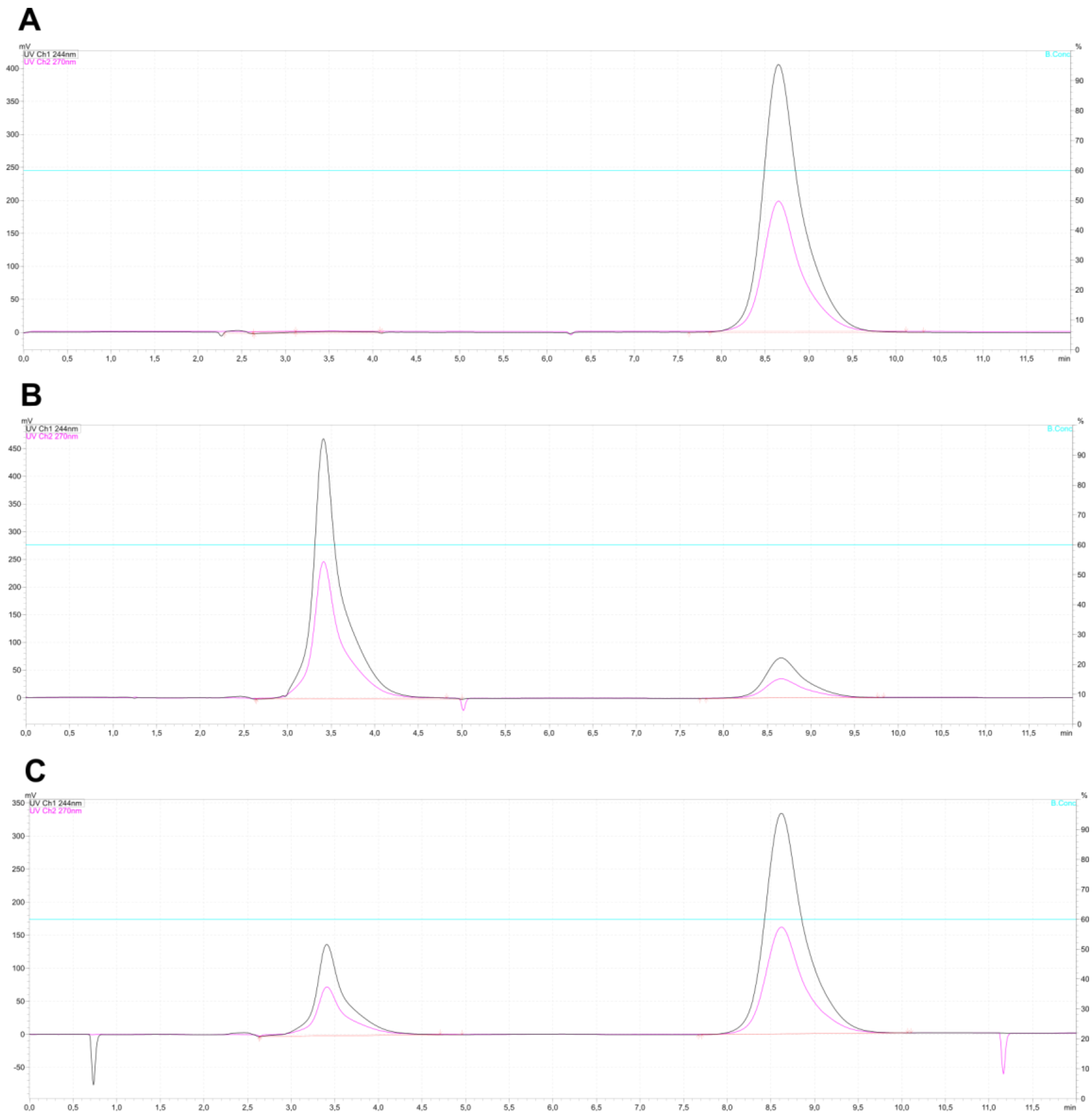

Figure S5: HPLC chromatograms of the thermal equilibrium (A) of **11** and its PSS after 5 minutes of 365 nm (B) and 1 minute of 452 nm radiation (C), recorded using UV–vis detection at the isosbestic points at 244 nm (black) and 270 nm (magenta).

PSS thermal half-life of **11**

The thermal half-life of **11** after 5 minutes of 365 nm irradiation was determined by UV/Vis-spectroscopy. Therefore, a 50 µM solution of **11** in 5% DMSO (v/v) in enzyme assay buffer was prepared and an initial spectrum of the thermal equilibrium recorded. After 5 minutes of irradiation with 365 nm the increase of absorbance at the thermal equilibrium maximum (λ = 324 nm) was followed over a period of 96 hours (Figure S6). Absorbance at 324 nm was plotted as a function of time. A non-linear regression yielded the half-live of the PSS. Due to the slow rate of thermal isomerization only a short period could be measured. Therefore the limit value was set to 0.939 as this was the absorbance at 324 nm of **11** at the thermal equilibrium.

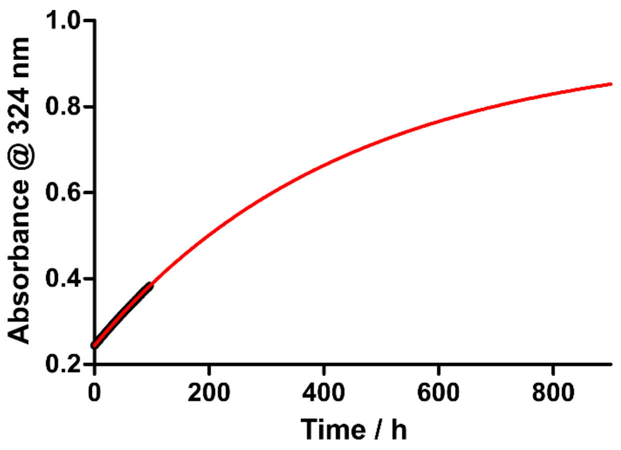

Figure S6: 11 (50 µM) in 5% DMSO (v/v) in enzyme assay buffer after 5 minutes of 365 nm irradiation. Absorbance at 324 nm of plotted as a function of time with non-linear fit (red line).

Table S2: Non-linear fit of measured values.

One phase decay	
Best-fit values	
Y0	0,2437
Plateau	= 0.9390
K	0,002308
Half Life	300,4
Tau	433,4
Span	= -0.6953
Std. Error	
Y0	6,329e-005
K	1,737e-006
Goodness of Fit	
Degrees of Freedom	95
R square	0,9999
Absolute Sum of Squares	8,427e-006
Sy.x	0,0002978
Constraints	
Plateau	Plateau = 0.9390
K	K > 0.0
Number of points	
Analyzed	97

Photochemistry of **2b** and **2f**

LCMS-IT-TOF analysis of **2b**

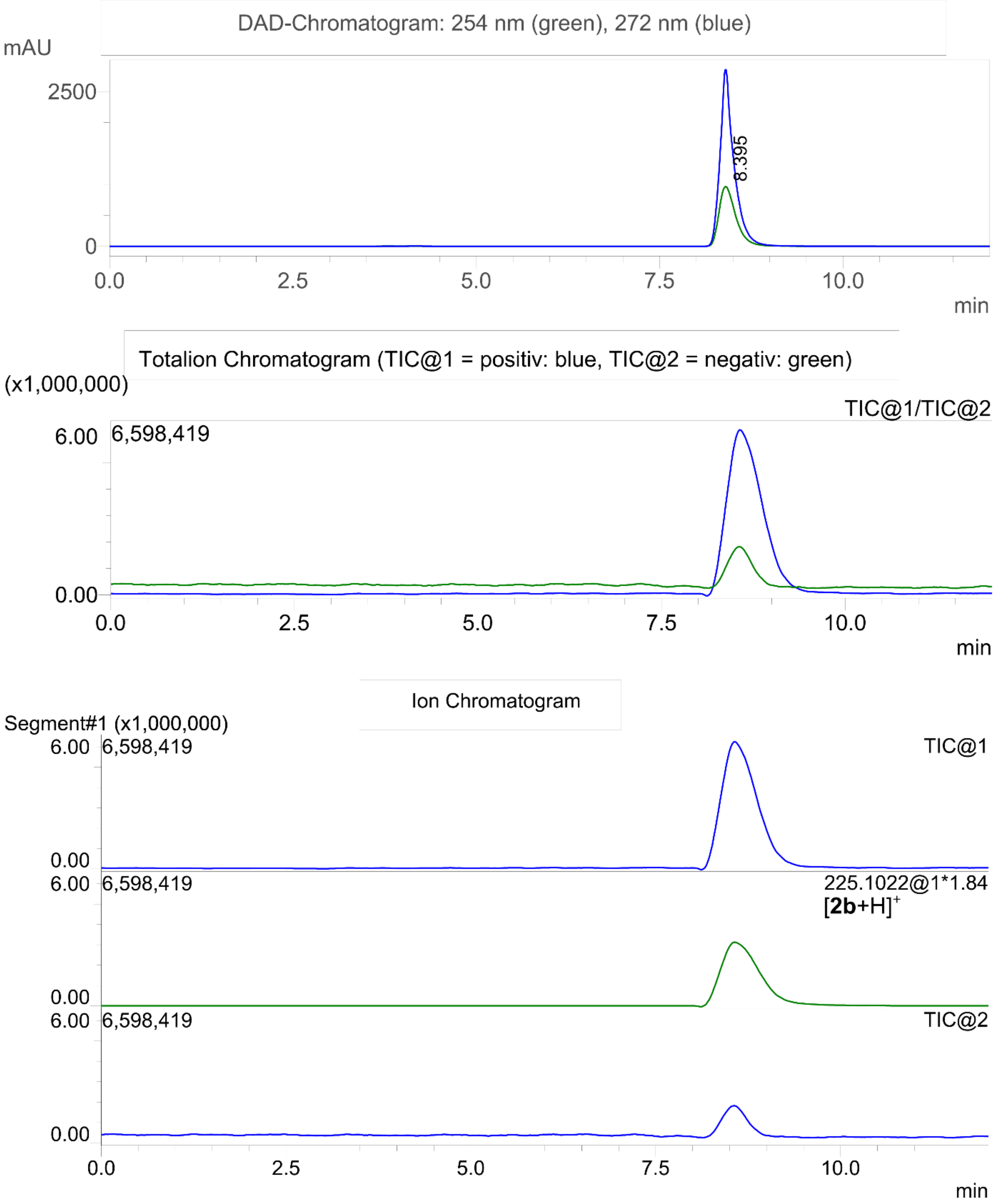

Figure S7: Compound **2b** (10 mM) in methanol at the thermal equilibrium. Injection volume 1 µL, gradient MeOH/H_2O 57–69% (0.1% HCOOH).

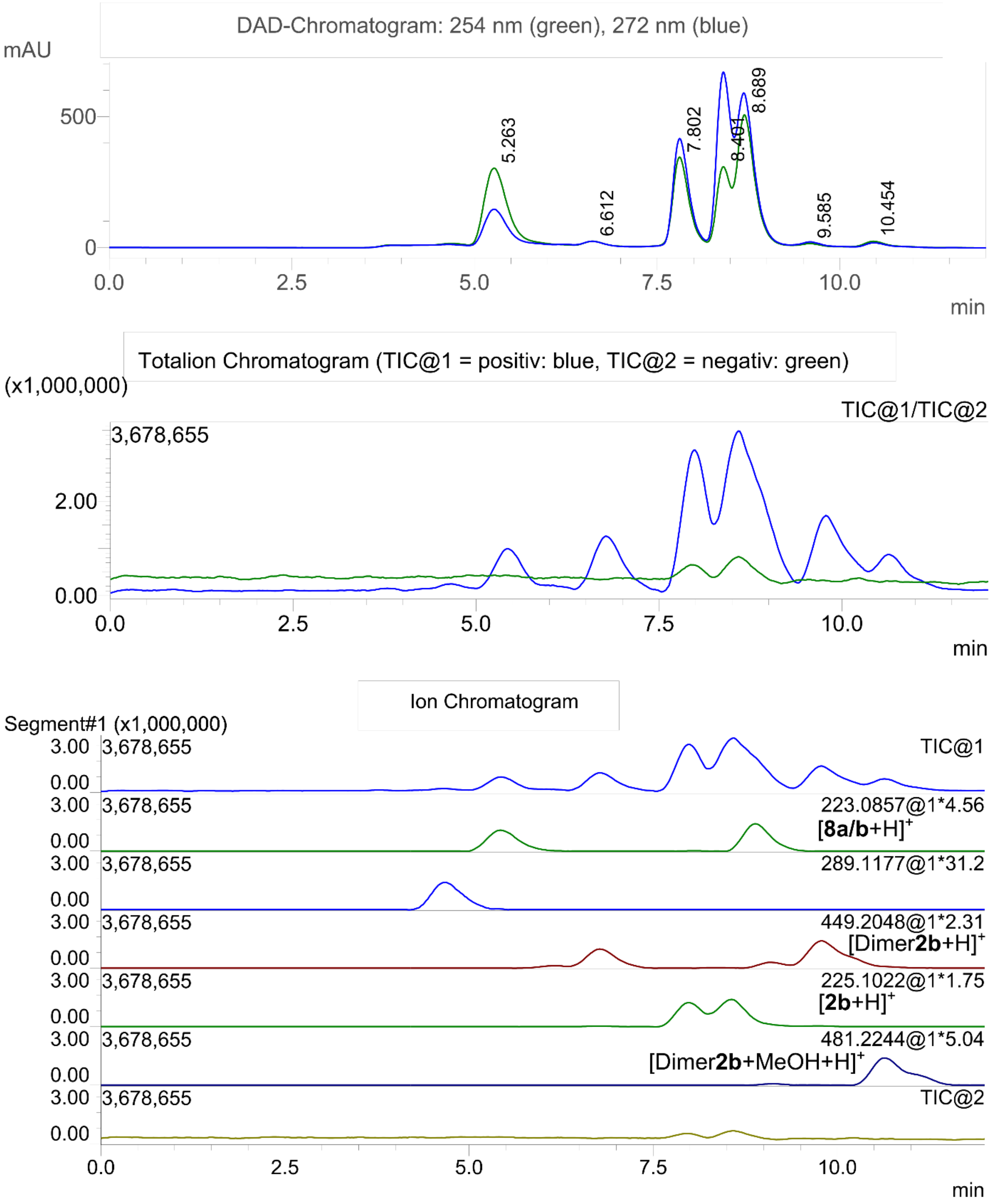

Figure S8: Compound **2b** (10 mM) in methanol after 100 minutes of 254 nm irradiation. Injection volume 1 µL, gradient MeOH/H_2O 57–69 % (0.1 % HCOOH).

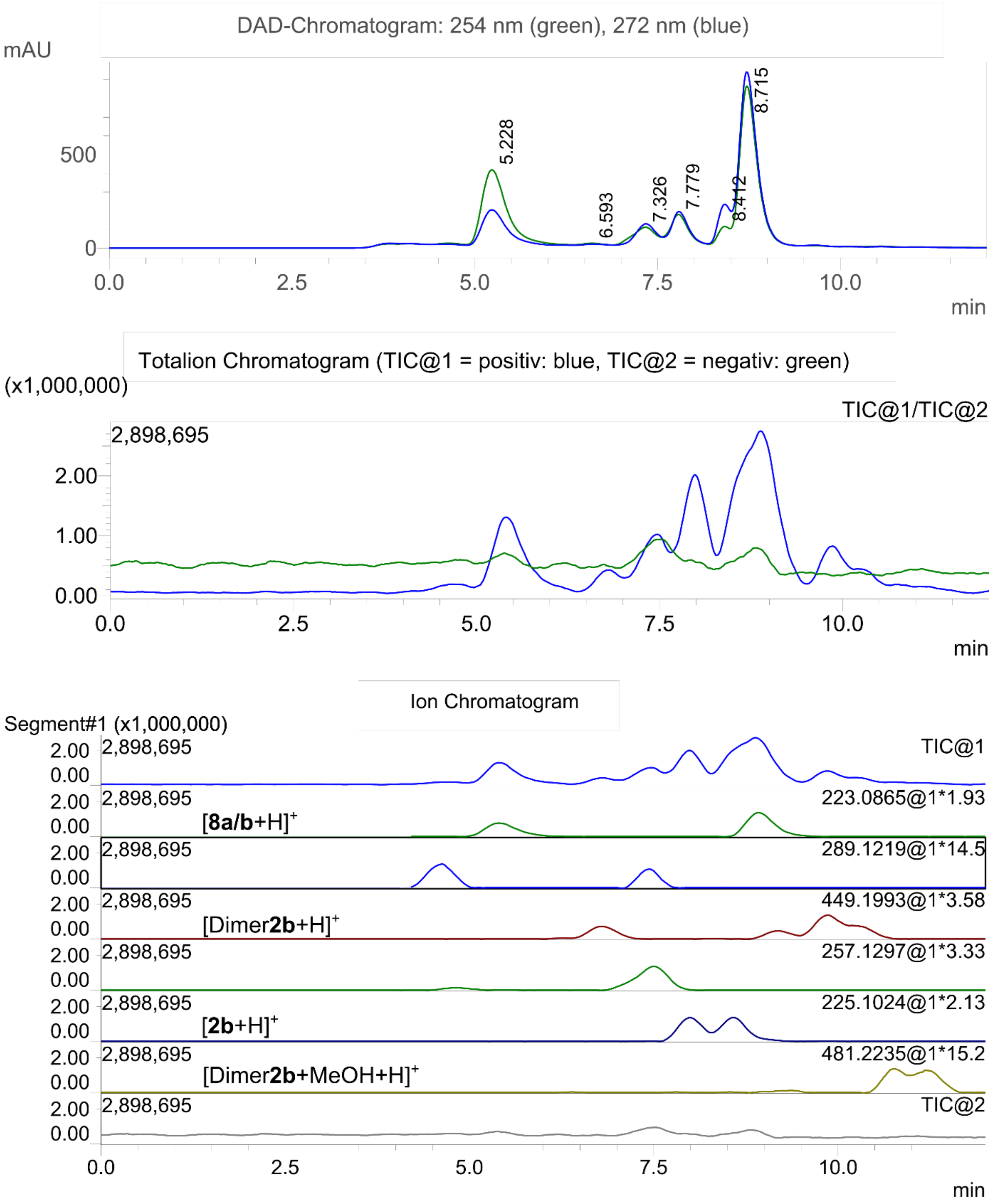

Figure S9: Compound **2b** (10 mM) in methanol after 10 hours of 254 nm irradiation. Injection volume 1 µL, gradient MeOH/H_2O 57–69 % (0.1 % HCOOH).

LCMS-IT-TOF analysis of **8a**

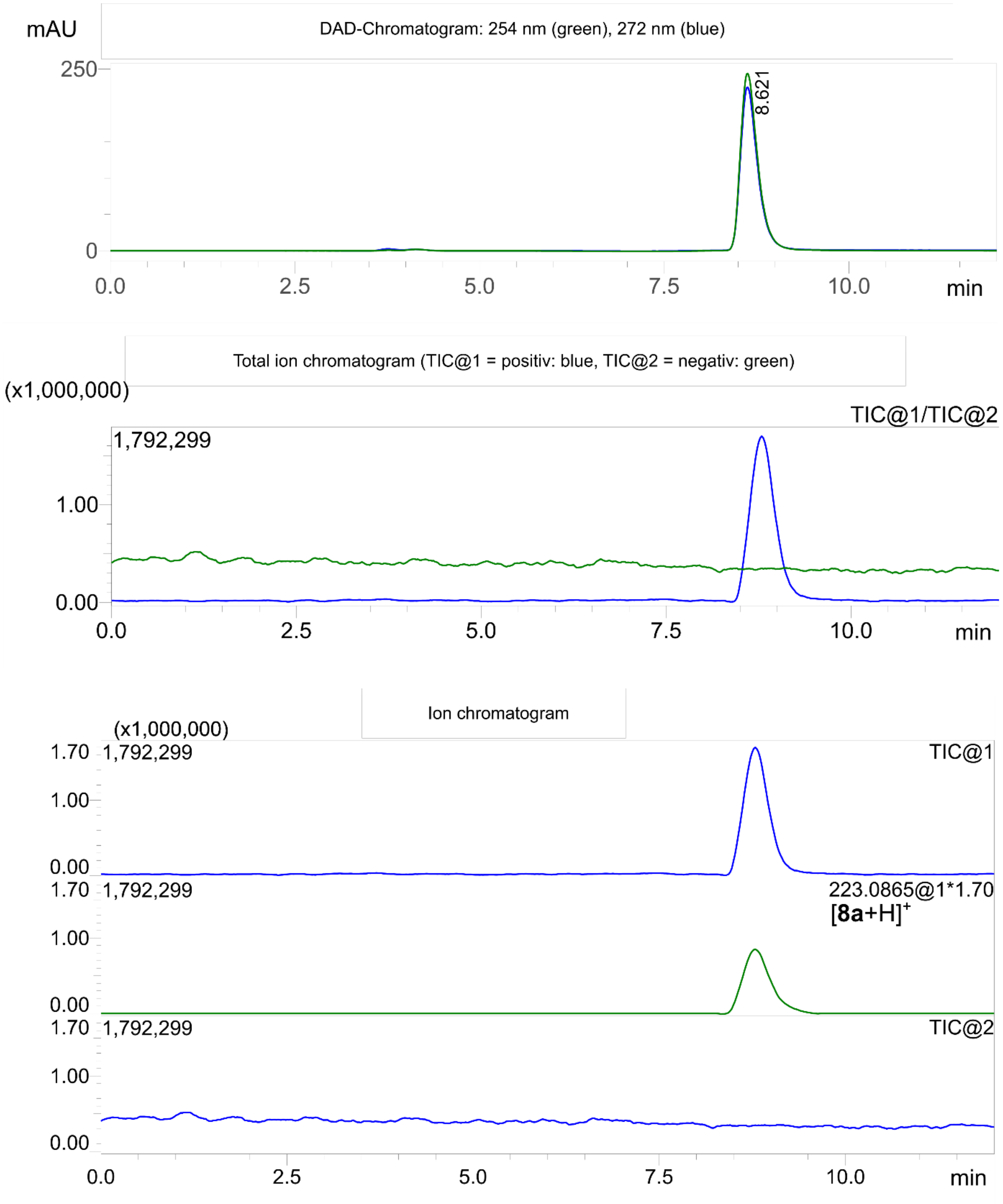

Figure S10: Compound **8a** (10 mM) in methanol. Injection volume 1 µL, gradient MeOH/H_2O 57–69 % (0.1 % HCOOH).

LCMS-IT-TOF Analysis of **2f**

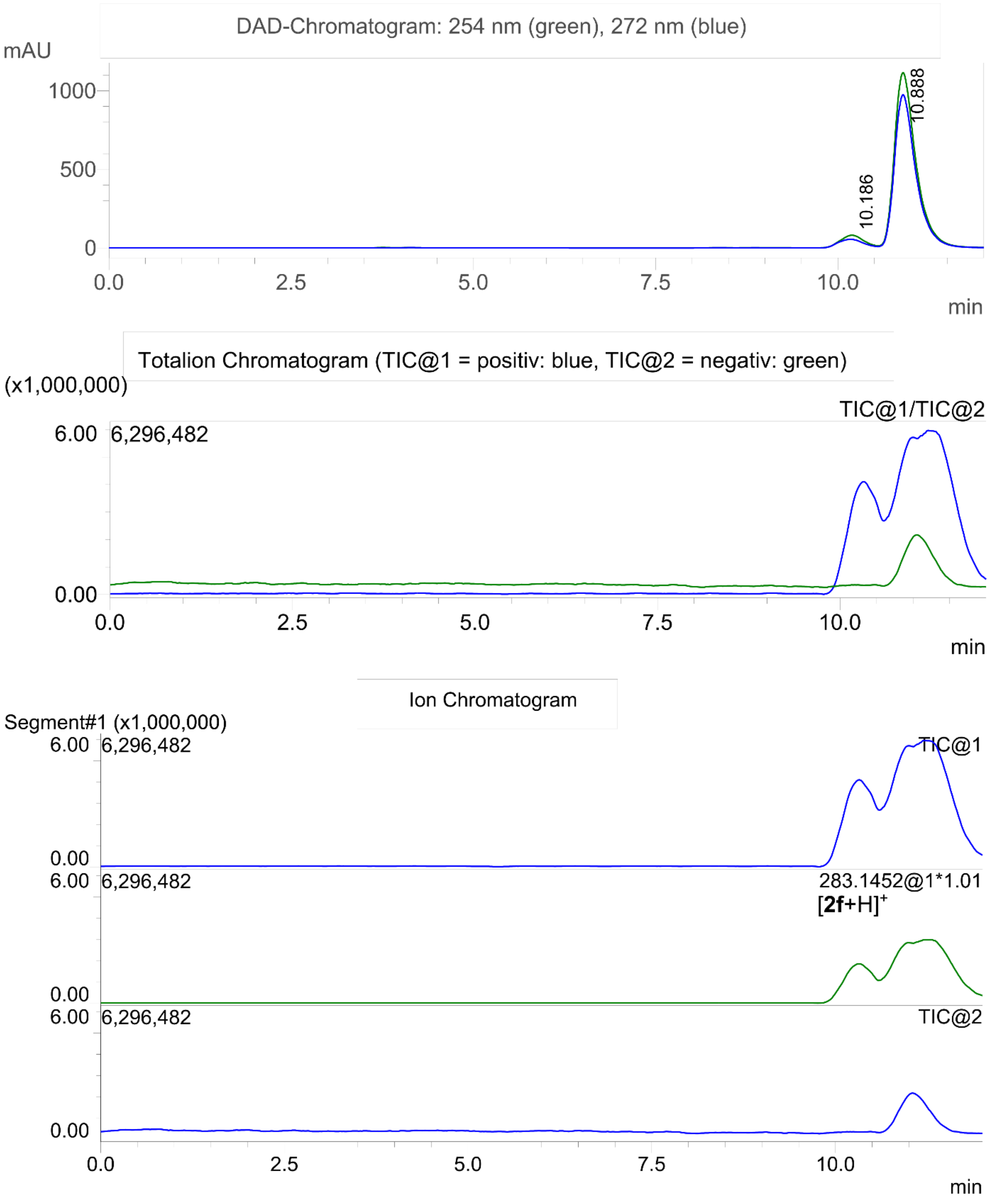

Figure S11: Compound **2f** (10 mM) in methanol. Injection volume 1 µL, gradient MeOH/H_2O 57–69 % (0.1 % HCOOH).

Due to photoisomeriaztion at daylight (*Z*)-**2f** could be observed in the sample of the thermal equilibrium.

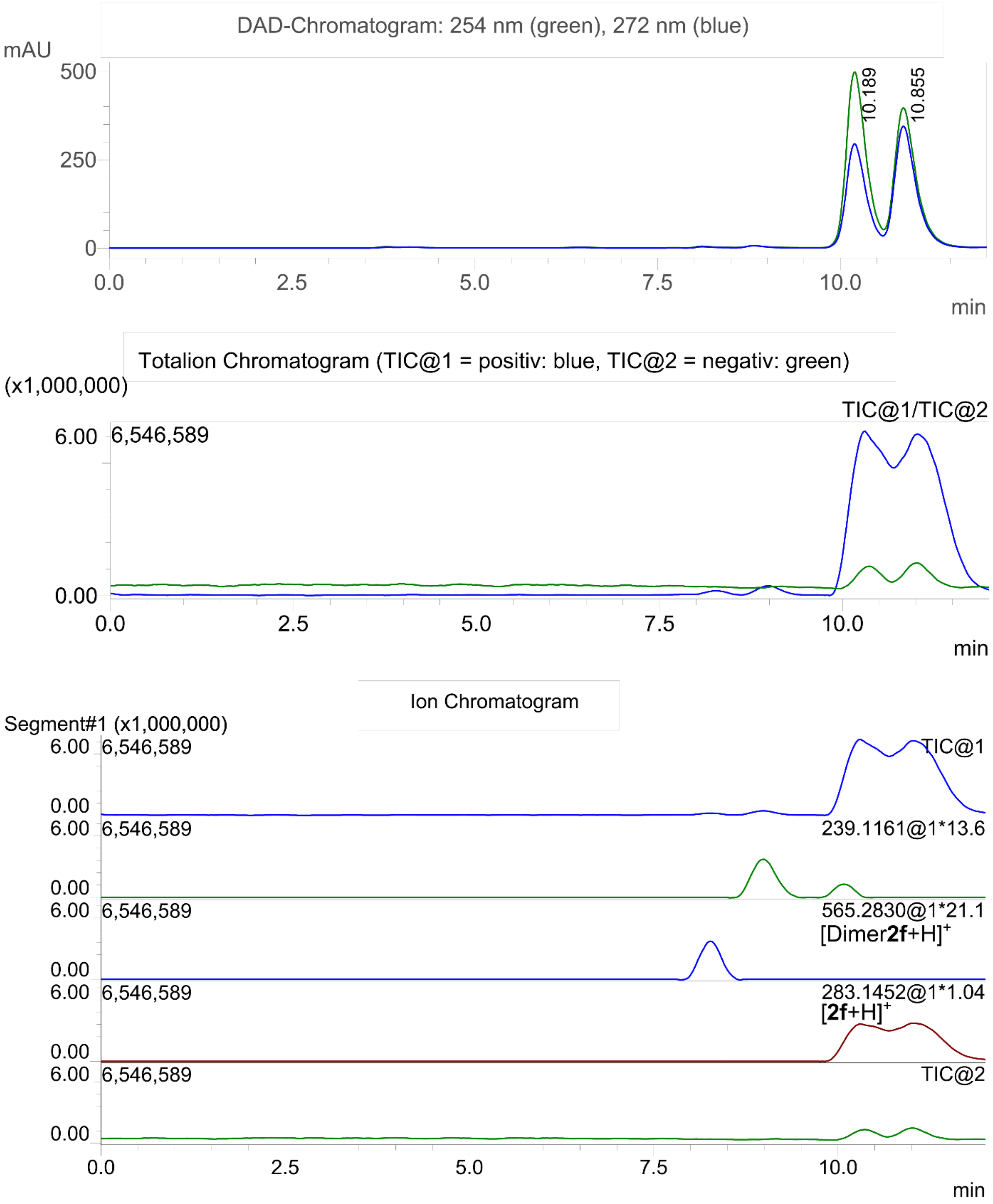

Figure S12: Compound **2f** (10 mM) in methanol after 100 minutes of 254 nm irradiation. Injection volume 1 µL, gradient MeOH/H_2O 57–69 % (0.1% HCOOH).

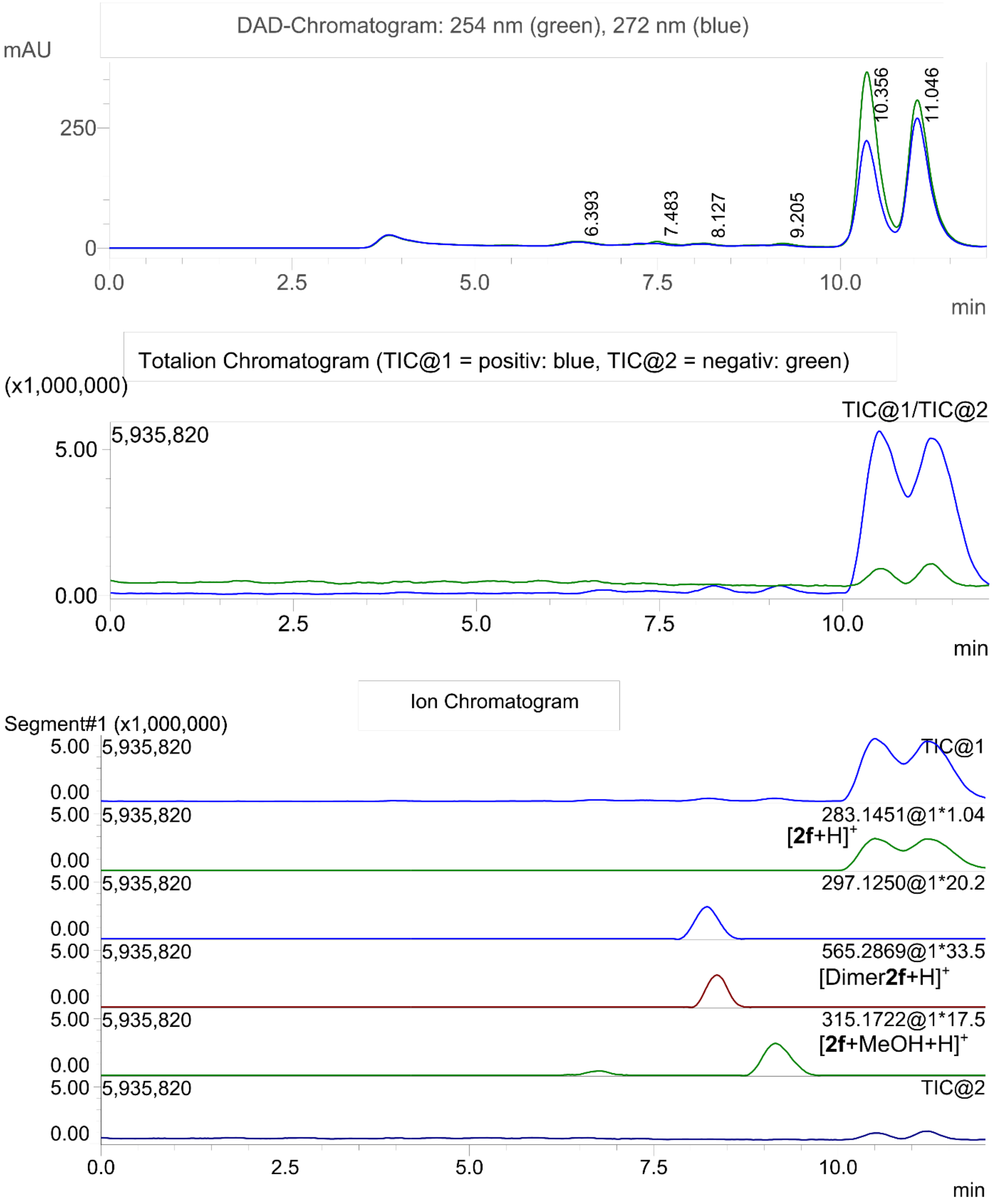

Figure S13: Compound **2f** (10 mM) in methanol after 10 hours of 254 nm irradiation. Injection volume 1 µL, gradient MeOH/H_2O 57–69% (0.1% HCOOH).

^{1}H NMR analysis of **2b**

The relative percentage of (*Z*)-**2b** was determined by comparison of integrals of single proton signals in (*E*)-**2b** and (*Z*)-**2b**. Therefore ^{1}H NMR spectra of **2b** (10 mM) in MeOD were recorded after various irradiation periods with 254 nm.

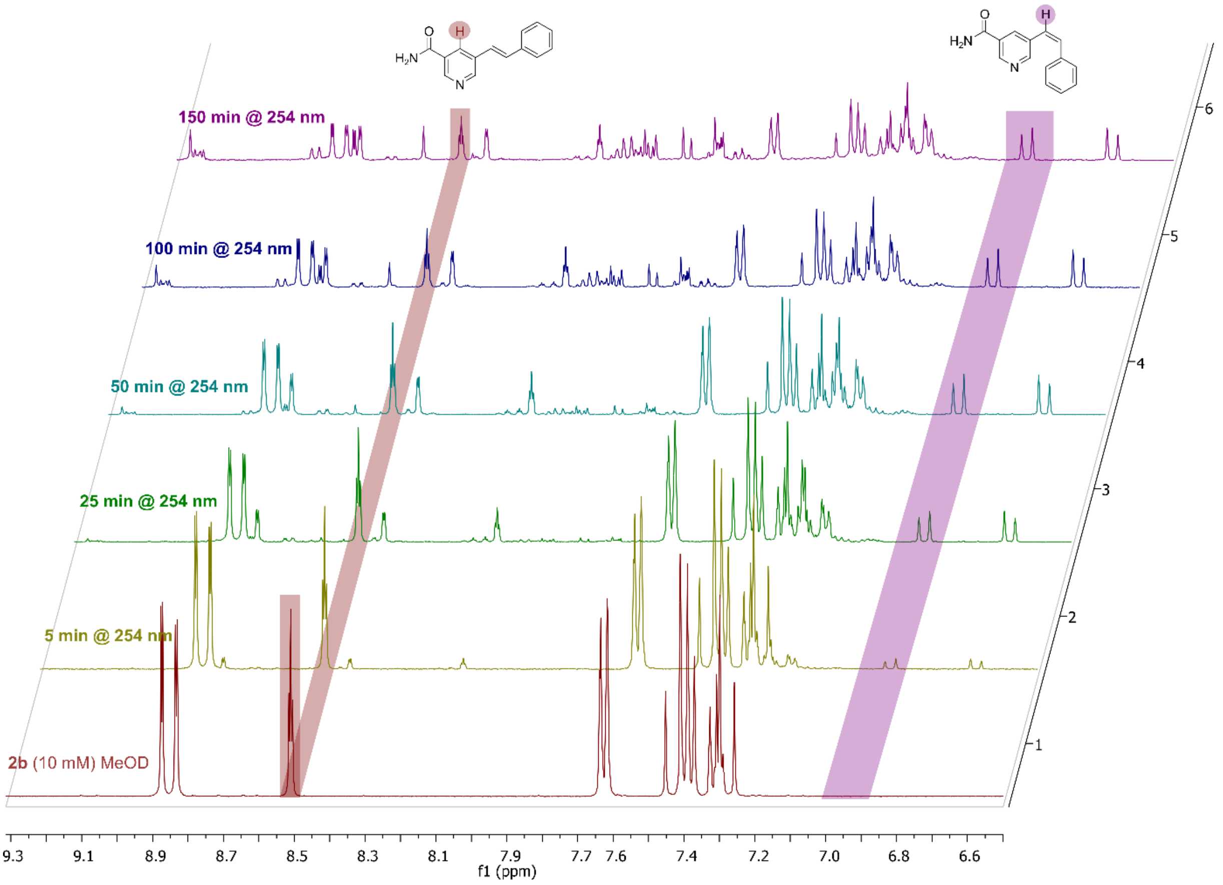

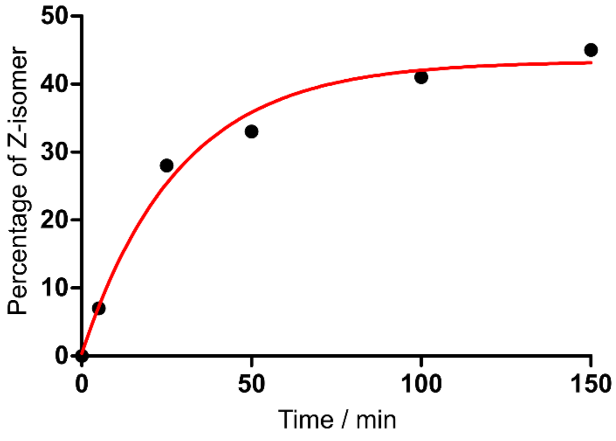

Figure S14: (Above) ^{1}H NMR spectra of **2b** (10 mM) in MeOD after varying irradiation periods. The compared proton signals are highlighted in red and purple. (Below) Relative percentage of (*Z*)-**2b** plotted as function of the irradiation period. A non-linear fit (red line) of the measured values implies a photostationary state comprising about 45% of the (*Z*)-isomer.

^{1}H NMR analysis of **2f**

The relative percentage of (*Z*)-**2f** was determined by comparison of integrals of single proton signals in (*E*)-**2f** and (*Z*)-**2f**. Therefore ^{1}H NMR spectra of **2f** (10 mM) in MeOD were recorded after various irradiation periods with 254 nm.

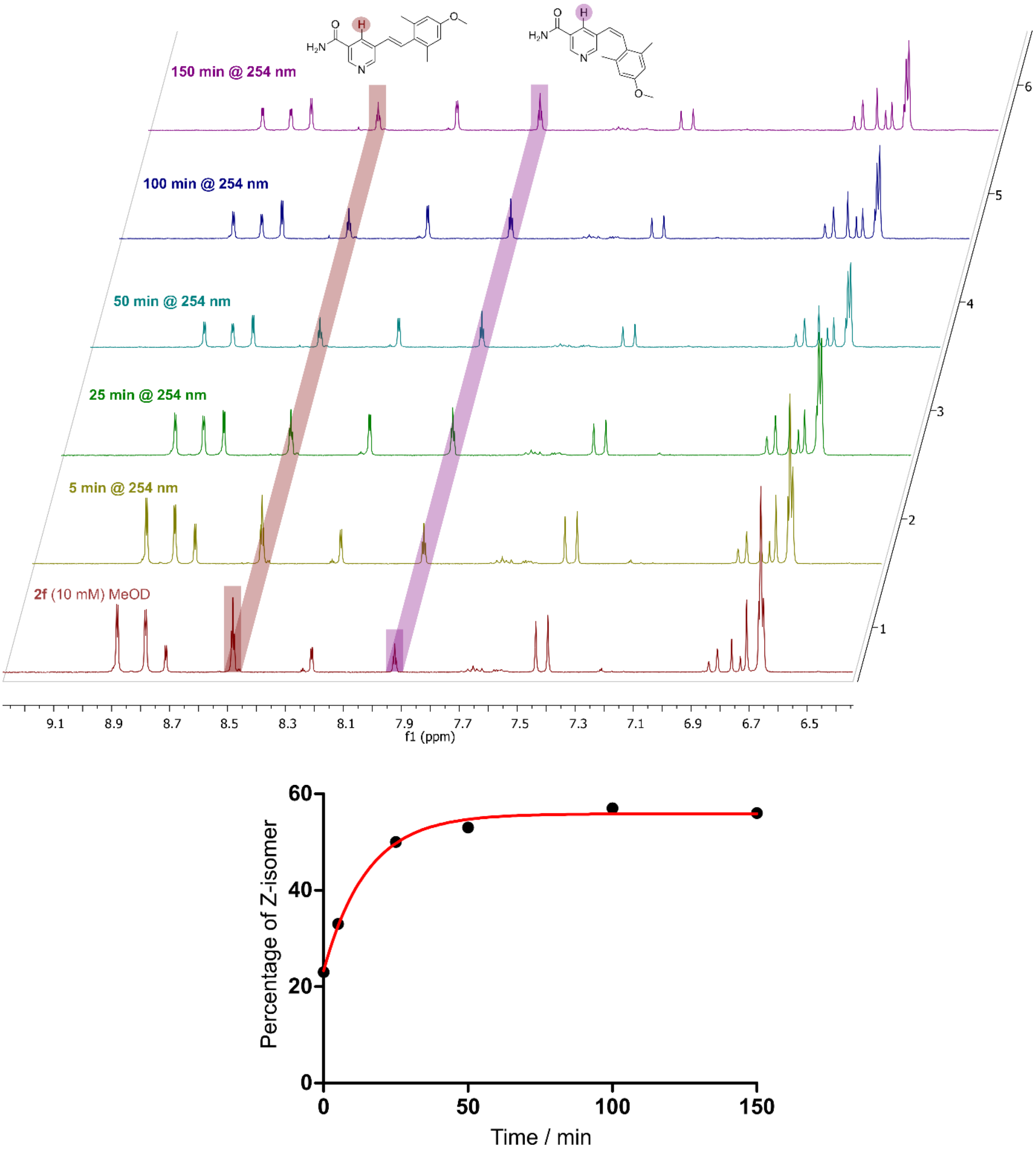

Figure S15: (Above) ^{1}H NMR spectra of **2f** (10 mM) in MeOD after varying irradiation periods. The compared proton signals are highlighted in red and purple. (Below) Relative percentage of (*Z*)-**2f** plotted as function of the irradiation period. A non-linear fit (red line) of the measured values implies a photostationary state comprising 57% of the (*Z*)-isomer. Due to

photoisomerization by daylight *Z*-**2f** was already present in the sample of the thermal equilibrium.

Computational details:

Structures were preoptimized according to TD-DFT using PBE functional and the SVP basis set. The first ten electronic excitations were computed using the PBE0 hybrid-functional, approximate coupled-cluster (CC2) singles-and-doubles model and second-order algebraic diagrammatic construction ADC(2). Excited states were also computed using the larger def2-TZVP basis set. In order to investigate solvent effects on the absorption spectra a continuous solvation model (COSMO) was implemented with permittivity (ε_0) of 62.14 F/m and a refractive index (D_r) of 1.3379, relating to an aqueous solution of 60% methanol (v/v).

Table S2: Summary of the electronic structure calculations. The excitation energies (ω) and oscillator strengths (f) of the ten lowest excited states are given along with λ_{max} values of the lowest two absorption bands. The ground state energies of the DFT/PBE/SVP optimized structure (E_0) is given in Hartree. Δλ refers to the difference of the $\lambda_{max,1}$ values between calculations and experiment.

(*E*)-**2b Experimental** λ_{max} **= 297 nm** (*Z*)-**2b Experimental** λ_{max} **= 282 nm** **8a / 8b Experimental** λ_{max} **= 251 nm**

	Method	Basis	E_0/au	$\lambda_{max,1}$/nm	$\lambda_{max,2}$/nm		ω_1	ω_2	ω_3	ω_4	ω_5	ω_6	ω_7	ω_8	ω_9	ω_{10}
(*E*)-**2b-A**	PBE0	SVP	-724.05552	314	-	ΔE /eV	3.863	4.123	4.259	4.561	4.576	4.757	5.148	5.187	5.205	5.268
						(nm)	(321)	(301)	(291)	(272)	(271)	(261)	(241)	(239)	(238)	(235)
			Δλ =	(+17)	-	f /a.u.	0.72423	0.00007	0.32522	0.00333	0.00118	0.00078	0.00394	0.00076	0.02430	0.00153
		TZVP	-724.85904	320	238	ΔE /eV	3.745	4.139	4.163	4.482	4.604	4.783	5.021	5.144	5.234	5.239
						(nm)	(331)	(300)	(298)	(277)	(269)	(259)	(247)	(241)	(237)	(237)
			Δλ =	(+23)	-	f /a.u.	0.62839	0.40381	0.00004	0.00244	0.00077	0.00092	0.01255	0.01673	0.11804	0.00461
	CC2	SVP	-722.59725	274	205	ΔE /eV	4.446	4.477	4.684	4.771	4.917	5.149	5.896	6.009	6.019	6.141
						(nm)	(279)	(277)	(265)	(260)	(252)	(241)	(210)	(206)	(206)	(202)
			Δλ =	(-23)	-	f /a.u.	0.80094	0.00018	0.35892	0.04366	0.00207	0.00082	0.14331	0.00038	0.02971	0.21350
		TZVP	-723.98836	289	215	ΔE /eV	4.192	4.369	4.480	4.620	4.801	5.017	5.596	5.711	5.805	5.961
						(nm)	(296)	(284)	(277)	(268)	(258)	(247)	(222)	(217)	(214)	(208)
			Δλ =	(-8)	-	f /a.u.	0.72850	0.00010	0.42258	0.02673	0.00118	0.00118	0.07030	0.13438	0.19500	0.09426
	ADC2	SVP	-722.56374	277	209	ΔE /eV	4.389	4.404	4.665	4.775	4.793	5.047	5.856	5.858	6.044	6.055
						(nm)	(282)	(282)	(266)	(260)	(259)	(246)	(212)	(212)	(205)	(205)
			Δλ =	(-20)	-	f /a.u.	0.00037	0.85124	0.37349	0.02833	0.00082	0.00194	0.15514	0.02539	0.10081	0.00822
		TZVP	-723.95026	291	217	ΔE /eV	4.155	4.284	4.462	4.627	4.699	4.932	5.570	5.692	5.723	5.813
						(nm)	(298)	(289)	(278)	(268)	(264)	(251)	(223)	(218)	(217)	(213)
			Δλ =	(-6)	-	f /a.u.	0.77207	0.00013	0.42421	0.01889	0.00035	0.00197	0.11182	0.00036	0.19898	0.09782
	ADC2 / COSMO	SVP	-722.58565	283	207	ΔE /eV	4.314	4.607	4.621	4.794	5.036	5.224	5.783	5.995	6.033	6.110
						(nm)	(287)	(269)	(268)	(259)	(246)	(237)	(214)	(207)	(206)	(203)
			Δλ =	(-14)	-	f /a.u.	0.98271	0.32324	0.00036	0.01197	0.00168	0.00158	0.20860	0.02208	0.19511	0.19125
		TZVP	-723.97454	299	218	ΔE /eV	4.067	4.393	4.526	4.646	4.947	5.115	5.499	5.695	5.781	5.819
						(nm)	(305)	(282)	(274)	(267)	(251)	(242)	(225)	(218)	(214)	(213)
			Δλ =	(+2)	-	f /a.u.	0.87428	0.40536	0.00018	0.00995	0.00097	0.00192	0.12191	0.30675	0.08149	0.13412

	Method	Basis	E_0/au	$\lambda_{max,1}$/nm	$\lambda_{max,2}$/nm		ω_1	ω_2	ω_3	ω_4	ω_5	ω_6	ω_7	ω_8	ω_9	ω_{10}
(E)-**2b-B**	PBE0	SVP	-724.05644	315	236	ΔE /eV	3.922	4.130	4.257	4.494	4.566	4.838	5.025	5.160	5.205	5.291
						(nm)	(316)	(300)	(291)	(276)	(272)	(256)	(247)	(240)	(238)	(234)
			Δλ =	(+18)	-	f /a.u.	0.97037	0.04812	0.00004	0.00172	0.00379	0.00007	0.00070	0.02083	0.01209	0.07566
		TZVP	-724.85988	324	241	ΔE /eV	3.802	4.001	4.315	4.482	4.486	4.880	5.040	5.051	5.151	5.241
						(nm)	(326)	(310)	(287)	(277)	(276)	(254)	(246)	(245)	(241)	(237)
			Δλ =	(+27)	-	f /a.u.	0.84408	0.14473	0.00009	0.00121	0.00310	0.00026	0.01528	0.00044	0.10918	0.00894
	CC2	SVP	-722.59839	276	207	ΔE /eV	4.445	4.619	4.636	4.769	4.796	5.194	5.848	5.904	6.008	6.160
						(nm)	(279)	(268)	(267)	(260)	(259)	(239)	(212)	(210)	(206)	(201)
			Δλ =	(-21)	-	f /a.u.	0.87771	0.00003	0.28846	0.03224	0.00260	0.00026	0.00032	0.10530	0.03073	0.06398
		TZVP	-723.98939	292	215	ΔE /eV	4.183	4.429	4.529	4.618	4.639	5.065	5.596	5.681	5.850	5.956
						(nm)	(296)	(280)	(274)	(268)	(267)	(245)	(222)	(218)	(212)	(208)
			Δλ =	(-5)	-	f /a.u.	0.80796	0.32522	0.00001	0.02255	0.00182	0.00040	0.10265	0.02240	0.13160	0.05045
	ADC2	SVP	-722.56485	278	209	ΔE /eV	4.414	4.508	4.605	4.733	4.773	5.047	5.704	5.868	6.019	6.131
						(nm)	(281)	(275)	(269)	(262)	(260)	(246)	(217)	(211)	(206)	(202)
			Δλ =	(-19)	-	f /a.u.	0.97381	0.00019	0.25069	0.00185	0.02199	0.00076	0.00038	0.11702	0.03866	0.01698
		TZVP	-723.95127	294	216	ΔE /eV	4.155	4.400	4.406	4.614	4.625	4.936	5.513	5.575	5.692	5.858
						(nm)	(298)	(282)	(281)	(269)	(268)	(251)	(225)	(222)	(218)	(212)
			Δλ =	(-3)	-	f /a.u.	0.88297	0.28864	0.00055	0.00137	0.01660	0.00069	0.00020	0.10834	0.03093	0.15236
	ADC2 / COSMO	SVP	-722.58668	285	205	ΔE /eV	4.317	4.560	4.750	4.792	4.936	5.240	5.804	5.994	6.067	6.139
						(nm)	(287)	(272)	(261)	(259)	(251)	(237)	(214)	(207)	(204)	(202)
			Δλ =	(-12)	-	f /a.u.	1.11352	0.17830	0.00016	0.01076	0.00269	0.00045	0.13741	0.02489	0.11039	0.22364
		TZVP	-723.97544	302	216	ΔE /eV	4.067	4.342	4.645	4.665	4.819	5.134	5.518	5.671	5.803	5.857
						(nm)	(305)	(286)	(267)	(266)	(257)	(241)	(225)	(219)	(214)	(213)
			Δλ =	(+5)	-	f /a.u.	1.01263	0.22747	0.00963	0.00017	0.00207	0.00051	0.11822	0.02633	0.31269	0.05494

	Method	Basis	E_0/au	$\lambda_{max,1}$/nm	$\lambda_{max,2}$/nm		ω_1	ω_2	ω_3	ω_4	ω_5	ω_6	ω_7	ω_8	ω_9	ω_{10}
(*Z*)-**2b-A**	PBE0	SVP	-724.04835	301	236	ΔE /eV	3.905	4.242	4.309	4.611	4.674	4.789	5.086	5.185	5.305	5.397
						(nm)	(318)	(292)	(288)	(269)	(265)	(259)	(244)	(239)	(234)	(230)
			Δλ =	(+19)	-	f /a.u.	0.21840	0.10211	0.10672	0.01557	0.06434	0.00059	0.01084	0.01748	0.13903	0.01899
		TZVP	-724.85213	340	306	ΔE /eV	3.826	4.186	4.288	4.598	4.629	4.811	4.995	5.178	5.211	5.329
						(nm)	(324)	(296)	(289)	(270)	(268)	(258)	(248)	(239)	(238)	(233)
			Δλ =	(+58)	-	f /a.u.	0.20225	0.23229	0.00228	0.02157	0.04793	0.00364	0.00947	0.13223	0.03085	0.06130
	CC2	SVP	-722.59511	265	208	ΔE /eV	4.435	4.680	4.681	4.873	4.943	5.132	5.887	5.967	6.052	6.116
						(nm)	(280)	(265)	(265)	(254)	(251)	(242)	(211)	(208)	(205)	(203)
			Δλ =	(-17)	-	f /a.u.	0.08934	0.22340	0.13837	0.00078	0.07236	0.01203	0.31794	0.04329	0.07187	0.13613
		TZVP	-723.98557	278	218	ΔE /eV	4.283	4.491	4.516	4.740	4.806	5.007	5.611	5.685	5.819	5.852
						(nm)	(289)	(276)	(275)	(262)	(258)	(248)	(221)	(218)	(213)	(212)
			Δλ =	(-4)	-	f /a.u.	0.14460	0.31415	0.00309	0.00643	0.05377	0.01447	0.30510	0.05796	0.07233	0.09701
	ADC2	SVP	-722.56167	266	209	ΔE /eV	4.363	4.605	4.669	4.843	4.888	5.005	5.817	5.942	5.988	6.089
						(nm)	(284)	(269)	(266)	(256)	(254)	(248)	(213)	(209)	(207)	(204)
			Δλ =	(-16)	-	f /a.u.	0.05282	0.30004	0.05241	0.09509	0.05775	0.00085	0.21407	0.26524	0.01436	0.12439
		TZVP	-723.94738	279	219	ΔE /eV	4.234	4.415	4.500	4.726	4.754	4.889	5.573	5.696	5.733	5.822
						(nm)	(293)	(281)	(276)	(262)	(261)	(254)	(222)	(218)	(216)	(213)
			Δλ =	(-3)	-	f /a.u.	0.10422	0.23186	0.12173	0.03364	0.06330	0.00193	0.29909	0.09713	0.08122	0.09946
	ADC2 / COSMO	SVP	-722.58239	270	211	ΔE /eV	4.445	4.652	4.720	4.895	5.060	5.198	5.812	5.962	6.020	6.108
						(nm)	(279)	(267)	(263)	(253)	(245)	(239)	(213)	(208)	(206)	(203)
			Δλ =	(-12)	-	f /a.u.	0.23362	0.33522	0.01272	0.01784	0.03635	0.00523	0.48647	0.06598	0.03453	0.16803
		TZVP	-723.97039	282	221	ΔE /eV	4.239	4.456	4.611	4.759	4.963	5.091	5.554	5.661	5.768	5.892
						(nm)	(292)	(278)	(269)	(261)	(250)	(244)	(223)	(219)	(215)	(210)
			Δλ =	(0)	-	f /a.u.	0.23297	0.34983	0.00541	0.02477	0.02602	0.00967	0.44899	0.08523	0.02794	0.09866

	Method	Basis	E_0/au	$\lambda_{max,1}$/nm	$\lambda_{max,2}$/nm		ω_1	ω_2	ω_3	ω_4	ω_5	ω_6	ω_7	ω_8	ω_9	ω_{10}
(*Z*)-**2b-B**	PBE0	SVP	-724.04798	313	240	ΔE /eV	3.917	4.173	4.297	4.485	4.583	4.851	4.953	5.152	5.266	5.326
						(nm)	(317)	(297)	(289)	(276)	(271)	(256)	(250)	(241)	(235)	(233)
			Δλ =	(+31)	-	f /a.u.	0.36812	0.03208	0.03620	0.00765	0.01526	0.01329	0.02416	0.04909	0.00433	0.06208
		TZVP	-724.85164	320	243	ΔE /eV	3.819	4.063	4.340	4.479	4.515	4.885	4.944	5.098	5.147	5.220
						(nm)	(325)	(305)	(286)	(277)	(275)	(254)	(251)	(243)	(241)	(238)
			Δλ =	(+38)	-	f /a.u.	0.32917	0.07578	0.01900	0.01359	0.01006	0.00892	0.05432	0.02592	0.01077	0.09139
	CC2	SVP	-722.59473	271	208	ΔE /eV	4.465	4.590	4.700	4.801	4.822	5.173	5.695	5.907	5.985	6.101
						(nm)	(278)	(270)	(264)	(258)	(257)	(240)	(218)	(210)	(207)	(203)
			Δλ =	(-11)	-	f /a.u.	0.32014	0.00496	0.13353	0.07259	0.01588	0.00771	0.08802	0.05240	0.02633	0.13090
		TZVP	-723.98532	285	219	ΔE /eV	4.237	4.453	4.544	4.659	4.679	5.049	5.439	5.669	5.735	5.796
						(nm)	(293)	(278)	(273)	(266)	(265)	(246)	(228)	(219)	(216)	(214)
			Δλ =	(+3)	-	f /a.u.	0.31434	0.06648	0.08031	0.05723	0.00560	0.00847	0.10842	0.03798	0.04153	0.11475
	ADC2	SVP	-722.56126	273	209	ΔE /eV	4.432	4.496	4.647	4.754	4.823	5.025	5.592	5.839	6.006	6.079
						(nm)	(280)	(276)	(267)	(261)	(257)	(247)	(222)	(212)	(206)	(204)
			Δλ =	(-9)	-	f /a.u.	0.29125	0.06884	0.08906	0.11644	0.00861	0.00711	0.06151	0.07585	0.02798	0.13942
		TZVP	-723.94708	287	219	ΔE /eV	4.215	4.377	4.470	4.632	4.683	4.919	5.369	5.601	5.759	5.780
						(nm)	(294)	(283)	(277)	(268)	(265)	(252)	(231)	(221)	(215)	(214)
			Δλ =	(+5)	-	f /a.u.	0.33861	0.00647	0.13208	0.06283	0.00579	0.00807	0.08452	0.06075	0.05902	0.10821
	ADC2 / COSMO	SVP	-722.58175	277	208	ΔE /eV	4.397	4.627	4.739	4.853	4.929	5.209	5.702	5.966	6.021	6.046
						(nm)	(282)	(268)	(262)	(255)	(252)	(238)	(217)	(208)	(206)	(205)
			Δλ =	(-5)	-	f /a.u.	0.43157	0.14604	0.03847	0.00726	0.03924	0.00735	0.11768	0.05007	0.00300	0.22521
		TZVP	-723.96986	291	219	ΔE /eV	4.168	4.434	4.644	4.713	4.823	5.106	5.446	5.705	5.764	5.821
						(nm)	(297)	(280)	(267)	(263)	(257)	(243)	(228)	(217)	(215)	(213)
			Δλ =	(+9)	-	f /a.u.	0.39004	0.19800	0.00949	0.00795	0.03184	0.00986	0.12568	0.16247	0.09143	0.03185

	Method	Basis	E_0	$\lambda_{max,1}$	$\lambda_{max,2}$		ω_1	ω_2	ω_3	ω_4	ω_5	ω_6	ω_7	ω_8	ω_9	ω_{10}
			-722.90765	256	-	ΔE /eV	3.829	4.168	4.215	4.519	4.693	4.694	4.869	5.191	5.194	5.288
		SVP				(nm)	(324)	(297)	(294)	(274)	(264)	(264)	(255)	(239)	(239)	(234)
	PBE0		Δλ =	(+5)	-	f /a.u.	0.02786	0.00366	0.00085	0.00058	0.13971	0.03097	0.88442	0.00305	0.00034	0.03660
			-723.70153	260	-	ΔE /eV	3.743	4.087	4.223	4.538	4.591	4.715	4.757	5.096	5.210	5.262
		TZVP				(nm)	(331)	(303)	(294)	(273)	(270)	(263)	(261)	(243)	(238)	(236)
			Δλ =	(+9)	-	f /a.u.	0.03057	0.00105	0.00058	0.00052	0.19608	0.00008	0.83793	0.00165	0.25061	0.00007
			-721.48142	237	-	ΔE /eV	3.989	4.481	4.701	4.775	5.033	5.097	5.193	5.289	5.891	5.951
		SVP				(nm)	(311)	(277)	(264)	(260)	(246)	(243)	(239)	(234)	(210)	(208)
	CC2		Δλ =	(-14)	-	f /a.u.	0.01663	0.00125	0.00089	0.00068	0.00004	0.45836	0.24672	0.66837	0.42683	0.00017
			-722.84989	249	-	ΔE /eV	3.840	4.329	4.475	4.659	4.870	4.897	4.936	5.090	5.589	5.758
		TZVP				(nm)	(323)	(286)	(277)	(266)	(255)	(253)	(251)	(244)	(222)	(215)
8a			Δλ =	(-2)	-	f /a.u.	0.02117	0.00082	0.00209	0.00059	0.41586	0.00002	0.51381	0.34514	0.42465	0.00000
			-721.44731	240	-	ΔE /eV	3.973	4.369	4.659	4.705	4.884	5.069	5.179	5.242	5.794	5.885
		SVP				(nm)	(312)	(284)	(266)	(264)	(254)	(245)	(239)	(237)	(214)	(211)
	ADC2		Δλ =	(-12)	-	f /a.u.	0.02176	0.00087	0.00118	0.00096	0.00003	0.54918	0.43686	0.56082	0.00021	0.40210
			-722.81101	251	-	ΔE /eV	3.825	4.237	4.438	4.590	4.774	4.843	4.925	5.057	5.586	5.619
		TZVP				(nm)	(324)	(293)	(279)	(270)	(260)	(256)	(252)	(245)	(222)	(221)
			Δλ =	(0)	-	f /a.u.	0.02728	0.00055	0.00540	0.00083	0.00003	0.57114	0.60919	0.25913	0.44684	0.00004
			-721.46680	246	-	ΔE /eV	3.953	4.533	4.644	4.854	4.978	5.059	5.120	5.222	5.825	5.873
		SVP				(nm)	(314)	(274)	(267)	(255)	(249)	(245)	(242)	(237)	(213)	(211)
	ADC2 / COSMO		Δλ =	(-5)	-	f /a.u.	0.03866	0.00130	0.00432	0.00087	1.20880	0.00008	0.37884	0.23211	0.31815	0.13142
			-722.83547	259	-	ΔE /eV	3.797	4.399	4.421	4.744	4.753	4.867	4.954	5.048	5.515	5.691
		TZVP				(nm)	(327)	(282)	(280)	(261)	(261)	(255)	(250)	(246)	(225)	(218)
			Δλ =	(+8)	-	f /a.u.	0.04641	0.00096	0.01999	1.22099	0.00157	0.33895	0.00001	0.13499	0.37392	0.09314

	Method	Basis	E_0	$\lambda_{max,1}$	$\lambda_{max,2}$		ω_1	ω_2	ω_3	ω_4	ω_5	ω_6	ω_7	ω_8	ω_9	ω_{10}
**8b**	PBE0	SVP	-722.89078	250	-	ΔE /eV	3.891	4.221	4.255	4.615	4.763	4.851	4.951	5.113	5.217	5.299
						(nm)	(319)	(294)	(291)	(269)	(260)	(256)	(250)	(242)	(238)	(234)
			Δλ =	(-1)	-	f /a.u.	0.03094	0.05235	0.00978	0.00438	0.16953	0.09075	0.27448	0.03360	0.14375	0.03961
		TZVP	-723.68618	257	-	ΔE /eV	3.816	4.151	4.301	4.671	4.715	4.781	4.949	5.059	5.151	5.330
						(nm)	(325)	(299)	(288)	(265)	(263)	(259)	(251)	(245)	(241)	(233)
			Δλ =	(+6)	-	f /a.u.	0.03225	0.05429	0.00181	0.03996	0.17085	0.26207	0.11578	0.00779	0.12576	0.00914
	CC2	SVP	-721.47013	232	-	ΔE /eV	3.996	4.536	4.749	4.955	5.091	5.212	5.292	5.367	5.689	5.918
						(nm)	(310)	(273)	(261)	(250)	(244)	(238)	(234)	(231)	(218)	(210)
			Δλ =	(-19)	-	f /a.u.	0.02170	0.00344	0.07335	0.00325	0.07096	0.10272	0.15477	0.66635	0.14430	0.01003
		TZVP	-722.83937	244	-	ΔE /eV	3.859	4.452	4.540	4.854	4.930	5.010	5.074	5.171	5.436	5.757
						(nm)	(321)	(278)	(273)	(255)	(251)	(247)	(244)	(240)	(228)	(215)
			Δλ =	(-7)	-	f /a.u.	0.02465	0.00396	0.06042	0.01110	0.13625	0.08294	0.47861	0.19170	0.14713	0.00502
	ADC2	SVP	-721.43632	234	-	ΔE /eV	3.989	4.476	4.711	4.919	4.991	5.138	5.253	5.313	5.666	5.826
						(nm)	(311)	(277)	(263)	(252)	(248)	(241)	(236)	(233)	(219)	(213)
			Δλ =	(-17)	-	f /a.u.	0.02748	0.00336	0.07327	0.01782	0.05079	0.00019	0.19493	0.85084	0.11332	0.05018
		TZVP	-722.80051	245	-	ΔE /eV	3.852	4.398	4.509	4.821	4.876	4.985	5.022	5.073	5.443	5.663
						(nm)	(322)	(282)	(275)	(257)	(254)	(249)	(247)	(244)	(228)	(219)
			Δλ =	(-6)	-	f /a.u.	0.03139	0.00424	0.06170	0.06297	0.07792	0.06270	0.09489	0.71902	0.15637	0.01611
	ADC2 / COSMO	SVP	-721.45794	239	-	ΔE /eV	3.955	4.676	4.696	5.074	5.169	5.179	5.217	5.329	5.712	6.084
						(nm)	(313)	(265)	(264)	(244)	(240)	(239)	(238)	(233)	(217)	(204)
			Δλ =	(-12)	-	f /a.u.	0.04751	0.09477	0.02823	0.20660	0.22167	0.54466	0.30627	0.06567	0.16639	0.03732
		TZVP	-722.82433	251	-	ΔE /eV	3.811	4.480	4.618	4.879	4.957	4.994	5.098	5.234	5.449	5.889
						(nm)	(325)	(277)	(269)	(254)	(250)	(248)	(243)	(237)	(228)	(211)
			Δλ =	(0)	-	f /a.u.	0.05298	0.10131	0.00315	0.57604	0.50431	0.15088	0.00556	0.01757	0.18629	0.14605

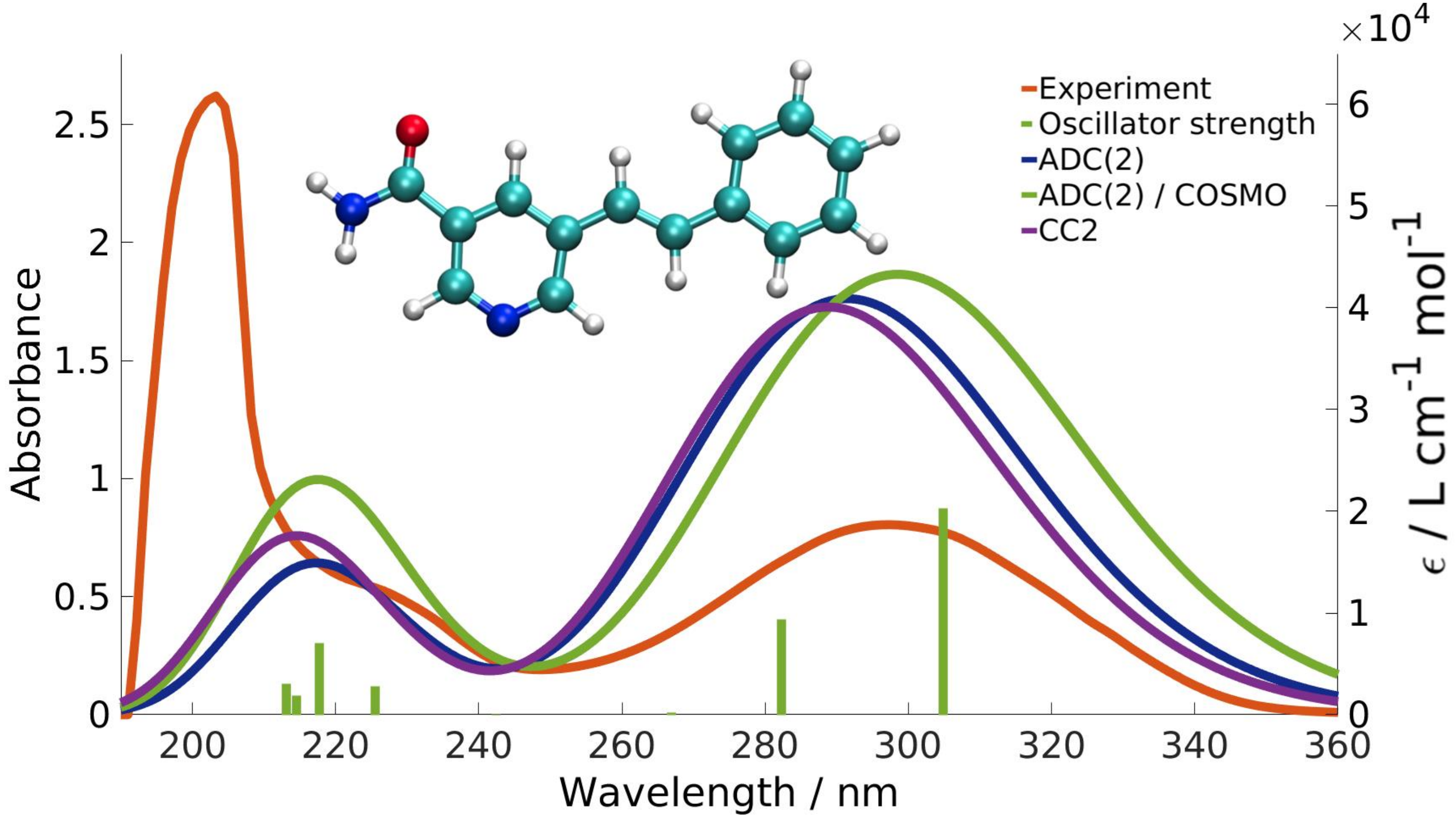

Figure S16: Calculated absorptions spectra of (*E*)-**2b-A** and experimental absorption spectrum of (*E*)-**2b**. Oscillator strengths refer to ADC(2)/COSMO.

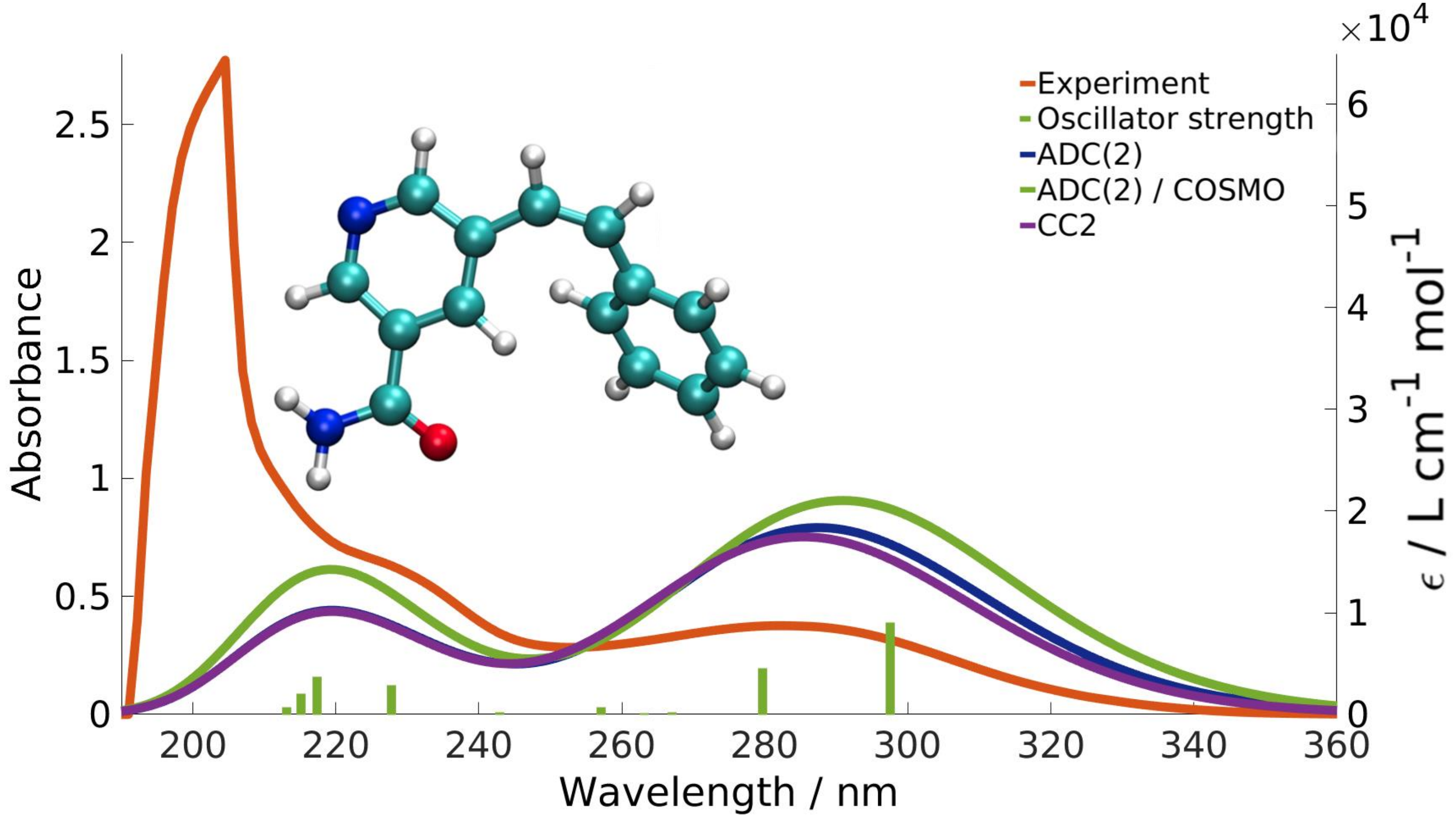

Figure S17: Calculated absorptions spectra of (*Z*)-**2b-B** and experimental absorption spectrum of (*Z*)-**2b**. Oscillator strengths refer to ADC(2)/COSMO.
